# A method for targeting a specified segment of DNA to a bacterial microorganelle

**DOI:** 10.1093/nar/gkac714

**Published:** 2022-08-27

**Authors:** Jan Otoničar, Maja Hostnik, Maja Grundner, Rok Kostanjšek, Tajda Gredar, Maja Garvas, Zoran Arsov, Zdravko Podlesek, Cene Gostinčar, Jernej Jakše, Stephen J W Busby, Matej Butala

**Affiliations:** Department of Biology, Biotechnical Faculty, University of Ljubljana, 1000 Ljubljana, Slovenia; Department of Biology, Biotechnical Faculty, University of Ljubljana, 1000 Ljubljana, Slovenia; Department of Biology, Biotechnical Faculty, University of Ljubljana, 1000 Ljubljana, Slovenia; Department of Biology, Biotechnical Faculty, University of Ljubljana, 1000 Ljubljana, Slovenia; Department of Biology, Biotechnical Faculty, University of Ljubljana, 1000 Ljubljana, Slovenia; Jožef Stefan Institute, Condensed Matter Physics Department, 1000 Ljubljana, Slovenia; Jožef Stefan Institute, Condensed Matter Physics Department, 1000 Ljubljana, Slovenia; Department of Biology, Biotechnical Faculty, University of Ljubljana, 1000 Ljubljana, Slovenia; Department of Biology, Biotechnical Faculty, University of Ljubljana, 1000 Ljubljana, Slovenia; Department of Agronomy, Biotechnical Faculty, University of Ljubljana, 1000 Ljubljana, Slovenia; School of Biosciences, University of Birmingham, Birmingham B15 2TT, UK; Department of Biology, Biotechnical Faculty, University of Ljubljana, 1000 Ljubljana, Slovenia

## Abstract

Encapsulation of a selected DNA molecule in a cell has important implications for bionanotechnology. Non-viral proteins that can be used as nucleic acid containers include proteinaceous subcellular bacterial microcompartments (MCPs) that self-assemble into a selectively permeable protein shell containing an enzymatic core. Here, we adapted a propanediol utilization (Pdu) MCP into a synthetic protein cage to package a specified DNA segment *in vivo*, thereby enabling subsequent affinity purification. To this end, we engineered the LacI transcription repressor to be routed, together with target DNA, into the lumen of a Strep-tagged Pdu shell. Sequencing of extracted DNA from the affinity-isolated MCPs shows that our strategy results in packaging of a DNA segment carrying multiple LacI binding sites, but not the flanking regions. Furthermore, we used LacI to drive the encapsulation of a DNA segment containing operators for LacI and for a second transcription factor.

## INTRODUCTION

Proteinaceous bacterial microcompartments (MCPs) are produced by a variety of bacteria (1). Most MCP shells are selectively permeable and package a specific set of catabolic (metabolosomes) or anabolic (carboxysomes) enzymes, allowing enhanced substrate channelling and protection of the host from toxic pathway intermediates (2). Bacterial MCP shells consist of proteins that oligomerize into homo-hexameric or pseudo-hexameric protein complexes. These assemble into closed icosahedral structures through homo-pentameric shell proteins that form the vertices of the MCP (3). These hexagonal and pentagonal tiles self-assemble, together with cargo enzymes, to form a semipermeable shell (2). Cargo–shell and cargo–cargo interactions in the lumen of the MCP appear to affect the shell size, based on the stoichiometry of the cargo and shell proteins, and the intrinsic curvature of the shell (4,5).

In this work, we repurpose the *Citrobacter freundii* propanediol utilisation (Pdu) MCP to be used as a nucleic acid container. The Pdu metabolosome comprises enzymes for the conversion of 1,2-propanediol (1,2-PD) to propionic acid and propanol (6,7). The Pdu shell is permeable to 1,2-PD, but prevents the escape of toxic propionaldehyde, a product of the first step in 1,2-PD degradation (8,9). The *C. freundii pdu* operon consists of 23 genes, and expression of the genes encoding PduA, B, B' (a truncated form of PduB resulting from an alternative translation site in the *pduB* transcript), J, K, N and PduU, in *Escherichia coli* results in self-assembly of an empty Pdu shell (10,11). Compared to the natural MCP, the synthetic compartment has a smaller diameter and is more robust (10). The major component is PduA, to which selected proteins can be N-terminally fused to present them on the shell surface (11). A heterologous cargo protein can also be encapsulated within the empty Pdu compartment by fusing it to the signal sequences of Pdu enzymes (12,13). Taking advantage of these properties, researchers have been able to redesign the Pdu metabolosome for new purposes, for example, by creating novel nanosized bioreactors (14,15).

*In vivo* encapsulation of specific DNA molecules into the lumen of a MCP shell has not been described, although recent reports describe both repurposed natural non-viral proteins and synthetic icosahedral protein cages that can package RNA molecules *in vivo* (16–18). The ability to create an artificial organelle containing a specified DNA segment could have applications, ranging from the design of gene delivery vehicles to improved nanoreactors in which selected enzymes fused to DNA-binding domains are spatially positioned on a DNA scaffold (19,20). Hence, our major aim in this work was to design a bacterial system for packaging a specific DNA fragment into the lumen of the Pdu shell. We achieved this by arabinose-induced coexpression of the I-SceI nuclease, Pdu shell proteins, and the LacI repressor fused to a Pdu-targeting peptide and enhanced green fluorescent protein (EGFP), which allowed assembly of a synthetic organelle containing the fluorescently labelled LacI–DNA complex. These macromolecular nucleoprotein complexes were subsequently affinity-isolated and the presence of DNA containing multiple LacI operators was confirmed by high-throughput sequencing. To test whether DNA can be used as a scaffold to recruit other proteins to the MCP lumen, we engineered DNA carrying target sites for the LexA repressor (LexA_BT_) of *Bacillus thuringiensis*, flanked by LacI operators. Our data suggest that MCPs can encapsulate DNA with target sites for more than one transcription factor. This paves the way for the design of other synthetic compartments containing DNA.

## MATERIALS AND METHODS

For detailed cloning, primer and sequence information see Supplementary Information. The list of primers, bacterial strains and plasmids used is shown in Supplementary Tables S1–S3.

### Purification of PduD_(1–18)_–LacI–EGFP and LexA_BT_–mCherry for SPR analysis

The BL21(DE3) *E. coli* strain was transformed with the plasmid vector pET21c-lacI or pET21c-lexA and grown on LB plates with ampicillin (100 μg/ml). A single colony was inoculated into 10 ml LB medium and grown at 37°C on a rotating wheel. The overnight culture was diluted 1:50 in 500 ml LB medium. The culture was grown at 37°C to *A*_600_ = 0.6. The medium was cooled and 0.8 mM isopropyl β-D-1-thiogalactopyranoside was added to induce synthesis of PduD_(1–18)_–LacI–EGFP or LexA_BT_–mCherry, both with an C-terminal hexa-histidine tag. The bacterial culture was left growing overnight at 20°C. The bacteria were centrifuged at 6000 × *g* for 15 min (4°C) and stored at -20°C. The bacterial pellet was resuspended at 0.5 g wet weight/ml in lysis buffer (50 mM NaH_2_PO_4_ pH 8.0, 300 mM NaCl, 10 mM imidazole), supplemented with lysozyme (500 μg/ml), DNase (10 μg/ml), RNase (10 μg/ml) and a cocktail of protease inhibitors (Roche, USA). Cells were shaken at 4°C for 30 min and then sonicated on ice at 40% amplitude for 10 min (Vibracell, Sonics, USA). The homogenate was centrifuged at 20 000 × *g* for 30 min (4°C). The supernatant was loaded onto a 500 μl Ni^2+^-NTA column equilibrated in lysis buffer containing 50 mM NaH_2_PO_4_ pH 8.0, 300 mM NaCl and 10 mM imidazole. Weakly bound proteins were eluted with 3 × 10 ml wash buffer (50 mM NaH_2_PO_4_ pH 8.0, 300 mM NaCl, 20 mM imidazole) and the protein bound to affinity resin was eluted in 1.5 ml of elution buffer (50 mM NaH_2_PO_4_ pH 8.0, 300 mM NaCl, 300 mM imidazole). The Ni^2+^-NTA elution buffer was exchanged with buffer containing 20 mM NaH_2_PO_4_ pH 8.0, 140 mM NaCl, 1 mM dithiothreitol (DTT) using dialysis membrane tubing (molecular weight cut-off, 12–14 kDa; Spectrum). Protein samples were stored at -80°C. Prior to SPR analysis, protein samples were centrifuged at 20 000 × *g* for 10 min (4°C) and the concentrations of PduD_(1–18)_–LacI–EGFP and LexA_BT_–mCherry were measured using a spectrophotometer (NanoDrop1000; Thermo Fisher Scientific, USA), with extinction coefficients at 280 nm of 44 810 and 41 830 M^–1^ cm^–1^, respectively.

### Surface plasmon resonance analysis

SPR measurements were performed at the Infrastructural Centre for Analysis of Molecular Interactions at the Department of Biology, University of Ljubljana on a Biacore T100 (GE Healthcare) at 25°C. To prepare double-stranded DNA fragments comprising a single LacI or LexA operator site, complementary primers (Sigma-Aldrich), labelled as in the Supplementary Table S1, were mixed in a molar ratio of 1:1.5 (LacI_u:LacI_d or LexA_u:LexA_d) in 20 mM PBS pH 7.4 and annealed using a temperature gradient from 94°C to 4°C. Approximately 60 resonance units (RU) of the annealed DNA fragments carrying an 15-nucleotide overhang were hybridized to the complementary S1 primer immobilized on flow cell of the sensor chip SA. DNA fragments were injected at a flow rate of 5 μl/min. The interaction of the repressor PduD_(1–18)_–LacI–EGFP or LexA_BT_–mCherry with the target DNA was tested by injecting solutions of the desired protein concentrations (ranging from 1.6 to 200 nM) at 30 μl/min over the DNA immobilized on the chip. Protein was serially diluted in running buffer consisting of 20 mM HEPES pH 7.4, 150 mM NaCl, 3 mM EDTA, 0.1 mg/ml BSA and 0.005% surfactant P20. Each PduD_(1–18)_–LacI–EGFP or LexA_BT_–mCherry concentration tested was injected in two replicates for 120 s at a flow rate of 30 μl/min at 25°C. The dissociation phase was followed for 80–300 s. Experiments were performed in duplicate. Regeneration of the sensor surface was performed with 50 mM NaOH solution for 10 s. The sensorgrams were doubly referenced for the response measured for the control, empty flow-cell and for the response of the buffer. Data were analysed using the Biacore T100 Evaluation Software (GE Healthcare) and equilibrium dissociation constants (*K*_D_s) were determined by fitting the data to the 1:1 Langmuir interaction model. Average *K*_D_s and standard deviations were determined from two titrations of each protein.

### Sodium dodecyl sulphate-polyacrylamide gel electrophoresis

To evaluate the protein composition of the purified MCPs by sodium dodecyl sulphate-polyacrylamide gel electrophoresis (SDS-PAGE), MCPs eluted in the buffer BXT (IBA Lifesciences, Germany) were concentrated by TCA/acetone precipitation. The pellet was resuspended in LDS Sample Buffer (Thermo Fisher Scientific, USA), deionized water and DTT. The protein samples were boiled at 95°C for 5 min and then loaded onto 4–12% gradient gel (Thermo Fisher Scientific, USA) and separated at 120 V for 45 min in MES buffer. The gel was then stained with SimplyBlue SafeStain (Thermo Fisher Scientific, USA) and imaged using G:BOX (Syngene, UK).

### Mass spectrometry

To identify different proteins in SDS-PAGE gels, ∼5 mm gel slices were excised and reduced in 10 mM DTT and cysteines were alkylated by addition of 50 mM iodoacetamide. Proteins were then digested with Trypsin Gold (1.5 ng/μl, Promega, UK) for 12 h at 37°C. Peptides were dried down and then resuspended in 0.1% formic acid solution in water. Peptides were captured on Acclaim PepMap 100 C18 (Dionex, USA) precolumn and separated in Nano Series™ column packed with C18 PepMap100 (Dionex, USA). Peptides from each slice were analysed on the Brunker SolariX XR mass spectrometer using a Triversa Nanomate chip-based electrospray system operated by the University of Birmingham Advanced Mass Spectrometry Facility, UK. The spray voltage of QE HF was set to 1.7 kV through Triversa NanoMate and heated capillary at 275°C. The mass spectrometer performed a full fourier transform mass spectrometry scan (*m*/*z* 360–1600). Full scan mass spectra were recorded at a resolution of 120 000 at *m*/*z* 200 and ACG target of 3 × 10^6^. Precursor ions were fragmented in higher-energy collisional (HCD) MS/MS with resolution set up at 15 000 and a normalized collision energy of 28. ACG target for HCD MS/MS was 1 × 10^5^. The width of the precursor isolation window was 1.2 *m*/*z* and only multiply-charged precursor ions were selected for MS/MS. Spectra were acquired for 56 min.The MS and MS/MS scans were searched against Uniprot database using Protein Discovery 2.2 software, Sequest HT algorithm (Thermo Fisher Scientific, USA). Variable modifications were deamidation (N and Q), oxidation (M) and phosphorylation (S, T and Y). The precursor mass tolerance was 10 ppm and the MS/MS mass tolerance was 0.02 Da. Two missed cleavage was allowed and data were filtered with a false discovery rate of 0.01. Protein with at least two high confidence peptides were accepted as real hit.

### Epifluorescence microscopy

Bacterial cells (5 μl) were fixed on a glass slide coated with poly-l-lysine. Cells were visualized using AxioImager Z1 with ApoTome attachment and Zeiss AxioCam HRc camera (Carl Zeiss, Germany). For EGFP imaging, fluorescence images were acquired using a GFP filter set. Samples were imaged at Infrastructural Centre for Microscopy of Biological Samples (Biotechnical Faculty, University of Ljubljana, Slovenia). The images were processed using the AxioVision program (Carl Zeiss, Germany).

### Measurement of fluorescence spectra

EGFP or mCherry emission spectra were recorded using a home-built fluorescence microspectroscopy (spectral imaging) setup described previously (21). The measuring system is built on an inverted microscope Nikon Eclipse TE 2000-E platform. Fluorescent tags EGFP or mCherry were excited with Xe–Hg source (Sutter Lambda LS, Novato, CA, USA) through a 415–455 nm (EGFP) or 532–557 nm (mCherry) broad-band excitation filter. Fluorescence was detected through a 468–552 nm or 570–743 nm emission filter for the EGFP or mCherry, respectively. For spectral detection, a narrow-band liquid-crystal tunable filter (LCTF; Varispec VIS-10–20 from CRi, Woburn, MA, USA) was placed in front of an EMCCD camera (iXon3 897 from Andor, Belfast, UK), allowing sequential acquisition of images at different wavelengths within the transmission range of the emission filter. Emission spectra were recorded in 5 nm steps in the interval from 470 to 550 nm (EGFP) or from 580 to 740 nm (mCherry) using Andor iXon3 897 EMCCD camera (Andor Technology). Analysis of the spectra was performed using a home-built program developed in MatLab environment (MathWorks, Natick, MA, USA). The samples were prepared by fixing 5 μl of bacterial cells previously supplemented with arabinose for 1.5 h or by fixing 5 μl of MCPs (50 μg/ml) eluted in buffer BXT (IBA Lifesciences, Germany) on a glass slide coated with poly-l-lysine.

### Transmission electron microscopy

A 5 μl drop of purified MCPs in BXT buffer (IBA Lifesciences, Germany) was placed on Formvar coated copper grids. The MCPs were allowed to settle to the grid for 1 min and the remaining suspension was removed from the grid by filter paper. Samples were negatively stained with 1% (w/v) aqueous uranyl acetate (UA) by applying 10 μl UA to the grids for a few seconds. After removal of excessive UA with filter paper the samples were air-dried before observed under CM 100 transmission electron microscope CM 100 (Philips, Eindhoven, The Netherlands) operating at 80 keV.

### Microcompartment expression and purification

Expression of MCPs was performed using *E. coli* strain JW0336-1 as the host. Cells were cultured in M9 minimal salt medium, which allows repression of the P_BAD_ promoter, with the addition of 0.2% glucose, 0.04% MgSO_4_ and, as appropriate, chloramphenicol (25 μg/ml) and tetracycline (12.5 μg/ml). Overnight culture of freshly prepared transformants was diluted 1:50 in 500 ml medium and grown at 37°C to an optical density (*A*_600_) of 0.8. To induce the activity of the *lexA* gene promoter, a subinhibitory concentration of nalidixic acid (6 μg/ml) was added to the cells carying plasmids pRW-LexA and pRW-LexA-mCherry, when the culture reached optical density (*A*_600_) of 0.4 and the cells were left further growing to *A*_600_ of 0.8. Then, 0.4% l-arabinose was added to induce transcription of genes under the P_BAD_ promoter—synthesis of the yeast meganuclease I-SceI, bacteriophage λ Gam protein, Pdu subunits (Twin-Strep-PduA, PduB, -J, -K, -N, -U), and fluorescently labelled LacI. After 1 h, cells were harvested by centrifugation at 8000 × *g* for 15 min (4°C). Pelleted cells were lysed by adding Bacterial Protein Extraction Reagent (Thermo Fisher Scientific, USA) (5 ml per gram of cell debris), lysozyme (100 μg/ml) and an EDTA-free cocktail of protease inhibitors (Roche, USA). DNase I (0.4 U/ml), TURBO™ DNase (0.8 U/ml), 10 mM MgCl_2_ and RNase A (20 μg/ml) were then added (all from Thermo Fisher Scientific, USA). Cells were left at room temperature for 1 h and then centrifuged at 15 000 × *g* for 5 min (4°C). Clear cell lysate was loaded onto the column with pre-loaded 500 μl suspension of Strep-Tactin^®^XT Superflow^®^ High Capacity Agarose Beads (IBA Lifesciences, Germany). To allow the labelled MCPs to bind to Strep-Tactin^®^XT, the mixture was left at room temperature for 1 hour. Subsequently, non-specific binding proteins were washed off with 2 × 10 ml of physiological wash buffer (100 mM Tris–HCl pH 8.0, 150 mM NaCl). Twin-Strep-Tag-bound MCPs were eluted in 1.5 ml BXT buffer (IBA Lifesciences, Germany).

### Immunoprecipitation of EGFP-fusion protein

Half (750 μl) of the MCP sample, purified from the selected bacterial strain, was digested with thrombin (1 U per 120 μg of eluted MCPs) at 20°C for 16 h, and then sonicated for 10 min (Sonis 4, Iskra PIO, Slovenia). Note that thrombin cleavage sites are present in PduA (expecting fragments of 11 and 1.5 kDa) and PduJ (expecting fragments of 8 kDa and 1.2 kDa). Approximately 40 μg of intact and sonicated MCPs were incubated separately with 25 μl suspension of GFP-Trap agarose beads (Chromotek, Germany) using an end-over-end rotation device at 4°C for 1 h to allow binding of EGFP-tagged protein to the agarose beads. As a positive control, 10 μg of purified PduD_(1–18)_–LacI–EGFP diluted in BXT buffer was incubated with GFP-Trap agarose beads. The mixtures were then separated on a column, and the unbound proteins collected in the flow-through fraction were concentrated by TCA/acetone precipitation for subsequent SDS-PAGE and Western blot analysis. The column was washed 4 times with 500 μl 100 mM Tris–HCl (pH 8.0), 150 mM NaCl, 0.5 mM EDTA. After the last wash step, beads were transferred to a fresh tube with low protein binding and centrifuged at 2500 × *g* for 2 min (4°C) and resuspended in LDS Sample Buffer. Samples were boiled at 95°C for 5 min to elute and denature the proteins. Beads were sedimented by centrifugation at 2500 × *g* for 2 min (4°C) and the supernatant was loaded onto 4–20% gradient gel (GenScript, USA) and proteins were separated at 140 V for 65 min in MES buffer.

### Western blot analysis

After SDS-PAGE, proteins were transferred to a PVDF membrane (Thermo Fisher Scientific, USA) in a transfer buffer (20% methanol in 1× SDS buffer) at 150 mA for 90 min. The membrane was incubated in blocking solution (5% skimmed milk in TBST buffer 10 mM Tris–HCl pH 7.4, 150 mM NaCl, 0.05% Tween20) at 4°C with gentle agitation overnight. The membrane was then incubated for 2 h at room temperature with rat anti-GFP primary antibody (Chromotek, Germany) diluted 1:3000 in blocking solution. Unbound antibodies were washed off by three TBST washes for 10 min each. The membrane was incubated with HRP-conjugated goat anti-rat secondary antibody (Thermo Fisher Scientific, USA) diluted 1:10 000 in blocking solution for 2 h at room temperature. Unbound antibodies were washed off by three TBST washes for 10 min each. Labeling was visualized with Amersham ECL Western Blotting Detection Reagent (Cytiva, Germany) using G:BOX.

### DNA purification

Eluted MCPs were treated with DNase I (3 U), TURBO™ DNase (5 U), TURBO™ DNase Buffer and RNase A (20 μg/ml) and incubated at 37°C for 1 h. Then, 15 mM EDTA was added and samples were incubated for 10 min at 37°C. TURBO™ DNase was inactivated by incubating the samples at 75°C for 10 min.

Proteinase K (0.1 mg/ml) (Thermo Fisher Scientific, USA) was added to eluted MCPs previously treated with DNase and RNase to open MCPs and release MCP-associated DNA. Samples were kept at 60°C for 1 h. Ammonium acetate (3.3 M) was added and samples were incubated on ice for 10 min to allow precipitation of proteins from solution. DNA released from the MCPs was then purified by phenol/chloroform/isoamyl alcohol extraction. Glycogen and isopropanol were then added and the mixture was kept on ice for 10 min followed by centrifugation at 16 000 × *g* (4°C) for 10 min. The supernatant was removed and the pellets were resuspended in 80% ethanol to remove any residual salts. The samples were centrifuged at 16 000 × *g* for 10 min (4°C), the supernatant was removed and washing of the pellets with ethanol was repeated twice. Pellets were air dried to remove residual alcohol and then resuspended in 30 μl TE buffer (10 mM Tris–HCl pH 8.0, 0.1 mM EDTA). Samples were treated with RNase A (20 μg/ml) at 37°C for 20 min before agarose gel electrophoresis or prior the high-throughput sequencing analysis was performed.

### High-throughput sequencing

High-throughput sequencing was performed using the Ion Proton NGS sequencing system. Briefly, isolated DNA was sonicated for 3 min in an ultrasonic water bath at room temperature. Sequencing libraries were prepared according to the in-house protocol for DNA library preparation. DNA end-repair of 28 μl (∼0.2 ng/μl) fragmented DNA was performed in 40 μl using T4 polynucleotide kinase and T4 DNA polymerase at 25°C for 25 min, followed by incubation at 12°C for 10 min. The reaction was purified with 1.8 volumes of MagSI-NGSPREP Plus magnetic beads and eluted with 20 μl of dH_2_O. P1 and A adaptors with corresponding barcodes were ligated to the ends of the DNA fragments using T4 DNA ligase and Bst 2.0 WarmStart DNA polymerase. 30 μl reactions were incubated for 30 min at 22°C followed by incubation for 20 min at 50°C. Reactions were purified with 1.8 volumes of MagSI-NGSPREP Plus magnetic beads and eluted with 20 μl of dH_2_O. The library was amplified (50 μl) with 6 cycles using KAPA HiFi HotStart DNA Polymerase and P1amp and T_PCR_A primers (0.5 μM) using the following amplification protocol: initial denaturation 95°C 3 min followed by 6 cycles 98°C 20 s, 60°C 30 s and 72°C 30 s. The PCR reaction was purified with 1.8 volumes of MagSI-NGSPREP Plus magnetic beads and eluted with 20 μl of dH_2_O. Libraries were checked for size and concentrations according to the Agilent High Sensitivity Kit protocol. Equimolar amounts of libraries were pooled together and amplified according to the protocol of the Ion PI Hi-Q OT2 200 kit. Ion Proton System and samples were prepared according to the protocol of the Ion PI Hi-Q Sequencing 200 kit and sequenced on an Ion PI Chip v3. Basecalling was performed from the device pipeline and adapters were removed from the raw sequencing data. The raw sequencing data was submitted to the Sequence Read Archive (SRA) database. Sequencing reads were mapped to the complete genome sequence of *E. coli* BW25113 (accession number CP009273) and the corresponding plasmids pOH, pOHΔPduD, pOHΔTwin-Strep-tag, pOHΔLacI, pRW-(*lacO*)_5_, pRW-(*lacO*)_8_ or pRW-LexA (Supplementary Table S3) using the tool ‘Map Reads to Reference’ implemented in CLC Genomics Server (version 20.0.4) using default settings. The mapping results were exported as BAM files. BEDTools genomecov was used to obtain the coverage per base pair from BAM file (22). The read coverage of each base pair of a plasmid was normalized by subtracting the median read coverage of the chromosome of the selected sample from the read coverage of a base pair and dividing the remainder by the median read coverage of the chromosome. Where indicated, the average read coverage of two biological replicates was used for plotting.

For the samples of strains containing: (i) pRW-(*lacO*)_5_ and pOH, (ii) pRW-(*lacO*)_5_ and pOHΔPduD, (iii) pRW-(*lacO*)_5_ and pOHΔTwin-Strep-tag, (iv) pRW-(*lacO*)_8_ and pOH, (v) pRW-(*lacO*)_8_ and pOHΔLacI, (vi) pRW-LexA and pOH, or (vii) pRW-LexA and pOHΔLacI, we determined the number of reads aligned to the 600 bp segment carrying LacI operator sites and the total number of reads aligned to the plasmids and chromosome using the SAMtools (23). Next, we calculated the average number of reads aligned per 600 bp for each DNA molecule in the sample, by dividing the total number of reads mapped to the plasmid/chromosome by the full length of the plasmid/chromosome and then multiplying by 600. The resulting value was then divided by the total number of mapped reads in the sample and indicates the number of reads that align to a random 600 bp DNA fragment if the distribution of mapped reads was homogeneous throughout the genome. Then, to calculate the relative abundance of reads mapped to the 600 bp carrying LacI or LacI-LexA operator sites of plasmids pRW-(*lacO*)_5_, pRW-(*lacO*)_8_ or pRW-LexA, compared to all the mapped reads in the sample, the absolute number of reads mapped to the fragment was divided by the total number of mapped reads in the sample.

## RESULTS AND DISCUSSION

### Engineering of Strep-tagged Pdu microcompartments containing LacI–EGFP

We previously demonstrated affinity isolation of a nucleoprotein complex excised *in vivo* from a low-copy number plasmid (24). To do this, we constructed a bacterium that produces FLAG-tagged LacI, which binds at five LacI operators cloned upstream of a selected DNA segment inserted into a plasmid. The LacI sites and DNA segment are flanked by 18-bp target sites for the yeast I-SceI meganuclease. Induction of I-SceI nuclease triggers excision of this region from the plasmid and the released DNA is stabilized by co-induced phage Lambda Gam protein, which inhibits host RecBCD nuclease. The LacI–DNA complex could subsequently be purified from the bacterium. Here, we develop this method by using LacI to drive the repressor–DNA complex into the lumen of a Pdu shell, which is then affinity-isolated.

**Figure 1.**
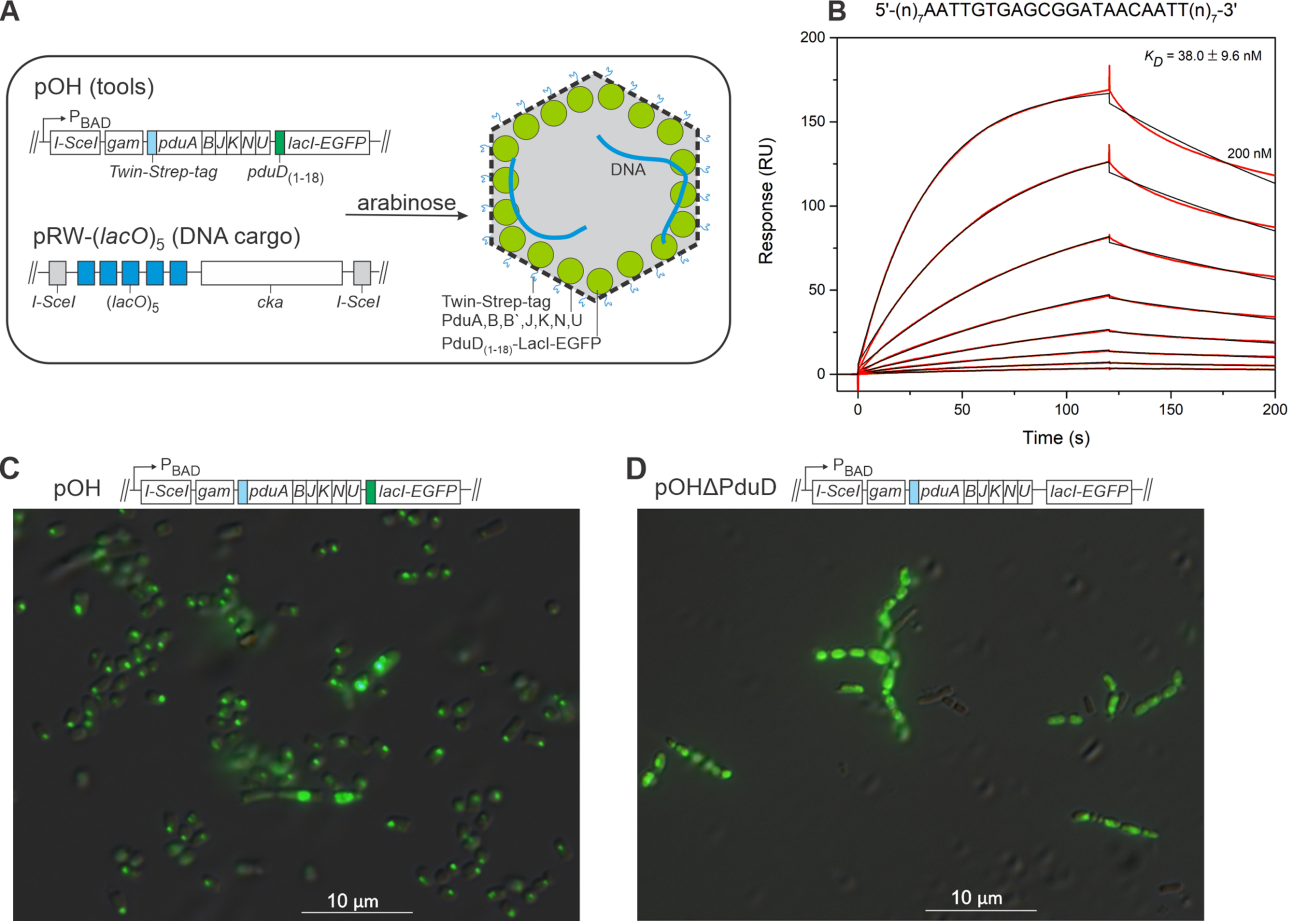
Engineering of the Pdu-LacI microcompartment. (**A**) Schematic representation of parts of plasmids pOH and pRW-(*lacO*)_5_. Expression of genes from plasmid pOH under the control of P_BAD_ promoter is induced by the addition of l-arabinose, allowing the synthesis of Pdu shell proteins and the assembly of Pdu MCPs. The fusion protein PduD_(1–18)_–LacI–EGFP expressed from the plasmid pOH binds to LacI binding sites on the liberated fragment. Plasmid pRW-(*lacO*)_5_ carries five binding sites for the LacI repressor upstream of the *cka* promoter. The binding sites for the LacI repressor and the *cka* promoter are flanked by two target sites for the yeast I-SceI meganuclease. The 594 bp fragment is released from plasmid pRW-(*lacO*)_5_ after *in vivo* restriction by I-SceI. The nucleoprotein complex is then encapsulated in Pdu MCPs during its assembly (shown on the right: the shell proteins are shown as black lines, LacI–EGFP as green circuit, DNA as blue line). The Twin-Strep-tag (light blue) fused to the N-terminus of the major shell protein PduA allows affinity isolation of Pdu MCPs. (**B**) SPR analysis of the PduD_(1–18)_–LacI–EGFP interaction with the chip-immobilized DNA fragment containing LacI operator (DNA sequence is presented above the graph). PduD_(1–18)_–LacI–EGFP was injected over the chip-immobilized LacI operator in 2-fold dilutions ranging from 200 to 1.6 nM for 120 s at a flow rate of 30 μl/min and the dissociation was followed for 80 s. Equilibrium dissociation constant (*K*_D_) was determined by fitting (black lines) the response curves (red lines) to the 1:1 Langmuir interaction model. The average *K*_D_ and standard deviation were determined from two titrations of the protein. (**C**) PduD_(1–18)_–LacI–EGFP fluorescence signal from *E. coli* cells containing pOH is localized at discrete points, demonstrating efficient assembly of microcompartments and encapsulation of the protein PduD_(1–18)_–LacI–EGFP. A representative micrograph is shown. (**D**) LacI–EGFP fluorescence signal from *E. coli* cells containing pOHΔPduD is in most cells localized throughout the cytoplasm.

To package a selected DNA fragment into the Pdu shell, we constructed plasmid pOH, a derivative of pACBSR (24), containing genes for the I-SceI, Gam, and *C. freundii* Pdu shell proteins, as well as for LacI (Figure 1A). These genes were co-expressed from the arabinose-dependent P_BAD_ promoter. We took advantage of the 18 N-terminal amino acids of the PduD enzyme, which can be used to direct proteins into the MCP lumen (5,11,12), and used this targeting signal to label the N-terminal of LacI, which was also fused to EGFP at its C-terminus. Surface plasmon resonance analysis confirmed that purified PduD_(1–18)_–LacI–EGFP stably interacts with operator DNA with an equilibrium dissociation constant of 38 ± 10 nM (Figure 1B). To determine whether this engineered LacI protein can enter the Pdu compartment, we transformed the Keio collection *E. coli* JW0336 strain, which lacks *lacI*, with pOH, and observed the formation of distinct fluorescent foci inside the bacterium after l-arabinose induction (Figure 1C). As a control, we also produced a pOH derivative carrying LacI–EGFP without the targeting PduD_(1–18)_ peptide. In arabinose-treated cells carrying this control pOHΔPduD plasmid, LacI–EGFP was mostly diffuse throughout the cytoplasm (Figure 1D). These results suggest that PduD_(1–18)_–LacI–EGFP is trapped inside the Pdu shell.

To confirm the presence of LacI within the Pdu lumen, we purified the MCPs and analysed them by negative-stain transmission electron microscopy (TEM), western blotting and mass spectrometry (MS). MCPs are typically released from producing bacteria using mild detergents and are then purified in several differential and density gradient centrifugation steps (25). However, it had been reported that a Strep affinity tag on the surface of synthetic microcompartments can facilitate the isolation of MCPs (26). Thus, for our protocol, to purify the compartments, we fused the PduA protein in the shell cluster to a Twin-Strep-tag (see scheme of Pdu in Figure 1A), and we lysed the MCP-producing cells with the bacteria-specific lysis reagent (B-PER™), which has been reported effectively to release empty Pdu compartments from bacteria (27–29).

**Figure 2.**
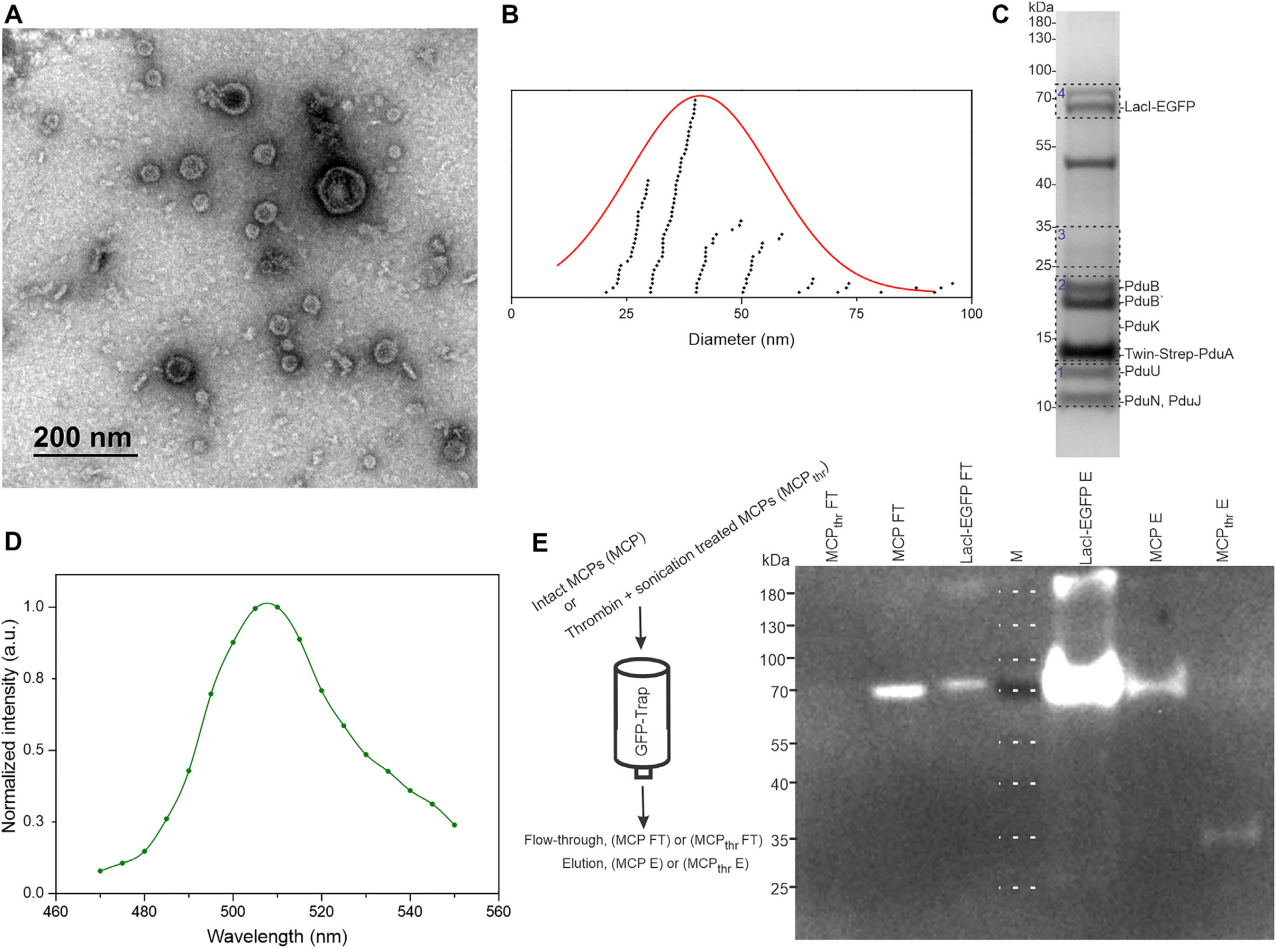
Purification of Pdu-LacI MCPs, immunoprecipitation of PduD_(1–18)_–LacI–EGFP fusion protein and western blotting for EGFP. (**A**) Representative TEM micrograph of Pdu MCPs purified by affinity chromatography from *E. coli* cells. The MCPs have a nearly spherical shape. In addition to MCPs, some impurities can also be seen on the micrograph. (**B**) Size distribution of purified Pdu MCPs, with an average diameter of 41 ± 16 nm. Each dot represents the diameter of a single MCP. The diameters of MCPs were obtained from TEM images. (**C**) SDS-PAGE of Pdu MCPs purified from *E. coli* cells carrying the plasmid pOH by affinity chromatography. Protein bands 1–4, indicated by boxes, were digested with trypsin and analysed by mass spectrometry (Supplementary Table S4). The presence of PduD_(1–18)_–LacI–EGFP and Pdu shell proteins in the purified MCPs was determined. The identity of the major bands is shown on the right. (**D**) The fluorescence emission spectra measured by fluorescence microspectroscopy in purified Pdu MCPs correspond to the emission spectra of EGFP, indicating that the observed fluorescence signal is indeed from PduD_(1–18)_–LacI–EGFP. (**E**) MCPs purified from *E. coli* strain JW0336 carrying plasmid pOH were used to study the localization of PduD_(1–18)_–LacI–EGFP associated with MCPs by western blot. Schematic representation (left) of GFP-Trap column, used to probe GFP accessibility in Pdu MCPs, which were either intact (MCP) or treated with thrombin and sonicated to release the MCP content (MCP_thr_). Flow-through (FT) and elution fractions (E) were analysed for EGFP by Western blot (right). PduD_(1–18)_–LacI–EGFP (67.4 kDa) purified with Ni-chelate affinity was used as a positive control. It is worth noting that purified PduD_(1–18)_–LacI–EGFP carried an N-terminal His tag. Line M shows the PageRuler prestained protein ladder, the protein bands of which are also marked by white dotted lines (Thermo Fisher Scientific, USA).

Although Strep-Tactin magnetic beads are the preferred choice for batch isolation, we were only able to isolate the Twin-Strep-tagged Pdu shells using Strep-TactinXT Sepharose resin in gravity flow. When imaged using TEM, the purified compartments were seen to have a nearly spherical shape and were heterogeneous in size, with an average diameter of 41 ± 16 nm (*n* = 113), with the largest 10% having a diameter of 78 ± 12 nm (Figure 2A and B). Note that variations in sample preparation and loading are known to affect the size of isolated MCPs (5,30). Hence, in another study, the average diameter of purified recombinant *C. freundii* PduA-U microcompartments was reported to be 65 ± 7 nm (10).

To analyse the components of purified Pdu shells, we separated the proteins in the elution fraction by SDS PAGE and identified proteins in selected bands by mass spectrometry (Figure 2C, Supplementary Table S4). The Pdu-shell proteins were found to be enriched. Importantly, we also identified PduD_(1–18)_–LacI–EGFP in a distinct band of ∼70 kDa size, supporting the fluorescence microscopy results (Figure 1C), indicating that LacI–EGFP is packaged and co-purified in the Pdu compartment.

In addition, we used fluorescence microspectroscopy to obtain fluorescence emission spectra of Pdu compartments. We excited the isolated compartments with light from 415 to 455 nm, and the emission signals were collected over a spectral range from 470 to 550 nm. We recorded a typical EGFP spectrum for the compartments with an emission peak of 509 nm (Figure 2D), supporting that the affinity-isolated compartments contain LacI–EGFP.

To investigate whether the PduD_(1–18)_–LacI–EGFP was encapsulated within the Pdu shell, half of the purified MCPs from the strain carrying pOH was treated with thrombin, which cleaves PduA and PduJ, and sonicated to break the Pdu shell. Intact and thrombin-sonication-treated fractions of the MCPs were incubated with GFP-Trap agarose beads to precipitate the PduD_(1–18)_–LacI–EGFP and any associated proteins. Western blots showed that a significant amount of the EGFP fusion protein, failed to be precipitated with GFP-Trap, and could be detected in the flow-through fraction (Figure 2E, Supplementary Figure S1). This indicates that PduD_(1–18)_–LacI–EGFP was present in the lumen of the Pdu shell, but that a portion of the MCPs broke during the GFP trap affinity step. In contrast, PduD_(1–18)_–LacI–EGFP from broken MCPs was found as an ∼35-kDa cleavage product, due to non-specific thrombin activity, only in association with GFP-Trap. These results support the idea that the LacI fusion protein resides in the lumen and is not associated with the outer surface of the Pdu shell. We refer to these protein cages as Pdu-LacI compartments, and, next, we tested their ability to be converted into a synthetic nucleocapsid, containing a DNA fragment.

### Targeting a specific DNA fragment into the lumen of the Pdu-LacI compartment

We previously described the pRW902 plasmid, which allows I-SceI-directed excision of a ∼600 bp DNA segment carrying five LacI repressor binding sites and the colicin K (*cka*) promoter region (24), and we used this here. For simplicity, we refer to plasmid pRW902 here as plasmid pRW-(*lacO*)_5_ (Figure 1A). Hence, we cultured *E. coli* strain JW0336 carrying both plasmids pOH and pRW-(*lacO*)_5_ (Figure 1A) up to mid-logarithmic phase in M9 minimal medium, when the DNA packaging program was triggered by the addition of 0.4% L-arabinose for 1 hour. Bacteria were lysed with B-PER™ and lysozyme in the presence of DNases, RNases, and protease inhibitors, and compartments were affinity-isolated using the Strep-Tactin^®^XT purification system. Prior to subsequent analysis, affinity-isolated compartments were treated (1 h, 37°C) with TURBO^TM^ DNase to degrade any DNA that was not protected within the synthetic Pdu-LacI capsid. This DNase had been shown efficiently remove unprotected DNA copurified with bacterial outer membrane vesicles (OMVs), leaving only internally packaged DNA (31).

To examine the DNA content of the affinity-isolated Pdu-LacI compartments, we hydrolysed the shell with proteinase K and separated the nucleic acids with phenol-chloroform. Before loading onto an agarose gel, RNA was removed from the sample by RNase treatment. The results confirm the association of DNA fragments with the Pdu-LacI compartments, which were 150–300 bp in size (Supplementary Figure S2). These data imply that DNA segments can be packaged within the lumen of the Pdu-LacI compartments since they are protected from TURBO^TM^ DNase treatment.

**Figure 3.**
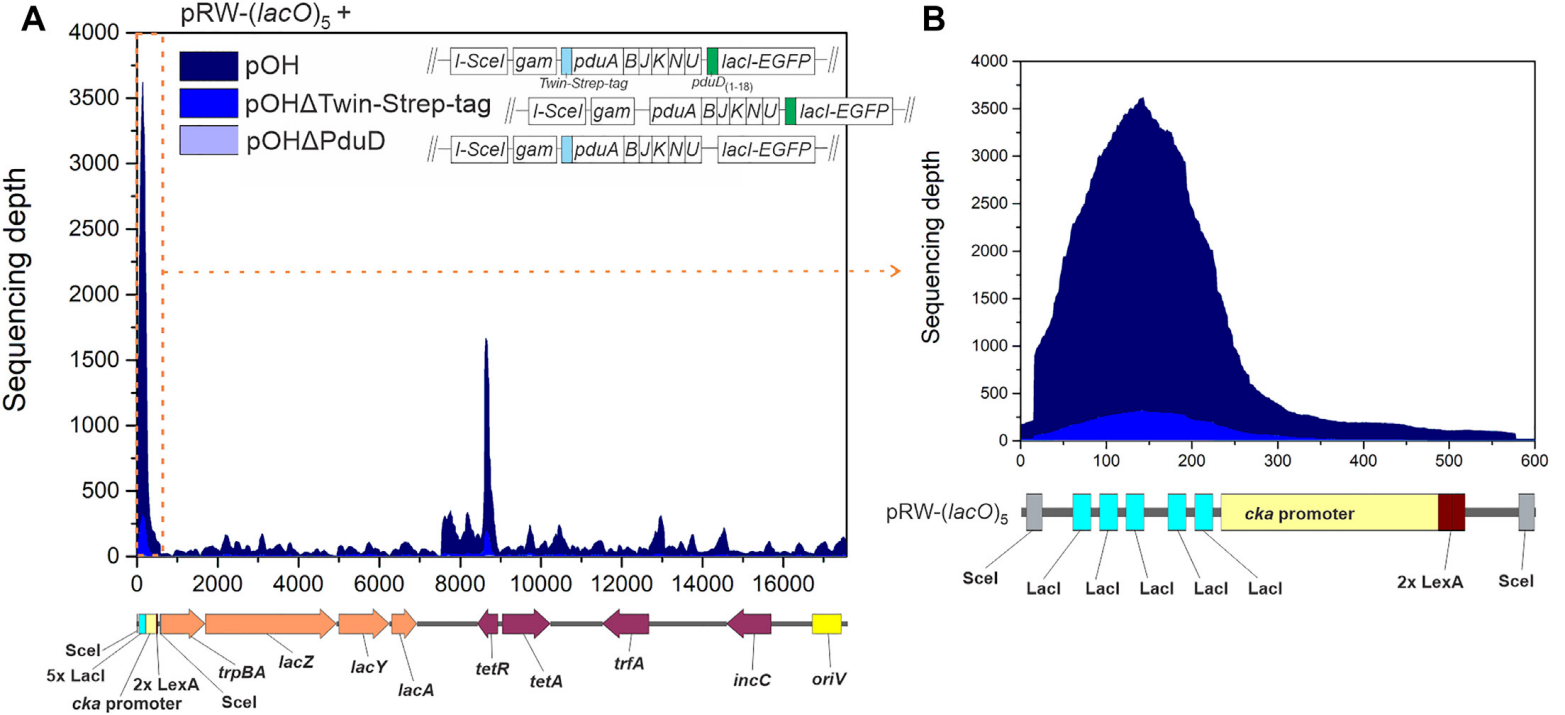
High-throughput sequencing analysis of purified Pdu-LacI MCPs. (**A**) Sequencing depth of plasmid pRW-(*lacO*)_5_ obtained after extraction of the DNA from the affinity purified Pdu-LacI MCPs from the strains carrying: pOH and pRW-(*lacO*)_5_ or pOHΔTwin-Strep-tag and pRW-(*lacO*)_5_ or pOHΔPduD and pRW-(*lacO*)_5_. Extracted DNA was sequenced by Ion Torrent. The sequencing coverage of the plasmids was normalized to the median genome coverage. Two main peaks in read coverage can be observed for plasmid pRW-(*lacO*)_5_. The most pronounced peak in read coverage is for LacI binding sites, while the second highest peak corresponds to the *tetR* gene. (**B**) An inset of the first peak from the main graph (marked by the orange dashed line) is shown on the right. An enrichment of reads with LacI binding sites can be seen. The linearized plasmid pRW-(*lacO*)_5_ map is shown below the graph. Results incorporate sequencing depth values obtained from two biological replicates.

We analysed the extracted DNA by using the Ion Torrent high-throughput sequencing, counting primary alignments. We observed significant read coverage for the pRW-(*lacO*)_5_ plasmid for both biological replicates, with a substantial increase in the coverage of the five LacI repressor binding sites compared to the average read coverage of the entire plasmid (Figure 3, Supplementary Figure S3A). We also detected slightly enriched read coverage for the *tetR* gene, but the rest of the plasmid was only weakly represented. The analysis shows that, of all reads matching the pRW-(*lacO*)_5_ plasmid, for the two replicates, 25% and 33% of the reads are assigned to the LacI-binding DNA segment.

Surprisingly, we obtained a low number of reads for the *cka* promoter region (Figure 3). Since pRW-(*lacO*)_5_ was designed to allow I-SceI-driven release of the segment carrying the five LacI binding sites together with the *cka* promoter region, we expected it to be packaged into a Pdu-LacI compartment, resulting in high sequencing coverage of the region, but our results do not support this. This leads us to speculate that, during the formation of the Pdu compartment, DNases are entrapped in the shell and cleave DNA that is free of associated proteins. Alternatively, nucleases may be localized near the site in the cytoplasm where the compartments are formed. This might be an intrinsic property of bacterial microcompartments that allows Pdu-MCP formation even when their hexagonal and pentagonal tiles assemble around extrachromosomal DNA that would otherwise prevent the closure of the proteinaceous shell.

As a control, we also carried out the l-arabinose-induced Pdu-DNA encapsulation protocol in *E. coli* JW0336 containing plasmids pRW-(*lacO*)_5_ and pOHΔPduD. We observed a low read coverage depth for pRW-(*lacO*)_5_, that reached a maximum of ∼10 reads per position, respectively, whilst the maximum read coverage depth for plasmid pRW-(*lacO*)_5_ in the cells cotransformed with pOH was ∼3600 reads per base pair (Figure 3, Supplementary Figure S3A–D, Table 1). Since the majority of cells carrying pOH, but not cells carrying pOHΔPduD, exhibit distinct fluorescence foci (Figure 1), we conclude that the Pdu_(1–18)_ peptide facilitates LacI-DNA encapsulation and that *lacO* sites in the sequenced samples are enriched *via* PduD_(1–18)_–LacI.

**Table 1.** Percentage of reads aligned to LacI or LacI–LexA operators-containing 600 bp DNA segments, or 600 bp segments from the host *E. coli* JW0336-1 chromosome, or resident plasmids. Columns indicate different plasmids in *E. coli* strain JW0336-1. Rows show the percentage of reads aligned to random 600 bp segments from the chromosome, pACBSR derivatives (pOH), pRW derivatives, or to the cloned 600 bp segment containing the LacI operator sites (*lacO*) or the LacI and LexA sites (pRW-LexA). The percentage of reads per 600 bp segment is calculated with respect to all reads in the sample of the selected strain. Results are averages from two biological replicates shown in Supplementary Table S5. For the sample pRW-LexA pOH the results from one experiment are shown

*E. coli* JW0336-1	pRW-(*lacO*)_5_pOH	pRW-(*lacO*)_5_pOHΔPduD	pRW-(*lacO*)_5_pOHΔTwin-Strep-tag	pRW-(*lacO*)_8_pOH	pRW-(*lacO*)_8_pOHΔLacI	pRW-LexA pOH	pRW-LexA pOHΔLacI
	Average % of reads aligned to 600 bp segment						
Chromosome	0.006	0.011	0.009	0.008	0.010	0.006	0.009
pOH	2.614	0.977	1.820	1.387	1.555	3.148	2.101
pRW	0.362	0.005	0.078	0.691	0.132	0.018	0.023
600 bp (*lacO*)_4–8_	2.959	0.014	0.507	10.141	0.070	0.143	0.005

To check that DNA fragments that are co-purified with the Pdu-LacI compartments are not simply an artefact of the affinity purification protocol, for example due to nonspecific binding of the LacI-DNA complex to the affinity resin, we also performed the protocol with *E. coli* JW0336 carrying pRW-(*lacO*)_5_ and pOHΔTwin-Strep-tag. The latter plasmid carries the *pduA* gene, which is not tagged with the Twin-Strep sequence, and therefore the resulting Pdu-LacI compartments do not allow affinity purification. Analysis of the resulting material revealed ∼12-fold lower coverage of DNA sequence reads mapped to the five LacI target sites, compared to the strain carrying the pOH and pRW-(*lacO*)_5_ plasmids, considering the average of two biological replicates (Supplementary Figure S3E and F). Note that, in the control strain, the Pdu-LacI compartments were formed (Supplementary Figure S4).

### DNA protected from nucleases corresponds to LacI binding sites

**Figure 4.**
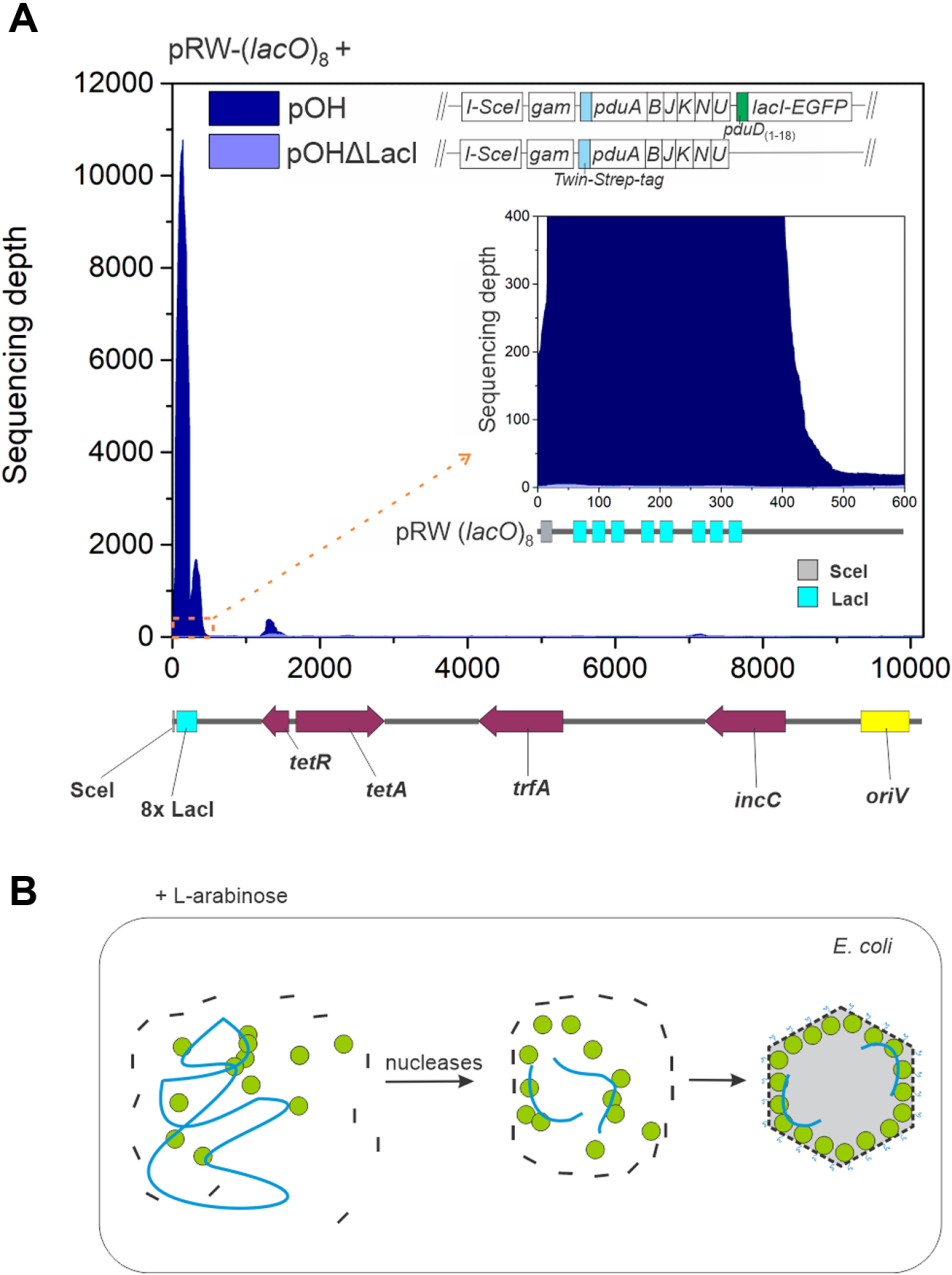
Modifications of the LacI binding site region affect the region of protected DNA. (**A**) Sequencing depth of plasmid pRW-(*lacO*)_8_ obtained after extraction of the DNA from the affinity purified Pdu-LacI MCPs from the strains carrying: (i) pOH and pRW-(*lacO*)_8_ or (ii) pOHΔLacI and pRW-(*lacO*)_8_. Extracted DNA was sequenced by Ion Torrent. The sequencing coverage of the plasmids was normalized to the median genome coverage. The highest peak of read coverage is observed for the region containing LacI binding sites. The inset of the major peak is shown on the right, detailing the LacI operator region. Linearized plasmid pRW-(*lacO*)_8_ map is shown below the graph. Results show sequencing depth obtained from a single experiment, and biological replicates are shown in Supplementary Figure S3. (**B**) Schematic representation of the proposed extrachromosomal DNA degradation by host DNases during Pdu-LacI shell assembly. The shell proteins are shown as black lines, the plasmid DNA as blue line, LacI–EGFP as green circuit.

To confirm that the regions of high read coverage on pRW-(*lacO*)_5_ correspond to DNA sites protected from nucleases by LacI, we prepared two derivatives. We designed the pRW-(*lacO*)_8_ plasmid in which we replaced the *cka* promoter region with three additional LacI operators (Figure 4A). In addition, the pRW-(*lacO*)_8_ carried only a single I-SceI recognition sequence, so we could check whether the DNA segment containing LacI operators must be excised, or whether a segment of the linearized plasmid could also be packaged into the Pdu shell. To facilitate the transformation of pRW-(*lacO*)_8_, we also removed ∼7 kb DNA from pRW-(*lacO*)_5_.

After performing the protocol with the strain carrying the plasmids pOH and pRW-(*lacO*)_8_, we found that the majority of the sequence reads matching plasmid pRW-(*lacO*)_8_ (44% and 86% for the two replicates, respectively) mapped to the LacI operator region and that the high sequencing coverage included all eight LacI operator sites (Figure 4, Supplementary Figure S3G). On a genome-wide level, reads that could be assigned to the LacI operator region on pRW-(*lacO*)_8_ accounted for more than 10% of all reads in a sample (Table 1). Since the pRW-(*lacO*)_8_ plasmid contains a single I-SceI site, this indicates that release of the fragment from the plasmid is not required for packaging into the Pdu-LacI MCP. This supports the idea that host nucleases promote Pdu shell formation by removing extrachromosomal DNA (Figure 4B).

As a further control, we examined DNA loading into the Pdu shell in the strain carrying pRW-(*lacO*)_8_ and a pOH derivative without the PduD_(1–18)_-tagged LacI–EGFP (pOHΔLacI). We found negligible sequencing depth for all regions, showing that LacI operator DNA is not protected from nucleases in the absence of LacI (Figure 4). However, sequencing revealed a characteristic pattern of read coverage for the pOH, pOHΔPduD, pOHΔTwin-Strep-tag and pOHΔLacI plasmids, irrespective of which derivative was used (Supplementary Figure S3). In addition, some sequence reads from certain parts of the host chromosome repeatedly appeared (Supplementary Figure S5). We speculate that, because of the spatial organization of transcription and translation in bacteria, some DNA, localized in the vicinity of Pdu shell assembly, may be packaged in a LacI-independent manner, and this is not due to a specific complex, as we find with LacI-protected fragments targeted to Pdu shells. An example of this is a number of reads that map to pACBSR derivatives encoding Pdu components (Table 1). It is worth noting that the pRW derivatives carry a low copy number RK2 replicon and the pACBSR derivatives carry a p15A replicon with approximately 2 and 9 copies per cell, respectively (32), which likely contributes to the high sequence coverage observed for pOH and its derivatives.

### LacI-driven encapsulation of operators for a second transcription factor

To show that the DNA segment encapsulated in Pdu can carry target sites for another transcription factor, in addition to LacI operators, we modified pRW-(*lacO*)_5_ to give derivatives, pRW-LexA and pRW-LexA-mCherry. In both these derivatives, two DNA sites for I-SceI flank a DNA segment carrying two LacI-binding sites which flank the duplicated LexA operator from the *recA* gene of *Bacillus thuringiensis* (hence (*lacO*)_2_-(*lexA*_BT_)_2_-(*lacO*)_2_). Plasmid pRW-LexA also encodes a mutant form of the *B. thuringiensis* LexA repressor that is incapable of self-cleavage (LexA_BT_) because alanine 96 is replaced by aspartic acid. Similarly, plasmid pRW-LexA-mCherry encodes the LexA_BT_ fused to mCherry. Expression of the gene for LexA_BT_ or LexA_BT_-mCherry in these plasmids was under the control of the *lexA* promoter of *E. coli*: note that LexA of *E. coli* does not recognize the LexA operators of *B. thuringiensis* (33).

**Figure 5.**
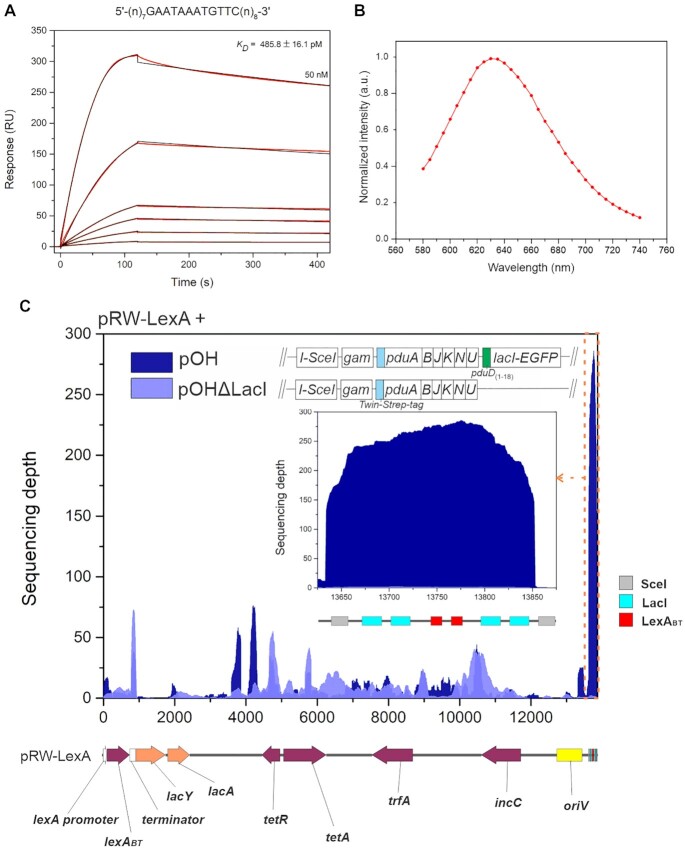
LacI-directed encapsulation of the DNA fragment carrying the target DNA sites for *B. thuringiensis* LexA repressor. (**A**) SPR analysis of LexA_BT_–mCherry interaction with the DNA fragment containing the LexA operator immobilized on the chip (DNA sequence is presented above the graph). Proteins were injected over the chip-immobilized LexA operator in 2-fold dilutions of 50 to 1.6 nM for 120 s at a flow rate of 30 μl/min, and dissociation was monitored for 300 s. Sensorgrams (red lines) are shown along with the apparent equilibrium dissociation constant (*K*_D_), which was determined from the response curves (red lines) as a function of LexA_BT_–mCherry concentration injected over the DNA. The equilibrium dissociation constant (*K*_D_) was determined by fitting (black lines) the response curves to the 1:1 Langmuir interaction model. The average *K*_D_ and standard deviation were determined from two titrations of the proteins. (**B**) Fluorescence emission spectra measured by fluorescence microspectroscopy in purified Pdu MCPs correspond to the emission spectra of mCherry, indicating that the observed fluorescence signal is from LexA_BT_-mCherry. (**C**) Sequencing depth of plasmid pRW-LexA obtained after extraction of the DNA from the affinity purified Pdu-LacI MCPs from the strains carrying pRW-LexA and either pOH or pOHΔLacI. Extracted DNA was sequenced by Ion Torrent. The sequencing coverage of the plasmids was normalized to the median genome coverage. The highest peak in read coverage is observed for the region containing four LacI and two LexA binding sites (marked by the orange dashed line and shown as inset). The results from one experiment are shown. Linearized plasmid pRW-LexA map is shown below the graph.

Using SPR, we first confirmed that the LexA_BT_-mCherry protein retains DNA-binding properties and interacts, with high affinity, with the operator used in pRW-LexA-mCherry (Figure 5A). Next, in *E. coli* strain JW0336, carrying plasmids pOH-S and pRW-LexA-mCherry, we induced LexA_BT_-mCherry synthesis with a subinhibitory concentration of nalidixic acid, and initiated the Pdu-DNA (*lacO*)_2_-(*lexA*_BT_)_2_-(*lacO*)_2_ encapsulation protocol with l-arabinose. Note that, in pOH-S, a stop codon is inserted between the *lacI* and *EGFP* genes, so that arabinose-induced PduD_(1–18)_–LacI lacks the fluorescent label. We used fluorescence microscopy and illuminated the purified compartments with light from 532 to 557 nm to excite the mCherry fused to LexA_BT_ (Figure 5B and Supplementary Figure S6). The recorded spectra confirm the presence of LexA_BT_–mCherry in the capsids with the characteristic emission peak at ∼630 nm for the red-shifted-mCherry (34).

To analyse whether the (*lacO*)_2_-(*lexA*_BT_)_2_-(*lacO*)_2_ DNA segment was present in the affinity-purified Pdu capsids, we sequenced DNA from Pdu compartments purified from the strain carrying plasmids pRW-LexA and pOH, or from the control strain lacking a gene for PduD_(1–18)_–LacI. For the PduD_(1–18)_–LacI producing strain, most sequencing reads, 32%, assigned to the pRW-LexA plasmid mapped to the region between the two I-SceI target sites (Figure 5C). Since almost no reads for this region were detected in the compartments purified from the PduD_(1–18)_–LacI-deficient strain (Figure 5C, Table 1), the data show that DNA segments with target sites for more than one transcription factor could be selectively packaged into MCPs *via* PduD_(1–18)_–LacI.

In conclusion, to our knowledge, this is the first report of affinity purification of Pdu shells containing specified DNA segments. We conclude that MCPs can enable LacI-directed packaging of multiple target sites for at least one transcription factor. Surprisingly cis-attached sequences are not purified and we hypothesise that host nucleases may play a role in MCP assembly (Figure 4B). Previous efforts to encapsulate nucleic acids *in vivo* into the nonviral protein capsid have focused on packaging RNA *via* complementary electrostatic interactions between the RNA and the interior of engineered protein shells (17,18,35). Such nucleocapsids can be used as delivery containers for various RNA molecules or RNA-protein complexes (17,36). In comparison, our system enables packaging of DNA segments into the Pdu shell *via* specific protein-DNA interactions. Since DNA can serve as a scaffold to arrange chimeric enzymes of a specific metabolic pathway into a functional metabolic structure (20), we anticipate, that by using a DNA carrying multiple target site sequences and enzymes linked to specific transcription factors, a scaffold can be provided to direct selected enzymes within the MCP. Such DNA-directed assembly of nonendogenous biosynthetic pathways within MCPs can increase the metabolic product yield and sequester toxic metabolic intermediates in the MCP lumen (14).

## DATA AVAILABILITY

All data are available from the corresponding author upon request. The nucleotide sequences of the pOH, pOHΔTwin-Strep-tag, pOHΔPduD, pOHΔLacI, pRW-(*lacO*)_5_, pRW-(*lacO*)_8_, pRW-LexA and pRW-LexA-mCherry can be found in Supplementary Table S3. The sequence data were deposited under the Bioproject with accession number PRJNA693827, BioSample SAMN17478165 (SRX16679928, SRX16679927, SRX16679925, SRX16679922, SRX9933758, SRX9933757, SRX9933756, SRX9933755, SRX9933754, SRX9933753, SRX9933752). The mass spectrometry proteomics data have been deposited to the ProteomeXchange Consortium via the PRIDE partner repository with the dataset identifier PXD035877.

## Supplementary Material

gkac714_Supplemental_Files
