# Supplementary material for: A method for targeting a specified segment of DNA to a bacterial microorganelle
Source: Nucleic Acids Res. 2022 Aug 27;50(19):e113. doi: 10.1093/nar/gkac714 (PMC9638918; doi:10.1093/nar/gkac714)
Supplement: gkac714_Supplemental_Files [file gkac714_supplemental_files.zip › Otonicar et al Supplementary_data_28-07-22_R3.pdf]

Supplementary Online Information for:

A method for targeting a specified segment of DNA to a bacterial microorganelle

Jan Otoničar, Maja Hostnik, Maja Grundner, Rok Kostanjšek, Tajda Gredar, Maja Garvas, Zoran Arsov, Zdravko Podlesek, Cene Gostinčar, Jernej Jakše, Stephen J. W. Busby and Matej Butala

Supplementary Methods (Plasmid construction)

Supplementary Tables S1-S5

Supplementary Figures S1-S6

Supplementary Methods

Plasmid construction

The list of primers, bacterial strains and plasmids used is shown in Supplementary Tables S1-S3. All the restriction enzymes used were from Thermo Fisher Scientific, USA. pOH was constructed by cloning *pduABJKNU* (generous gift of Dr. M. Warren), *Twin-Strep-tag,* and *pduD*$_{(1\text{-}18)}$*-lacI-EGFP* (both provided by Twist Bioscience, USA) into the vector pACBSR-DL1. First, the Gam protein gene was amplified from pACBSR by PCR (1). The SphI and NotI restriction sites were introduced into the PCR product of the gene *via* primers GamUP_SphI and GamDO_NotI. The gene for Twin-Strep-tag was amplified by PCR using the 5' primer StrepUP_NotI to introduce the NotI restriction site and the 3' primer StrepDO_XhoI to introduce the XhoI restriction site. The genes for the shell proteins (*pduABJKNU*) were amplified by PCR with primers PduUP_XhoI and PduDO_BglII_SphI from the pLysS-*pduABB'JKNU* (2). The amplified *gam* gene was then cut with SphI and NotI, the amplified fragment *Twin-Strep-tag* was cut with NotI and XhoI, and the amplified *pdu* operon was cut with SphI and XhoI. All three digested fragments were first purified and then ligated together into the vector pACBSR-DL1 previously digested with SphI. Gene for PduD$_{(1\text{-}18)}$-LacI-EGFP fusion protein was amplified using primers LacIPduDUP_BglII and LacIDo_BglII, cut with BglII and ligated into the final version of the BglII-digested pACBSR vector to obtain the final vector pOH.

The plasmid pOHΔTwin-Strep-tag was constructed by cloning *pduABJKNU* and *pduD*$_{(1\text{-}18)}$*-lacI-EGFP* into the vector pACBSR-DL1. As for pOH, the gene for Gam was first amplified by PCR. The restriction sites NotI and SphI were introduced into the PCR product of the gene via the 3' and 5' primers. The genes for the shell proteins (*pduABJKNU*) were amplified by PCR using the primers PduUP_NST_NotI and PduDO_BglII_SphI. The amplified *gam* gene was then cut with SphI and NotI, and the amplified *pdu* operon was cut with NotI and SphI. All three digested fragments were first purified and then ligated together into the vector pACBSR-DL1, which had been previously digested with SphI. Gene for PduD$_{(1\text{-}18)}$-LacI-EGFP fusion protein was amplified using primers LacIPduDUP_BglII and LacIDo_BglII, cut with BglII and ligated into the final version of pACBSR vector previously digested with BglII to obtain the final vector pOHΔTwin-Strep-tag.

The plasmid pOHΔPduD was constructed by first PCR amplifying the pOH plasmid using Phusion high-fidelity DNA polymerase (Thermo Fisher Scientific, USA) and primers RBS_LacI_F and PduU_R. The purified DNA fragment was ligated to obtain vector pOHΔPduD.

The plasmid pOH-S was constructed by first PCR amplifying the LacI gene using pOH plasmid as a matrix and Phusion high-fidelity DNA polymerase (Thermo Fisher Scientific, USA) and primers LacI_STOP_F and LacI_STOP_R. The purified DNA fragment was digested with SmaI and subsequently ligated to obtain vector pOH-S.

The pRW-(*lacO*)$_8$ plasmid was designed by first truncating vector pRW-(*lacO*)$_5$ and then cloning the fragment carrying 3 *lacI* operators (provided by Twist Bioscience, USA) into the shortened version of vector pRW-(*lacO*)$_5$. Vector pRW-(*lacO*)$_5$ was first digested with BstBI and EcoNI enzymes. Afterwards, blunting reaction was performed. To circulize the linearized vector, ligation reaction was performed with purified product of restriction and blunting reaction. To construct vector pRW-(*lacO*)$_8$, the fragment with 3 *lacO* sites and the short version of vector pRW-(*lacO*)$_5$ were digested with EcoRI and HindIII enzymes. The digested fragment and vector were ligated to obtain the vector pRW-(*lacO*)$_8$.

To obtain a DNA fragment (*lacO*)$_2$-(*lexA*$_{BT}$)$_2$-(*lacO*)$_2$ equal molar concentrations of primer pairs NdeI_SceI:BglII_SceI, NdeI_LexA:KpnI_LexA, BamHI_SceI_LacI:KpnI_LacI_SceI were each mixed in 1x PBS pH 7.4 and annealed over a temperature gradient from 95 °C to 30 °C. All three fragments were purified and then ligated all together into vector pJET. Subsequently, the (*lacO*)$_2$-(*lexA*$_{BT}$)$_2$-(*lacO*)$_2$ fragment was amplified by PCR using primers 2LacI_2LexA_2LacI_F and 2LacI_2LexA_2LacI_R. The purified fragment and vector pRW50 were digested with BamHI and BglII and ligated together to yield a new pRW derivative. DNA fragments carrying either the gene for *lexA* or *lexA-mCherry* (provided by Twist Bioscience, USA) under the control of the *E. coli lexA* promoter were then cut with AflII and HindIII and ligated into the identically cut pRW vector, to obtain the plasmids pRW-LexA or pRW-LexA-mCherry.

For surface plasmon resonance (SPR) analysis, to purify the PduD$_{(1-18)}$-LacI-EGFP or LexA$_{BT}$-mCherry fusion proteins both carrying a hexa-histidine tag appended to the C-terminus, genes for these fusion proteins (provided by Twist Bioscience, USA) were cloned separately into the expression vector pET21c. The gene for PduD$_{(1-18)}$-lacI-EGFP was amplified by PCR using the primers PduD_DO and EGFP_UP. The gene for LexA$_{BT}$-mCherry was amplified by PCR using the primers LexA_NdeI and LexA_XhoI. The vector and gene were digested with NdeI and XhoI. The digested and purified fragment and vector were then ligated to obtain vector pET21c-lacI or pET21c-lexA. All constructs were sequenced by Microsynth AG. Annotated sequences of pOH, pRW-(*lacO*)$_5$ or its derivatives are shown in Supplementary Table S3.

Supplementary Table S1. Oligonucleotide primers used in this study. 'Bio' indicates the biotin labelling and [Phos] indicates that the primers were phosphorylated at the 5`- site.

Primer	Sequence (5'-3')
GamUP_SphI	CAGGCATGCTAAGGAGGTTATAAAAAATGG
GamDO_NotI	GCGGCGGCCGCTTATACCTCTGAATCAATATCAACCTG
StrepUP_NotI	GCGGCGGCCGCAATAATTTTGTTTAACTTTAAGAAGGAGATATACAT ATGTGGTCGCACCCGCAGTTCGAG
StrepDO_XhoI	CGCCTCGAGGCTTTTTCGAACTGCGGGTG
PduUP_XhoI	CGCCTCGAGCCAACAAGAAGCGTTAGGAATGGTAG
PduDO_BglII_SphI	CAGGCATGCATGGATGGAGATCTTTATGTCCGGGTGATGGGACAG GC
LacIPduDUP_BglII	GCGAGATCTTTCTCCATACCCGTTTTTTTGGGCTAGCAGGAGGGTA CCTATATGGAAATCAATGAAAAGCTGCTGCGCCAGATTATTGAAGA CGTACTGTCTGAAAAACCAGTAACGTTATACGATGTCG
LacIDo_BglII	GCGAGATCTTTACTTGTACAGCTCGTCCATGCC
PduUP_NST_NotI	GCGGCGGCCGCAATAATTTTGTTTAACTTTAAGAAGGAGATATACAT ATGCAACAAGAAGCGTTAGGAATGGTAG
RBS_LacI_F	[Phos]ACTAGAAATAATTTTGTTTAACTTTAAGAAGGAGATATACATAT GAAACCAGTAACGTTATACGATGTCG
PduU_R	[Phos]AGATCTTTATGTCCGGGTGATGGGA
LacI_STOP_F	GAAAGCGGGCAGTAACCCGGGAGCAAG
LacI_STOP_R	CTTGCTCCCGGGTTACTGCCCGCTTTC
NdeI_SceI	[Phos]TATGCTCCGTCTTTAATTGTTATCCGCTCACAATTTCTGACTT TTAATTGTTATCCGCTCACAATTGTGCTGGCTCAATTCATTACCCTG TTATCCCTACAATTCAGATCT
BglII_SceI	AGATCTGAATTGTAGGGATAACAGGGTAATGAATTGAGCCAGCACA ATTGTGAGCGGATAACAATTAAAAGTCAGAAATTGTGAGCGGATAA CAATTAAAGACGGAGCA
NdeI_LexA	[Phos]TATGCAAATCGAATAAATGTTCGATTTAAATCGAATAAATGTT CGATTTTATGGTAC
KpnI_LexA	[Phos]CATAAAATCGAACATTTATTCGATTTAAATCGAACATTTATTCG ATTTGCA
BamHI_SceI_LacI	GGATCCCGAAGTATTACCCTGTTATCCCTAGAATTCCCAGAATTGTT ATCCGCTCACAATTCTGTCTTGCTAATTGTTATCCGCTCACAATTTG CGCGGGTAC
KpnI_LacI_SceI	[Phos]CCGCGCAAATTGTGAGCGGATAACAATTAGCAAGACAGAATT GTGAGOGGATAACAATTCTGGGAATTCTAGGGATAACAGGGTAATA CTTCGGGATCC
2LacI_2LexA_2LacI_F	AGATCTGAATTGTAGGGATAACAGGGTAATGAATTGAGCC
2LacI_2LexA_2LacI_R	GGATCCCGAAGTATTACCCTGTTATCCCTAGAATTCCCA
PduD_DO	CGCCATATGATGGAAATCAATGAAAAGCTGCTGCGCC
EGFP_UP	CGCCTCGAGGGATCCACGCGGAACCAGCTTGTACAGCTCGTCCAT GCCG
LexA_NdeI	CGCCATATGTTAGAAAACATGGAAAAGTTAACGAAACGCC
LexA_XhoI	CGCCTCGAGGGATCCACGCGGAACCAGCTTGTACAGCTCGTCCAT GCC
LacI_u	GTTACTACTCGAGTCAGAAATTGTGAGCGGATAACAATTAAAGACG
LacI_d	CGTCTTTAATTGTTATCCGCTCACAATTTCTGACT
LexA_u	GTTACTACTCGAGCGAAAAATCGAATAAATGTTCGATTTTTG
LexA_d	CAAAAATCGAACATTTATTCGATTTTT

S1	CGCTCGAGTAGTAAC-Bio

Supplementary Table S2. Bacterial strains used in this study.

Strains	Genotype and/or phenotype	Reference
DH5α	F$^-$ *endA1 glnV44 thi1 recA1 relA1 gyrA96 deoR nupG purB20* φ80d*lacZ*ΔM15 Δ(*lacZYA-argF*)U169, hsdR17($r_K^- m_K^+$), λ$^-$	Thermo Fisher Scientific
BL21(DE3)	F$^-$ *ompT gal dcm lon hsdS*$_B$($r_B^- m_B^-$) λ(DE3 [*lacI lacUV5-T7p07 ind1 sam7 nin5*]) [*malB*$^+$]$_{K-12}$(λ^S)	Invitrogen
JW0336-1	F- *Δ(araD-araB)567 ΔlacZ4787*(::rrnB-3) *ΔlacI785::kan λ*$^-$ *rph-1 Δ(rhaD-rhaB)568 hsdR514*	(3)

Supplementary Table S3. Plasmids used in this study.

Plasmid name	Genotype	Description of contents/construction	Reference
pOH	pACBSR *I-SceI gam pduA-Twin-Strep-tag pduBJKNU* $pduD_{(1-18)}$*-lacI-EGFP*	Expression of I-SceI, Gam, Pdu shell proteins with PduA strep tagged and $PduD_{(1-18)}$-LacI-EGFP fusion protein under arabinose promoter, Cm^R	This study
pACBSR-DL1	pACBSR *I-SceI gam Twin-Strep-tag-lacI-EGFP*	Expression of I-SceI, *gam, lacI* fusion protein under arabinose promoter, Cm^R	(4)
pOHΔTwin-Strep-tag	pACBSR *I-SceI gam pduABJKNU* $pduD_{(1-18)}$*-lacI-EGFP*	Expression of I-SceI, Gam, Pdu shell proteins with PduA non-tagged and $PduD_{(1-18)}$-LacI-EGFP fusion protein under arabinose promoter, Cm^R	This study
pOHΔPduD	pACBSR *I-SceI gam pduABJKNU lacI-EGFP*	Expression of I-SceI, Gam, Pdu shell proteins with PduA strep tagged and LacI-EGFP fusion protein under arabinose promoter, Cm^R	This study
pOH-S	pACBSR *I-SceI gam pduABJKNU* $pduD_{(1-18)}$*-lacI*	Expression of I-SceI, Gam, Pdu shell proteins with PduA strep tagged and $PduD_{(1-18)}$-LacI fusion protein under arabinose promoter, Cm^R	This study
pRW902 here named as pRW-$(lacO)_5$	pRW902	Vector carrying 5 LacI operators and *cka* promoter flanked by 2 target sites for the yeast meganuclease I-SceI, Tc^R	(4)
pET21c-lacI	pET21c $pduD_{(1-18)}$*-lacI-EGFP-His-tag*	Over-expression of C-terminal His-tag $PduD_{(1-18)}$-LacI-EGFP fusion protein under T7 promoter, Amp^R	This study
pET21c-lexA	pET21c $LexA_{BT}$*-mCherry-His-tag*	Over-expression of C-terminal His-tag $LexA_{BT}$-mCherry fusion protein under T7 promoter, Amp^R	This study
pRW-$(lacO)_8$	pRW-$(lacO)_8$	Vector carrying 8 LacI operators flanked by single target site for the yeast meganuclease I-SceI, Tc^R	This study
pRW-LexA	pRW-$(lacO)_2$-$(lexA_{BT})_2$-$(lacO)_2$-*lexA*	Vector carrying a fragment with 2 LacI operators upstream and downstream of 2 LexA operators from *Bacillus thuringiensis*, flanked by 2 target sites for yeast meganuclease I-SceI, and LexA protein under the *lexA* promoter of *E. coli*, Tc^R	This study
pRW-LexA-mCherry	pRW-$(lacO)_2$-$(lexA_{BT})_2$-$(lacO)_2$ -*lexA-mCherry*	A pRW-LexA plasmid derivative carrying a gene for the LexA-mCherry fusion protein under the control of *E. coli lexA* promoter, Tc^R	This study
pLysS-pduABJKNU	pLysS *pduABJKNU*	Vector used for initial amplification of *pdu* genes, Cm^R	(2)
pJET1.2	pJET1.2	Vector used for cloning separate fragments, Amp^R	Thermo Fisher Scientific, USA

>Complete sequence of the pOH plasmid. Features of the plasmid are shown in different colours: shown in orange, blue and green are genes *I-SceI, gam, Twin-Strep-tag – pduA, pduBJKNU, pduD$_{(1\text{-}18)}$-lacI-EGFP* and *araBAD* promoter. Target sites for SphI, XhoI, SmaI and BglII restriction enzymes are shown in red.

GTGCACTCTCAGTACAATCTGCTCTGATGCCGCATAGTTAAGCCAGTATACACTCCGCTATCGCTACGTGACTG
GGTCATGGCTGCGCCCCGACACCCGCCAACACCCGCTGACGCGCCCTGACGGGCTTGTCTGCTCCCGGCATCC
GCTTACAGACAAGCTGTGACCGTCTCCGGGAGCTGCATGTGTCAGAGGTTTTCACCGTCATCACCGAAACGCG
CGAGGCAGCAAGGAGATGGCGCCCAACAGTCCCCCGGCCACGGGGCCTGCCACCATACCCACGCCGAAACAA
GCGCTCATGAGCCCGAAGTGGCGAGCCCGATCTTCCCCATCGGTGATGTCGGCGATATAGGCGCCAGCAACC
GCACCTGTGGCGCCGGTGATGCCGGCCACGATGCGTCCGGCGTAGAGGATCTGCTCATGTTTGACAGCTTATC
ATCGATGCATAATGTGCCTGTCAAATGGACGAAGCAGGGATTCTGCAAACCCTATGCTACTCCGTCAAGCCGT
CAATTGTCTGATTCGTTACCAATTATGACAACTTGACGGCTACATCATTCACTTTTTCTTCACAACCGGCACGGA
ACTCGCTCGGGCTGGCCCCGGTGCATTTTTTAAATACCCGCGAGAAATAGAGTTGATCGTCAAAACCAACATTG
CGACCGACGGTGGCGATAGGCATCCGGGTGGTGCTCAAAAGCAGCTTCGCCTGGCTGATACGTTGGTCCTCGC
GCCAGCTTAAGACGCTAATCCCTAACTGCTGGCGGAAAAGATGTGACAGACGCGACGGCGACAAGCAAACAT
GCTGTGCGACGCTGGCGATATCAAAATTGCTGTCTGCCAGGTGATCGCTGATGTACTGACAAGCCTCGCGTAC
CCGATTATCCATCGGTGGATGGAGCGACTCGTTAATCGCTTCCATGCGCCGCAGTAACAATTGCTCAAGCAGAT
TTATCGCCAGCAGCTCCGAATAGCGCCCTTCCCCTTGCCCGGCGTTAATGATTTGCCCAAACAGGTCGCTGAAA
TGCGGCTGGTGCGCTTCATCCGGGCGAAAGAACCCCGTATTGGCAAATATTGACGGCCAGTTAAGCCATTCAT
GCCAGTAGGCGCGCGGACGAAAGTAAACCCACTGGTGATACCATTCGCGAGCCTCCGGATGACGACCGTAGT
GATGAATCTCTCCTGGCGGGAACAGCAAAATATCACCCGGTCGGCAAACAAATTCTCGTCCCTGATTTTTCACC
ACCCCCTGACCGCGAATGGTGAGATTGAGAATATAACCTTTCATTCCCAGCGGTCGGTCGATAAAAAAATCGA
GATAACCGTTGGCCTCAATCGGCGTTAAACCCGCCACCAGATGGGCATTAAACGAGTATCCCGGCAGCAGGG
GATCATTTTGCGCTTCAGCCATACTTTTCATACTCCCGCCATTCAGAGAAGAAACCAATTGTCCATATTGCATCA
GACATTGCCGTCACTGCGTCTTTTACTGGCTCTTCTCGCTAACCAAACCGGTAACCCCGCTTATTAAAAGCATTC
TGTAACAAAGCGGGACCAAAGCCATGACAAAAACGCGTAACAAAAGTGTCTATAATCACGGCAGAAAAGTCC

araBAD promoter

ACATTGATTATTTGCACGGCGTCACACTTTGCTATGCCATAGCATTTTTATCCATAAGATTAGCGGATCCTACCT

GACGCTTTTTATCGCAACTCTCTACTGTTTCTCCATACCCGTTTTTTTGGGCTAGCAGGAGGGTACCTATATGCA

I-SceI

TATGAAAAACATCAAAAAAAACCAGGTAATGAACCTGGGTCCGAACTCTAAACTGCTGAAAGAATACAAATCC
CAGCTGATCGAACTGAACATCGAACAGTTCGAAGCAGGTATCGGTCTGATCCTGGGTGATGCTTACATCCGTT
CTCGTGATGAAGGTAAAACCTACTGTATGCAGTTCGAGTGGAAAAACAAAGCATACATGGACCACGTATGTCT
GCTGTACGATCAGTGGGTACTGTCCCCGCCGCACAAAAAAGAACGTGTTAACCACCTGGGTAACCTGGTAATC
ACCTGGGGCGCCCAGACTTTCAAACACCAAGCTTTCAACAAACTGGCTAACCTGTTCATCGTTAACAACAAAAA
AACCATCCCGAACAACCTGGTTGAAAACTACCTGACCCCGATGTCTCTGGCATACTGGTTCATGGATGATGGTG
GTAAATGGGATTACAACAAAAACTCTACCAACAAATCGATCGTACTGAACACCCAGTCTTTCACTTTCGAAGAA
GTAGAATACCTGGTTAAGGGTCTGCGTAACAAATTCCAACTGAACTGTTACGTAAAAATCAACAAAAACAAAC
CGATCATCTACATCGATTCTATGTCTTACCTGATCTTCTACAACCTGATCAAACCGTACCTGATCCCGCAGATGA

SphI

TGTACAAACTGCCGAACACTATCTCCTCCGAAACTTTCCTGAAATAAGGGCCCGACGTCTGCAGGCATGCTAAG

gam

GAGGTTATAAAAAATGGATATTAATACTGAAACTGAGATCAAGCAAAAGCATTCACTAACCCCCTTTCCTGTTT
TCCTAATCAGCCCGGCATTTCGCGGGCGATATTTTCACAGCTATTTCAGGAGTTCAGCCATGAACGCTTATTAC

ATTCAGGATCGTCTTGAGGCTCAGAGCTGGGCGCGTCACTACCAGCAGCTCGCCCGTGAAGAGAAAGAGGCA
GAACTGGCAGACGACATGGAAAAAGGCCTGCCCCAGCACCTGTTTGAATCGCTATGCATCGATCATTTGCAAC
GCCACGGGGCCAGCAAAAAATCCATTACCCGTGCGTTTGATGACGATGTTGAGTTTCAGGAGCGCATGGCAG
AACACATCCGGTACATGGTTGAAACCATTGCTCACCACCAGGTTGATATTGATTCAGAGGTATAAAATAATTTT

Twin-Strep-tag

GTTTAACTTTAAGAAGGAGATATACATATGTGGTCGCACCCGCAGTTCGAGAAAGGTGGAGGTTCCGGAGGT

XhoI

GGATCGGGAGGTTCGGCGTGGAGCCACCCGCAGTTCGAAAAAGCCTCGAGCCAACAAGAAGCGTTAGGAAT

pduA

GGTAGAAACCAAAGGCTTGACTGCAGCCATAGAGGCCGCAGATGCAATGGTGAAGTCAGCCAATGTAATGCT
GGTCGGCTACGAAAAAATTGGTTCGGGGCTGGTAACAGTCATTGTCCGCGGCGATGTTGGCGCAGTCAAAGC
AGCAACAGATGCAGGTGCCGCCGCAGCACGTAATGTGGGAGAAGTGAAAGCCGTACACGTCATCCCACGCCC
TCACACCGATGTAGAAAAAATCTTACCGAAGGGAATTAGCTAATGAGCAGCAATGAGCTGGTTGATCAGATCA

pduB

TGGCGCAGGTGATTGCTCGCGTGGCAACGCCGGAACAGCAGGCTATCCCTGAAAATAATCCTCCAACACGAGA
AACGGCTATGGCAGAGAAAAGCTGCAGTTTAACGGAGTTTGTCGGTACTGCGATTGGCGACACCGTCGGTCT
GGTAATCGCCAACGTGGACAGCGCCCTACTGGACGCAATGAAACTTGAAAAACGGTATCGCTCCATTGGCATC
CTTGGCGCGCGTACTGGTGCAGGCCCGCACATCATGGCCGCAGATGAAGCGGTAAAAGCCACCAATACTGAA
GTCGTCAGTATTGAGTTGCCACGTGATACCAAAGGCGGCGCGGGTCACGGTTCGCTGATTATTCTCGGCGGCA
ACGATGTTTCCGACGTGAAACGCGGAATTGAGGTTGCGCTGAAAGAACTGGATCGCACCTTTGGCGATGTGTA
TGCCAACGAAGCCGGTCACATCGAGATGCAATACACCGCACGCGCCAGCTACGCGCTGGAAAAAGCCTTTGGT
GCACCGATTGGCCGTGCCTGTGGCGTGATCGTCGGCGCGCCGGCATCCGTTGGTGTCCTGATGGCTGATACTG
CGCTGAAATCCGCCAACGTGGAAGTTGTGGCCTACAGCTCCCCTGCCCATGGCACCAGCTTCAGTAACGAAGC
CATTCTGGTCATTTCAGGCGATTCCGGCGCTGTGCGTCAGGCCGTTATCTCCGCCCGCGAAATCGGTAAAACCG
TACTCGGGACCCTCGGCTCAGAACCGAAAAACGATCGTCCGTCCTACATCTGAACTAGAAATAATTTTGTTTAA

pduJ

CTTTAAGAAGGAGATATACATATGAATAACGCACTGGGACTGGTTGAAACAAAAGGGCTCGTCGGCGCTATTG
AAGCCGCTGATGCCATGGTGAAATCCGCAAACGTGCAGTTGGTTGGTTACGAAAAAATCGGCTCAGGCCTTAT
CACCGTTATGGTTCGCGGCGATGTCGGCGCAGTGAAAGCTGCCGTAGATGCAGGAAGCGCTGCAGCAAGCGC
CGTTGGCGAGGTGAAATCCTGCCACGTTATCCCGCGTCCGCACAGCGACGTTGAAGCCATTTTACCTAAATCCG
CATAAATACTAGAAATAATTTTGTTTAACTTTAAGAAGGAGATATACATATGGTGAAGCAATCACTGGGATTAC

pduK

TTGAAGTTAGTGGTCTGGCATTAGCCATCAGTTGCGCGGACGTCATGGCGAAAGCCGCCTCCATCACGCTGGT
GGGCCTCGAAAAAACCAACGGTTCAGGCTGGATGGTGATCAAGATAATCGGGGATGTGGCCTCCGTCCAGGC
GGCCATTTCCACCGGTGTCAGTTTCGCTGACCAGCGAGATGGACTGGTGGCTCACAAAGTCATATCCAGACCA
GGGGATGGCATTCTGTCACATAGCGTCACCCCGGAGTCTGAGTCTGAGCCAGCGCCCGCCCCGACACCGGTCG
TACCACATGAAGAGATCCCTGAGGACCATGCAGCGCCCGAAGCGCCACAAGATGCAGAGTTGATTAGCTGCA
ATCTGTGTCTTGACCCTGCCTGCCCCCGTCAAAAGGGCGAGCCGCGCTCTCTTTGTCTGCACTCAGGCAAACGA
GGTGAAGCGTGAACTAGAAATAATTTTGTTTAACTTTAAGAAGGAGATATACATATGCATCTGGCACGGGTTA

pduN

CAGGCGTTGTGGTTTCCACGCAAAAATCTCCATCACTGGTGGGGAAGAAACTGTTGCTGGTACGTCGGGTGAG
TGCGGACGGAGAACTTCCCGCGTCCCCTGTGAGTGGAGATGAAGTCGCGGTTGATTCTGTCGGCGCTGGAACT

GGAGAACTGGTATTACTCAGCAGTGGCTCCAGCGCCAGACACGTTTTTTCCGGCCCTAATGAGGCCATCGATCT
GGCTATCGTCGGCATTGTCGACACGCTTTCTCGTTAGACTAGAAATAATTTTGTTTAACTTTAAGAAGGAGATA

pduU

TACATATGGAAAGACAACCCACCACGGATCGTATGATTCAGGAATATGTTCCTGGCAAGCAGGTTACGCTGGC
GCATCTTATCGCTAATCCAGGTAAAGATTTGTTCAAGAAACTGGGATTACCAGAGTCGGTTTCCGCAATCGGTA
TTCTGACGATTACCCCCAGCGAAGCCTCAATCATCGCCTGTGATATCGCCACGAAATCCGGGGCGGTAGAGAT
TGGTTTTCTCGACCGTTTTACCGGCGCAGTGGTACTGACAGGCGATGTTTCCGCTGTTGAGTACGCGCTGAAAC

BglII

AGGTAACCCGGACGCTGGGCGAAATGATGCGTTTTACCGCCTGTCCCATCACCCGGACATAAAGATCTTTCTCC

pduD$_{(1\text{-}18)}$

ATACCCGTTTTTTTGGGCTAGCAGGAGGGTACCTATATGGAAATCAATGAAAAGCTGCTGCGCCAGATTATTG

lacI

AAGACGTACTGTCTGAAAAACCAGTAACGTTATACGATGTCGCAGAGTATGCCGGTGTCTCTTATCAGACCGTT
TCCCGCGTGGTGAACCAGGCCAGCCACGTTTCTGCGAAAACGCGGGAAAAAGTGGAAGCGGCGATGGCGGA
GCTGAATTACATTCCCAACCGCGTGGCACAACAACTGGCGGGCAAACAGTCGTTGCTGATTGGCGTTGCCACC
TCCAGTCTGGCCCTGCACGCGCCGTCGCAAATTGTCGCGGCGATTAAATCTCGCGCCGATCAACTGGGTGCCA
GCGTGGTGGTGTCGATGGTAGAACGAAGCGGCGTCGAAGCCTGTAAAGCGGCGGTGCACAATCTTCTCGCGC
AACGCGTCAGTGGGCTGATCATTAACTATCCGCTGGATGACCAGGATGCCATTGCTGTGGAAGCTGCCTGCAC
TAATGTTCCGGCGTTATTTCTTGATGTCTCTGACCAGACACCCATCAACAGTATTATTTTCTCCCATGAAGACGG
TACGCGACTGGGCGTGGAGCATCTGGTCGCATTGGGTCACCAGCAAATCGCGCTGTTAGCGGGCCCATTAAGT
TCTGTCTCGGCGCGTCTGCGTCTGGCTGGCTGGCATAAATATCTCACTCGCAATCAAATTCAGCCGATAGCGGA
ACGGGAAGGCGACTGGAGTGCCATGTCCGGTTTTCAACAAACCATGCAAATGCTGAATGAGGGCATCGTTCCC
ACTGCGATGCTGGTTGCCAACGATCAGATGGCGCTGGGCGCAATGCGCGCCATTACCGAGTCCGGGCTGCGC
GTTGGTGCGGATATCTCGGTAGTGGGATACGACGATACCGAAGACAGCTCATGTTATATCCCGCCGTTAACCA
CCATCAAACAGGATTTTCGCCTGCTGGGGCAAACCAGCGTGGACCGCTTGCTGCAACTCTCTCAGGGCCAGGC
GGTGAAGGGCAATCAGCTGTTGCCCGTCTCACTGGTGAAAAGAAAAACCACCCTGGCGCCCAATACGCAAACC
GCCTCTCCCCGCGCGTTGGCCGATTCATTAATGCAGCTGGCACGACAGGTTTCCCGACTGGAAAGCGGGCAGC

SmaI *EGFP*

CCGGGAGCAAGGGCGAGGAGCTGTTCACCGGGGTGGTGCCCATCCTGGTCGAGCTGGACGGCGACGTAAAC
GGCCACAAGTTCAGCGTGTCCGGCGAGGGCGAGGGCGATGCCACCTACGGCAAGCTGACCCTGAAGTTCATC
TGCACCACCGGCAAGCTGCCCGTGCCCTGGCCCACCCTCGTGACCACCTTGACCTACGGCGTGCAGTGCTTCGC
CCGCTACCCCGACCACATGAAGCAGCACGACTTCTTCAAGTCCGCCATGCCCGAAGGCTACGTCCAGGAGCGC
ACCATCTTCTTCAAGGACGACGGCAACTACAAGACCCGCGCCGAGGTGAAGTTCGAGGGCGACACCCTGGTG
AACCGCATCGAGCTGAAGGGCATCGACTTCAAGGAGGACGGCAACATCCTGGGGCACAAGCTGGAGTACAAC
TACAACAGCCACAAGGTCTATATCACCGCCGACAAGCAGAAGAACGGCATCAAGGTGAACTTCAAGACCCGCC
ACAACATCGAGGACGGCAGCGTGCAGCTCGCCGACCACTACCAGCAGAACACCCCCATCGGCGACGGCCCCG
TGCTGCTGCCCGACAACCACTACCTGAGCACCCAGTCCGCCCTGAGCAAAGACCCCAACGAGAAGCGCGATCA

BglII

CATGGTCCTGCTGGAGTTCGTGACCGCCGCCGGGATCACTCTCGGCATGGACGAGCTGTACAAGTAAAGATCT

SmaI

CCCGGGGGCTGTTTTGGCGGATGAGAGAAGATTTTCAGCCTGATACAGATTAAATCAGAACGCAGAAGCGGT
CTGATAAAACAGAATTTGCCTGGCGGCAGTAGCGCGGTGGTCCCACCTGACCCCATGCCGAACTCAGAAGTGA
AACGCCGTAGCGCCGATGGTAGTGTGGGGTCTCCCCATGCGAGAGTAGGGAACTGCCAGGCATCAAATAAAA

CGAAAGGCTCAGTCGAAAGACTGGGCCTTTCGTTTTATCTGTTGTTTGTCGGTGAACGCTCTCCTGAGTAGGAC
AAATCCGCCGGGAGCGGATTTGAACGTTGCGAAGCAACGGCCCGGAGGGTGGCGGGCAGGACGCCCGCCAT
AAACTGCCAGGCATCAAATTAAGCAGAAGGCCATCCTGACGGATGGCCTTTTTGCGTTTCTACAAACTCTTTTG
TTTATTTTTCTAAATACATTCAAATATGTATCCGCTCATGAGACAATAACCCTGATAAATGCTTCAATAATATTGA
AAAAGGAAGAGTATGAGTATTCAACATTTCCGTGTCGCCCTTATTCCCTTTTTTGCGGCATTTTGCCTTCCTGTTT
TTGCTCACCCAGAAACGCTGGTGAAAGTAAAAGATGCTGAAGATCAGTTGGGTGCACCGATGATAAGCTGTCA
AACATGAGAATTACAACTTATATCGTATGGGGCTGACTTCAGGTGCTACATTTGAAGAGATAAATTGCACTGAA
ATCTAGAAATATTTTATCTGATTAATAAGATGATCTTCTTGAGATCGTTTTGGTCTGCGCGTAATCTCTTGCTCT
GAAAACGAAAAAACCGCCTTGCAGGGCGGTTTTTCGAAGGTTCTCTGAGCTACCAACTCTTTGAACCGAGGTA
ACTGGCTTGGAGGAGCGCAGTCACCAAAACTTGTCCTTTCAGTTTAGCCTTAACCGGCGCATGACTTCAAGACT
AACTCCTCTAAATCAATTACCAGTGGCTGCTGCCAGTGGTGCTTTTGCATGTCTTTCCGGGTTGGACTCAAGAC
GATAGTTACCGGATAAGGCGCAGCGGTCGGACTGAACGGGGGGTTCGTGCATACAGTCCAGCTTGGAGCGAA
CTGCCTACCCGGAACTGAGTGTCAGGCGTGGAATGAGACAAACGCGGCCATAACAGCGGAATGACACCGGTA
AACCGAAAGGCAGGAACAGGAGAGCGCACGAGGGAGCCGCCAGGGGGAAACGCCTGGTATCTTTATAGTCC
TGTCGGGTTTCGCCACCACTGATTTGAGCGTCAGATTTCGTGATGCTTGTCAGGGGGGCGGAGCCTATGGAAA
AACGGCTTTGCCGCGGCCCTCTCACTTCCCTGTTAAGTATCTTCCTGGCATCTTCCAGGAAATCTCCGCCCCGTT
CGTAAGCCATTTCCGCTCGCCGCAGTCGAACGACCGAGCGTAGCGAGTCAGTGAGCGAGGAAGCGGAATATA
TCCTGTATCACATATTCTGCTGACGCACCGGTGCAGCCTTTTTTCTCCTGCCACATGAAGCACTTCACTGACACC
CTCATCAGTGCCAACATAGTAAGCCAGTATACACTCCGCTAGCGCTGATGTCCGGCGGTGCTTTTGCCGTTACG
CACCACCCCGTCAGTAGCTGAACAGGAGGGACAGCTGATAGAAACAGAAGCCACTGGAGCACCTCAAAAACA
CCATCATACACTAAATCAGTAAGTTGGCAGCATCACCCGACGCACTTTGCGCCGAATAAATACCTGTGACGGAA
GATCACTTCGCAGAATAAATAAATCCTGGTGTCCCTGTTGATACCGGGAAGCCCTGGGCCAACTTTTGGCGAA
AATGAGACGTTGATCGGCACGTAAGAGGTTCCAACTTTCACCATAATGAAATAAGATCACTACCGGGCGTATT
TTTGAGTTATCGAGATTTTCAGGAGCTAAGGAAGCTAAAATGGAGAAAAAAATCACTGGATATACCACCGTT
GATATATCCCAATGGCATCGTAAAGAACATTTTGAGGCATTTCAGTCAGTTGCTCAATGTACCTATAACCAGAC
CGTTCAGCTGGATATTACGGCCTTTTTAAAGACCGTAAAGGAAAATAAGCACAAGTTTTATCCGGCCTTTATTC
ACATTCTTGCCCGCCTGATGAATGCTCATCCGGAATTCCGTATGGCAATGAAAGACGGTGAGCTGGTGATATG
GGATAGTGTTCACCCTTGTTACACCGTTTTCCATGAGCAAACTGAAACGTTTTCATCGCTCTGGAGTGAATACC
ACGACGATTTCCGGCAGTTTCTACACATATATTCGCAAGATGTGGCGTGTTACGGTGAAAACCTGGCCTATTTC
CCTAAAGGGTTTATTGAGAATATGTTTTTCGTCTCAGCCAATCCCTGGGTGAGTTTCACCAGTTTTGATTTAAAC
GTGGCCAATATGGACAACTTCTTCGCCCCCGTTTTCACCATGGGCAAATATTATACGCAAGGCGACAAGGT
GCTGATGCCGCTGGCGATTCAGGTTCATCATGCCGTTTGTGATGGCTTCCATGTCGGCAGAATGCTTAATGAAT
TACAACAGTACTGCGATGAGTGGCAGGGCGGGGCGTAATTTTTTTAAGGCAGTTATTGGTGCCCTTAAACGCC
TGGTTGCTACGCCTGAATAAGTGATAATAAGCGGATGAATGGCAG

>Complete sequence of the pOHΔTwin-Strep-tag plasmid. Features of the plasmid are shown in different colours: shown in orange, blue and green are genes *I-SceI, gam, pduABJKNU,* $pduD_{(1-18)}$*-lacI-EGFP* and *araBAD* promoter. Target sites for SphI, XhoI, SmaI and BglII restriction enzymes are shown in red.

GTGCACTCTCAGTACAATCTGCTCTGATGCCGCATAGTTAAGCCAGTATACACTCCGCTATCGCTACGTGACTG
GGTCATGGCTGCGCCCCGACACCCGCCAACACCCGCTGACGCGCCCTGACGGGCTTGTCTGCTCCCGGCATCC
GCTTACAGACAAGCTGTGACCGTCTCCGGGAGCTGCATGTGTCAGAGGTTTTCACCGTCATCACCGAAACGCG
CGAGGCAGCAAGGAGATGGCGCCCAACAGTCCCCCGGCCACGGGGCCTGCCACCATACCCACGCCGAAACAA
GCGCTCATGAGCCCGAAGTGGCGAGCCCGATCTTCCCCATCGGTGATGTCGGCGATATAGGCGCCAGCAACC
GCACCTGTGGCGCCGGTGATGCCGGCCACGATGCGTCCGGCGTAGAGGATCTGCTCATGTTTGACAGCTTATC
ATCGATGCATAATGTGCCTGTCAAATGGACGAAGCAGGGATTCTGCAAACCCTATGCTACTCCGTCAAGCCGT
CAATTGTCTGATTCGTTACCAATTATGACAACTTGACGGCTACATCATTCACTTTTTCTTCACAACCGGCACGGA
ACTCGCTCGGGCTGGCCCCGGTGCATTTTTTAAATACCCGCGAGAAATAGAGTTGATCGTCAAAACCAACATTG
CGACCGACGGTGGCGATAGGCATCCGGGTGGTGCTCAAAAGCAGCTTCGCCTGGCTGATACGTTGGTCCTCGC
GCCAGCTTAAGACGCTAATCCCTAACTGCTGGCGGAAAAGATGTGACAGACGCGACGGCGACAAGCAAACAT
GCTGTGCGACGCTGGCGATATCAAAATTGCTGTCTGCCAGGTGATCGCTGATGTACTGACAAGCCTCGCGTAC
CCGATTATCCATCGGTGGATGGAGCGACTCGTTAATCGCTTCCATGCGCCGCAGTAACAATTGCTCAAGCAGAT
TTATCGCCAGCAGCTCCGAATAGCGCCCTTCCCCTTGCCCGGCGTTAATGATTTGCCCAAACAGGTCGCTGAAA
TGCGGCTGGTGCGCTTCATCCGGGCGAAAGAACCCCGTATTGGCAAATATTGACGGCCAGTTAAGCCATTCAT
GCCAGTAGGCGCGCGGACGAAAGTAAACCCACTGGTGATACCATTCGCGAGCCTCCGGATGACGACCGTAGT
GATGAATCTCTCCTGGCGGGAACAGCAAAATATCACCCGGTCGGCAAACAAATTCTCGTCCCTGATTTTTCACC
ACCCCCTGACCGCGAATGGTGAGATTGAGAATATAACCTTTCATTCCCAGCGGTCGGTCGATAAAAAAATCGA
GATAACCGTTGGCCTCAATCGGCGTTAAACCCGCCACCAGATGGGCATTAAACGAGTATCCCGGCAGCAGGG
GATCATTTTGCGCTTCAGCCATACTTTTCATACTCCCGCCATTCAGAGAAGAAACCAATTGTCCATATTGCATCA
GACATTGCCGTCACTGCGTCTTTTACTGGCTCTTCTCGCTAACCAAACCGGTAACCCCGCTTATTAAAAGCATTC
TGTAACAAAGCGGGACCAAAGCCATGACAAAAACGCGTAACAAAAGTGTCTATAATCACGGCAGAAAAGTCC

araBAD promoter

ACATTGATTATTTGCACGGCGTCACACTTTGCTATGCCATAGCATTTTTATCCATAAGATTAGCGGATCCTACCT

GACGCTTTTTATCGCAACTCTCTACTGTTTCTCCATACCCGTTTTTTTGGGCTAGCAGGAGGGTACCTATATGCA

I-SceI

TATGAAAAACATCAAAAAAAACCAGGTAATGAACCTGGGTCCGAACTCTAAACTGCTGAAAGAATACAAATCC
CAGCTGATCGAACTGAACATCGAACAGTTCGAAGCAGGTATCGGTCTGATCCTGGGTGATGCTTACATCCGTT
CTCGTGATGAAGGTAAAACCTACTGTATGCAGTTCGAGTGGAAAAACAAAGCATACATGGACCACGTATGTCT
GCTGTACGATCAGTGGGTACTGTCCCCGCCGCACAAAAAAGAACGTGTTAACCACCTGGGTAACCTGGTAATC
ACCTGGGGCGCCCAGACTTTCAAACACCAAGCTTTCAACAAACTGGCTAACCTGTTCATCGTTAACAACAAAAA
AACCATCCCGAACAACCTGGTTGAAAACTACCTGACCCCGATGTCTCTGGCATACTGGTTCATGGATGATGGTG
GTAAATGGGATTACAACAAAAACTCTACCAACAAATCGATCGTACTGAACACCCAGTCTTTCACTTTCGAAGAA
GTAGAATACCTGGTTAAGGGTCTGCGTAACAAATTCCAACTGAACTGTTACGTAAAAATCAACAAAAACAAAC
CGATCATCTACATCGATTCTATGTCTTACCTGATCTTCTACAACCTGATCAAACCGTACCTGATCCCGCAGATGA

SphI

TGTACAAACTGCCGAACACTATCTCCTCCGAAACTTTCCTGAAATAAGGGCCCGACGTCTGCAGGCATGCTAAG

gam

GAGGTTATAAAAAATGGATATTAATACTGAAACTGAGATCAAGCAAAAGCATTCACTAACCCCCTTTCCTGTTT
TCCTAATCAGCCCGGCATTTCGCGGGCGATATTTTCACAGCTATTTCAGGAGTTCAGCCATGAACGCTTATTAC

ATTCAGGATCGTCTTGAGGCTCAGAGCTGGGCGCGTCACTACCAGCAGCTCGCCCGTGAAGAGAAAGAGGCA
GAACTGGCAGACGACATGGAAAAAGGCCTGCCCCAGCACCTGTTTGAATCGCTATGCATCGATCATTTGCAAC
GCCACGGGGCCAGCAAAAAATCCATTACCCGTGCGTTTGATGACGATGTTGAGTTTCAGGAGCGCATGGCAG

NotI

AACACATCCGGTACATGGTTGAAACCATTGCTCACCACCAGGTTGATATTGATTCAGAGGTATAAGCGGCCGC

pduA

AATAATTTTGTTTAACTTTAAGAAGGAGATATACATCAACAAGAAGCGTTAGGAATGGTAGAAACCAAAGGCT
TGACTGCAGCCATAGAGGCCGCAGATGCAATGGTGAAGTCAGCCAATGTAATGCTGGTCGGCTACGAAAAAA
TTGGTTCGGGGCTGGTAACAGTCATTGTCCGCGGCGATGTTGGCGCAGTCAAAGCAGCAACAGATGCAGGTG
CCGCCGCAGCACGTAATGTGGGAGAAGTGAAAGCCGTACACGTCATCCCACGCCCTCACACCGATGTAGAAAA
AATCTTACCGAAGGGAATTAGCTAATGAGCAGCAATGAGCTGGTTGATCAGATCA

pduB

TGGCGCAGGTGATTGCTCGCGTGGCAACGCCGGAACAGCAGGCTATCCCTGAAAATAATCCTCCAACACGAGA
AACGGCTATGGCAGAGAAAAGCTGCAGTTTAACGGAGTTTGTCGGTACTGCGATTGGCGACACCGTCGGTCT
GGTAATCGCCAACGTGGACAGCGCCCTACTGGACGCAATGAAACTTGAAAAACGGTATCGCTCCATTGGCATC
CTTGGCGCGCGTACTGGTGCAGGCCCGCACATCATGGCCGCAGATGAAGCGGTAAAAGCCACCAATACTGAA
GTCGTCAGTATTGAGTTGCCACGTGATACCAAAGGCGGCGCGGGTCACGGTTCGCTGATTATTCTCGGCGGCA
ACGATGTTTCCGACGTGAAACGCGGAATTGAGGTTGCGCTGAAAGAACTGGATCGCACCTTTGGCGATGTGTA
TGCCAACGAAGCCGGTCACATCGAGATGCAATACACCGCACGCGCCAGCTACGCGCTGGAAAAAGCCTTTGGT
GCACCGATTGGCCGTGCCTGTGGCGTGATCGTCGGCGCGCCGGCATCCGTTGGTGTCCTGATGGCTGATACTG
CGCTGAAATCCGCCAACGTGGAAGTTGTGGCCTACAGCTCCCCTGCCCATGGCACCAGCTTCAGTAACGAAGC
CATTCTGGTCATTTCAGGCGATTCCGGCGCTGTGCGTCAGGCCGTTATCTCCGCCCGCGAAATCGGTAAAACCG
TACTCGGGACCCTCGGCTCAGAACCGAAAAACGATCGTCCGTCCTACATCTGAACTAGAAATAATTTTGTTTAA

pduJ

CTTTAAGAAGGAGATATACATATGAATAACGCACTGGGACTGGTTGAAACAAAAGGGCTCGTCGGCGCTATTG
AAGCCGCTGATGCCATGGTGAAATCCGCAAACGTGCAGTTGGTTGGTTACGAAAAAATCGGCTCAGGCCTTAT
CACCGTTATGGTTCGCGGCGATGTCGGCGCAGTGAAAGCTGCCGTAGATGCAGGAAGCGCTGCAGCAAGCGC
CGTTGGCGAGGTGAAATCCTGCCACGTTATCCCGCGTCCGCACAGCGACGTTGAAGCCATTTTACCTAAATCCG
CATAAATACTAGAAATAATTTTGTTTAACTTTAAGAAGGAGATATACATATGGTGAAGCAATCACTGGGATTAC

pduK

TTGAAGTTAGTGGTCTGGCATTAGCCATCAGTTGCGCGGACGTCATGGCGAAAGCCGCCTCCATCACGCTGGT
GGGCCTCGAAAAAACCAACGGTTCAGGCTGGATGGTGATCAAGATAATCGGGGATGTGGCCTCCGTCCAGGC
GGCCATTTCCACCGGTGTCAGTTTCGCTGACCAGCGAGATGGACTGGTGGCTCACAAAGTCATATCCAGACCA
GGGGATGGCATTCTGTCACATAGCGTCACCCCGGAGTCTGAGTCTGAGCCAGCGCCCGCCCCGACACCGGTCG
TACCACATGAAGAGATCCCTGAGGACCATGCAGCGCCCGAAGCGCCACAAGATGCAGAGTTGATTAGCTGCA
ATCTGTGTCTTGACCCTGCCTGCCCCCGTCAAAAGGGCGAGCCGCGCTCTCTTTGTCTGCACTCAGGCAAACGA
GGTGAAGCGTGAACTAGAAATAATTTTGTTTAACTTTAAGAAGGAGATATACATATGCATCTGGCACGGGTTA

pduN

CAGGCGTTGTGGTTTCCACGCAAAAATCTCCATCACTGGTGGGGAAGAAACTGTTGCTGGTACGTCGGGTGAG
TGCGGACGGAGAACTTCCCGCGTCCCCTGTGAGTGGAGATGAAGTCGCGGTTGATTCTGTCGGCGCTGGAACT
GGAGAACTGGTATTACTCAGCAGTGGCTCCAGCGCCAGACACGTTTTTTCCGGCCCTAATGAGGCCATCGATCT
GGCTATCGTCGGCATTGTCGACACGCTTTCTCGTTAGACTAGAAATAATTTTGTTTAACTTTAAGAAGGAGATA

pduU

TACATATGGAAAGACAACCCACCACGGATCGTATGATTCAGGAATATGTTCCTGGCAAGCAGGTTACGCTGGC
GCATCTTATCGCTAATCCAGGTAAAGATTTGTTCAAGAAACTGGGATTACCAGAGTCGGTTTCCGCAATCGGTA
TTCTGACGATTACCCCCAGCGAAGCCTCAATCATCGCCTGTGATATCGCCACGAAATCCGGGGCGGTAGAGAT
TGGTTTTCTCGACCGTTTTACCGGCGCAGTGGTACTGACAGGCGATGTTTCCGCTGTTGAGTACGCGCTGAAAC

BglII

AGGTAACCCGGACGCTGGGCGAAATGATGCGTTTTACCGCCTGTCCCATCACCCGGACATAAAGATCTTTCTCC

pduD$_{(1-18)}$

ATACCCGTTTTTTTGGGCTAGCAGGAGGGTACCTATATGGAAATCAATGAAAAGCTGCTGCGCCAGATTATTG

lacI

AAGACGTACTGTCTGAAAAACCAGTAACGTTATACGATGTCGCAGAGTATGCCGGTGTCTCTTATCAGACCGTT
TCCCGCGTGGTGAACCAGGCCAGCCACGTTTCTGCGAAAACGCGGGAAAAAGTGGAAGCGGCGATGGCGGA
GCTGAATTACATTCCCAACCGCGTGGCACAACAACTGGCGGGCAAACAGTCGTTGCTGATTGGCGTTGCCACC
TCCAGTCTGGCCCTGCACGCGCCGTCGCAAATTGTCGCGGCGATTAAATCTCGCGCCGATCAACTGGGTGCCA
GCGTGGTGGTGTCGATGGTAGAACGAAGCGGCGTCGAAGCCTGTAAAGCGGCGGTGCACAATCTTCTCGCGC
AACGCGTCAGTGGGCTGATCATTAACTATCCGCTGGATGACCAGGATGCCATTGCTGTGGAAGCTGCCTGCAC
TAATGTTCCGGCGTTATTTCTTGATGTCTCTGACCAGACACCCATCAACAGTATTATTTTCTCCCATGAAGACGG
TACGCGACTGGGCGTGGAGCATCTGGTCGCATTGGGTCACCAGCAAATCGCGCTGTTAGCGGGCCCATTAAGT
TCTGTCTCGGCGCGTCTGCGTCTGGCTGGCTGGCATAAATATCTCACTCGCAATCAAATTCAGCCGATAGCGGA
ACGGGAAGGCGACTGGAGTGCCATGTCCGGTTTTCAACAAACCATGCAAATGCTGAATGAGGGCATCGTTCCC
ACTGCGATGCTGGTTGCCAACGATCAGATGGCGCTGGGCGCAATGCGCGCCATTACCGAGTCCGGGCTGCGC
GTTGGTGCGGATATCTCGGTAGTGGGATACGACGATACCGAAGACAGCTCATGTTATATCCCGCCGTTAACCA
CCATCAAACAGGATTTTCGCCTGCTGGGGCAAACCAGCGTGGACCGCTTGCTGCAACTCTCTCAGGGCCAGGC
GGTGAAGGGCAATCAGCTGTTGCCCGTCTCACTGGTGAAAAGAAAAACCACCCTGGCGCCCAATACGCAAACC
GCCTCTCCCCGCGCGTTGGCCGATTCATTAATGCAGCTGGCACGACAGGTTTCCCGACTGGAAAGCGGGCAGC

SmaI *EGFP*

CCGGGAGCAAGGGCGAGGAGCTGTTCACCGGGGTGGTGCCCATCCTGGTCGAGCTGGACGGCGACGTAAAC
GGCCACAAGTTCAGCGTGTCCGGCGAGGGCGAGGGCGATGCCACCTACGGCAAGCTGACCCTGAAGTTCATC
TGCACCACCGGCAAGCTGCCCGTGCCCTGGCCCACCCTCGTGACCACCTTGACCTACGGCGTGCAGTGCTTCGC
CCGCTACCCCGACCACATGAAGCAGCACGACTTCTTCAAGTCCGCCATGCCCGAAGGCTACGTCCAGGAGCGC
ACCATCTTCTTCAAGGACGACGGCAACTACAAGACCCGCGCCGAGGTGAAGTTCGAGGGCGACACCCTGGTG
AACCGCATCGAGCTGAAGGGCATCGACTTCAAGGAGGACGGCAACATCCTGGGGCACAAGCTGGAGTACAAC
TACAACAGCCACAAGGTCTATATCACCGCCGACAAGCAGAAGAACGGCATCAAGGTGAACTTCAAGACCCGCC
ACAACATCGAGGACGGCAGCGTGCAGCTCGCCGACCACTACCAGCAGAACACCCCCATCGGCGACGGCCCCG
TGCTGCTGCCCGACAACCACTACCTGAGCACCCAGTCCGCCCTGAGCAAAGACCCCAACGAGAAGCGCGATCA

BglII

CATGGTCCTGCTGGAGTTCGTGACCGCCGCCGGGATCACTCTCGGCATGGACGAGCTGTACAAGTAAAGATCT

SmaI

CCCGGGGGCTGTTTTGGCGGATGAGAGAAGATTTTCAGCCTGATACAGATTAAATCAGAACGCAGAAGCGGT
CTGATAAAACAGAATTTGCCTGGCGGCAGTAGCGCGGTGGTCCCACCTGACCCCATGCCGAACTCAGAAGTGA
AACGCCGTAGCGCCGATGGTAGTGTGGGGTCTCCCCATGCGAGAGTAGGGAACTGCCAGGCATCAAATAAAA
CGAAAGGCTCAGTCGAAAGACTGGGCCTTTCGTTTTATCTGTTGTTTGTCGGTGAACGCTCTCCTGAGTAGGAC
AAATCCGCCGGGAGCGGATTTGAACGTTGCGAAGCAACGGCCCGGAGGGTGGCGGGCAGGACGCCCGCCAT
AAACTGCCAGGCATCAAATTAAGCAGAAGGCCATCCTGACGGATGGCCTTTTTGCGTTTCTACAAACTCTTTTG

TTTATTTTTCTAAATACATTCAAATATGTATCCGCTCATGAGACAATAACCCTGATAAATGCTTCAATAATATTGA
AAAAGGAAGAGTATGAGTATTCAACATTTCCGTGTCGCCCTTATTCCCTTTTTTGCGGCATTTTGCCTTCCTGTTT
TTGCTCACCCAGAAACGCTGGTGAAAGTAAAAGATGCTGAAGATCAGTTGGGTGCACCGATGATAAGCTGTCA
AACATGAGAATTACAACTTATATCGTATGGGGCTGACTTCAGGTGCTACATTTGAAGAGATAAATTGCACTGAA
ATCTAGAAATATTTTATCTGATTAATAAGATGATCTTCTTGAGATCGTTTTGGTCTGCGCGTAATCTCTTGCTCT
GAAAACGAAAAAACCGCCTTGCAGGGCGGTTTTTCGAAGGTTCTCTGAGCTACCAACTCTTTGAACCGAGGTA
ACTGGCTTGGAGGAGCGCAGTCACCAAAACTTGTCCTTTCAGTTTAGCCTTAACCGGCGCATGACTTCAAGACT
AACTCCTCTAAATCAATTACCAGTGGCTGCTGCCAGTGGTGCTTTTGCATGTCTTTCCGGGTTGGACTCAAGAC
GATAGTTACCGGATAAGGCGCAGCGGTCGGACTGAACGGGGGGTTCGTGCATACAGTCCAGCTTGGAGCGAA
CTGCCTACCCGGAACTGAGTGTCAGGCGTGGAATGAGACAAACGCGGCCATAACAGCGGAATGACACCGGTA
AACCGAAAGGCAGGAACAGGAGAGCGCACGAGGGAGCCGCCAGGGGGAAACGCCTGGTATCTTTATAGTCC
TGTCGGGTTTCGCCACCACTGATTTGAGCGTCAGATTTCGTGATGCTTGTCAGGGGGGCGGAGCCTATGGAAA
AACGGCTTTGCCGCGGCCCTCTCACTTCCCTGTTAAGTATCTTCCTGGCATCTTCCAGGAAATCTCCGCCCCGTT
CGTAAGCCATTTCCGCTCGCCGCAGTCGAACGACCGAGCGTAGCGAGTCAGTGAGCGAGGAAGCGGAATATA
TCCTGTATCACATATTCTGCTGACGCACCGGTGCAGCCTTTTTTCTCCTGCCACATGAAGCACTTCACTGACACC
CTCATCAGTGCCAACATAGTAAGCCAGTATACACTCCGCTAGCGCTGATGTCCGGCGGTGCTTTTGCCGTTACG
CACCACCCCGTCAGTAGCTGAACAGGAGGGACAGCTGATAGAAACAGAAGCCACTGGAGCACCTCAAAAACA
CCATCATACACTAAATCAGTAAGTTGGCAGCATCACCCGACGCACTTTGCGCCGAATAAATACCTGTGACGGAA
GATCACTTCGCAGAATAAATAAATCCTGGTGTCCCTGTTGATACCGGGAAGCCCTGGGCCAACTTTTGGCGAA
AATGAGACGTTGATCGGCACGTAAGAGGTTCCAACTTTCACCATAATGAAATAAGATCACTACCGGGCGTATT
TTTGAGTTATCGAGATTTTCAGGAGCTAAGGAAGCTAAAATGGAGAAAAAAATACACTGGATATACCACCGTT
GATATATCCCAATGGCATCGTAAAGAACATTTTGAGGCATTTCAGTCAGTTGCTCAATGTACCTATAACCAGAC
CGTTCAGCTGGATATTACGGCCTTTTTAAAGACCGTAAAGGAAAATAAGCACAAGTTTTATCCGGCCTTTATTC
ACATTCTTGCCCGCCTGATGAATGCTCATCCGGAATTCCGTATGGCAATGAAAGACGGTGAGCTGGTGATATG
GGATAGTGTTCACCCTTGTTACACCGTTTTCCATGAGCAAACTGAAACGTTTTCATCGCTCTGGAGTGAATACC
ACGACGATTTCCGGCAGTTTCTACACATATATTCGCAAGATGTGGCGTGTTACGGTGAAAACCTGGCCTATTTC
CCTAAAGGGTTTATTGAGAATATGTTTTTCGTCTCAGCCAATCCCTGGGTGAGTTTCACCAGTTTTGATTTAAAC
GTGGCCAATATGGACAACTTCTTCGCCCCCCCCGTTTTCACCATGGGCAAATATTATACGCAAGGCGACAAGGT
GCTGATGCCGCTGGCGATTCAGGTTCATCATGCCGTTTGTGATGGCTTCCATGTCGGCAGAATGCTTAATGAAT
TACAACAGTACTGCGATGAGTGGCAGGGCGGGGCGTAATTTTTTTAAGGCAGTTATTGGTGCCCTTAAACGCC
TGGTTGCTACGCCTGAATAAGTGATAATAAGCGGATGAATGGCAG

>Complete sequence of the pOHΔPduD plasmid. Features of the plasmid are shown in different colours: shown in orange, blue and green are genes *I-SceI, gam, Twin-Strep-tag – pduA, pduBJKNU, lacI-EGFP* and *araBAD* promoter. Target sites for SphI, XhoI, SmaI and BglII restriction enzymes are shown in red.

GTGCACTCTCAGTACAATCTGCTCTGATGCCGCATAGTTAAGCCAGTATACACTCCGCTATCGCTACGTGACTG
GGTCATGGCTGCGCCCCGACACCCGCCAACACCCGCTGACGCGCCCTGACGGGCTTGTCTGCTCCCGGCATCC
GCTTACAGACAAGCTGTGACCGTCTCCGGGAGCTGCATGTGTCAGAGGTTTTCACCGTCATCACCGAAACGCG
CGAGGCAGCAAGGAGATGGCGCCCAACAGTCCCCCGGCCACGGGGCCTGCCACCATACCCACGCCGAAACAA
GCGCTCATGAGCCCGAAGTGGCGAGCCCGATCTTCCCCATCGGTGATGTCGGCGATATAGGCGCCAGCAACC
GCACCTGTGGCGCCGGTGATGCCGGCCACGATGCGTCCGGCGTAGAGGATCTGCTCATGTTTGACAGCTTATC
ATCGATGCATAATGTGCCTGTCAAATGGACGAAGCAGGGATTCTGCAAACCCTATGCTACTCCGTCAAGCCGT
CAATTGTCTGATTCGTTACCAATTATGACAACTTGACGGCTACATCATTCACTTTTTCTTCACAACCGGCACGGA
ACTCGCTCGGGCTGGCCCCGGTGCATTTTTTAAATACCCGCGAGAAATAGAGTTGATCGTCAAAACCAACATTG
CGACCGACGGTGGCGATAGGCATCCGGGTGGTGCTCAAAAGCAGCTTCGCCTGGCTGATACGTTGGTCCTCGC
GCCAGCTTAAGACGCTAATCCCTAACTGCTGGCGGAAAAGATGTGACAGACGCGACGGCGACAAGCAAACAT
GCTGTGCGACGCTGGCGATATCAAAATTGCTGTCTGCCAGGTGATCGCTGATGTACTGACAAGCCTCGCGTAC
CCGATTATCCATCGGTGGATGGAGCGACTCGTTAATCGCTTCCATGCGCCGCAGTAACAATTGCTCAAGCAGAT
TTATCGCCAGCAGCTCCGAATAGCGCCCTTCCCCTTGCCCGGCGTTAATGATTTGCCCAAACAGGTCGCTGAAA
TGCGGCTGGTGCGCTTCATCCGGGCGAAAGAACCCCGTATTGGCAAATATTGACGGCCAGTTAAGCCATTCAT
GCCAGTAGGCGCGCGGACGAAAGTAAACCCACTGGTGATACCATTCGCGAGCCTCCGGATGACGACCGTAGT
GATGAATCTCTCCTGGCGGGAACAGCAAAATATCACCCGGTCGGCAAACAAATTCTCGTCCCTGATTTTTCACC
ACCCCCTGACCGCGAATGGTGAGATTGAGAATATAACCTTTCATTCCCAGCGGTCGGTCGATAAAAAAATCGA
GATAACCGTTGGCCTCAATCGGCGTTAAACCCGCCACCAGATGGGCATTAAACGAGTATCCCGGCAGCAGGG
GATCATTTTGCGCTTCAGCCATACTTTTCATACTCCCGCCATTCAGAGAAGAAACCAATTGTCCATATTGCATCA
GACATTGCCGTCACTGCGTCTTTTACTGGCTCTTCTCGCTAACCAAACCGGTAACCCCGCTTATTAAAAGCATTC
TGTAACAAAGCGGGACCAAAGCCATGACAAAAACGCGTAACAAAAGTGTCTATAATCACGGCAGAAAAGTCC

araBAD promoter

ACATTGATTATTTGCACGGCGTCACACTTTGCTATGCCATAGCATTTTTATCCATAAGATTAGCGGATCCTACCT

GACGCTTTTTATCGCAACTCTCTACTGTTTCTCCATACCCGTTTTTTTGGGCTAGCAGGAGGGTACCTATATGCA

I-SceI

TATGAAAAACATCAAAAAAAACCAGGTAATGAACCTGGGTCCGAACTCTAAACTGCTGAAAGAATACAAATCC
CAGCTGATCGAACTGAACATCGAACAGTTCGAAGCAGGTATCGGTCTGATCCTGGGTGATGCTTACATCCGTT
CTCGTGATGAAGGTAAAACCTACTGTATGCAGTTCGAGTGGAAAAACAAAGCATACATGGACCACGTATGTCT
GCTGTACGATCAGTGGGTACTGTCCCCGCCGCACAAAAAAGAACGTGTTAACCACCTGGGTAACCTGGTAATC
ACCTGGGGCGCCCAGACTTTCAAACACCAAGCTTTCAACAAACTGGCTAACCTGTTCATCGTTAACAACAAAAA
AACCATCCCGAACAACCTGGTTGAAAACTACCTGACCCCGATGTCTCTGGCATACTGGTTCATGGATGATGGTG
GTAAATGGGATTACAACAAAAACTCTACCAACAAATCGATCGTACTGAACACCCAGTCTTTCACTTTCGAAGAA
GTAGAATACCTGGTTAAGGGTCTGCGTAACAAATTCCAACTGAACTGTTACGTAAAAATCAACAAAAACAAAC
CGATCATCTACATCGATTCTATGTCTTACCTGATCTTCTACAACCTGATCAAACCGTACCTGATCCCGCAGATGA

SphI

TGTACAAACTGCCGAACACTATCTCCTCCGAAACTTTCCTGAAATAAGGGCCCGACGTCTGCAGGCATGCTAAG

gam

GAGGTTATAAAAAATGGATATTAATACTGAAACTGAGATCAAGCAAAAGCATTCACTAACCCCCTTTCCTGTTT
TCCTAATCAGCCCGGCATTTCGCGGGCGATATTTTCACAGCTATTTCAGGAGTTCAGCCATGAACGCTTATTAC

ATTCAGGATCGTCTTGAGGCTCAGAGCTGGGCGCGTCACTACCAGCAGCTCGCCCGTGAAGAGAAAGAGGCA
GAACTGGCAGACGACATGGAAAAAGGCCTGCCCCAGCACCTGTTTGAATCGCTATGCATCGATCATTTGCAAC
GCCACGGGGCCAGCAAAAAATCCATTACCCGTGCGTTTGATGACGATGTTGAGTTTCAGGAGCGCATGGCAG
AACACATCCGGTACATGGTTGAAACCATTGCTCACCACCAGGTTGATATTGATTCAGAGGTATAAAATAATTTT

Twin-Strep-tag

GTTTAACTTTAAGAAGGAGATATACATATGTGGTCGCACCCGCAGTTCGAGAAAGGTGGAGGTTCCGGAGGT

XhoI

GGATCGGGAGGTTCGGCGTGGAGCCACCCGCAGTTCGAAAAAGCCTCGAGCCAACAAGAAGCGTTAGGAAT

pduA

GGTAGAAACCAAAGGCTTGACTGCAGCCATAGAGGCCGCAGATGCAATGGTGAAGTCAGCCAATGTAATGCT
GGTCGGCTACGAAAAAATTGGTTCGGGGCTGGTAACAGTCATTGTCCGCGGCGATGTTGGCGCAGTCAAAGC
AGCAACAGATGCAGGTGCCGCCGCAGCACGTAATGTGGGAGAAGTGAAAGCCGTACACGTCATCCCACGCCC
TCACACCGATGTAGAAAAAATCTTACCGAAGGGAATTAGCTAATGAGCAGCAATGAGCTGGTTGATCAGATCA

pduB

TGGCGCAGGTGATTGCTCGCGTGGCAACGCCGGAACAGCAGGCTATCCCTGAAAATAATCCTCCAACACGAGA
AACGGCTATGGCAGAGAAAAGCTGCAGTTTAACGGAGTTTGTCGGTACTGCGATTGGCGACACCGTCGGTCT
GGTAATCGCCAACGTGGACAGCGCCCTACTGGACGCAATGAAACTTGAAAAACGGTATCGCTCCATTGGCATC
CTTGGCGCGCGTACTGGTGCAGGCCCGCACATCATGGCCGCAGATGAAGCGGTAAAAGCCACCAATACTGAA
GTCGTCAGTATTGAGTTGCCACGTGATACCAAAGGCGGCGCGGGTCACGGTTCGCTGATTATTCTCGGCGGCA
ACGATGTTTCCGACGTGAAACGCGGAATTGAGGTTGCGCTGAAAGAACTGGATCGCACCTTTGGCGATGTGTA
TGCCAACGAAGCCGGTCACATCGAGATGCAATACACCGCACGCGCCAGCTACGCGCTGGAAAAAGCCTTTGGT
GCACCGATTGGCCGTGCCTGTGGCGTGATCGTCGGCGCGCCGGCATCCGTTGGTGTCCTGATGGCTGATACTG
CGCTGAAATCCGCCAACGTGGAAGTTGTGGCCTACAGCTCCCCTGCCCATGGCACCAGCTTCAGTAACGAAGC
CATTCTGGTCATTTCAGGCGATTCCGGCGCTGTGCGTCAGGCCGTTATCTCCGCCCGCGAAATCGGTAAAACCG
TACTCGGGACCCTCGGCTCAGAACCGAAAAACGATCGTCCGTCCTACATCTGAACTAGAAATAATTTTGTTTAA

pduJ

CTTTAAGAAGGAGATATACATATGAATAACGCACTGGGACTGGTTGAAACAAAAGGGCTCGTCGGCGCTATTG
AAGCCGCTGATGCCATGGTGAAATCCGCAAACGTGCAGTTGGTTGGTTACGAAAAAATCGGCTCAGGCCTTAT
CACCGTTATGGTTCGCGGCGATGTCGGCGCAGTGAAAGCTGCCGTAGATGCAGGAAGCGCTGCAGCAAGCGC
CGTTGGCGAGGTGAAATCCTGCCACGTTATCCCGCGTCCGCACAGCGACGTTGAAGCCATTTTACCTAAATCCG
CATAAATACTAGAAATAATTTTGTTTAACTTTAAGAAGGAGATATACATATGGTGAAGCAATCACTGGGATTAC

pduK

TTGAAGTTAGTGGTCTGGCATTAGCCATCAGTTGCGCGGACGTCATGGCGAAAGCCGCCTCCATCACGCTGGT
GGGCCTCGAAAAAACCAACGGTTCAGGCTGGATGGTGATCAAGATAATCGGGGATGTGGCCTCCGTCCAGGC
GGCCATTTCCACCGGTGTCAGTTTCGCTGACCAGCGAGATGGACTGGTGGCTCACAAAGTCATATCCAGACCA
GGGGATGGCATTCTGTCACATAGCGTCACCCCGGAGTCTGAGTCTGAGCCAGCGCCCGCCCCGACACCGGTCG
TACCACATGAAGAGATCCCTGAGGACCATGCAGCGCCCGAAGCGCCACAAGATGCAGAGTTGATTAGCTGCA
ATCTGTGTCTTGACCCTGCCTGCCCCCGTCAAAAGGGCGAGCCGCGCTCTCTTTGTCTGCACTCAGGCAAACGA
GGTGAAGCGTGAACTAGAAATAATTTTGTTTAACTTTAAGAAGGAGATATACATATGCATCTGGCACGGGTTA

pduN

CAGGCGTTGTGGTTTCCACGCAAAAATCTCCATCACTGGTGGGGAAGAAACTGTTGCTGGTACGTCGGGTGAG
TGCGGACGGAGAACTTCCCGCGTCCCCTGTGAGTGGAGATGAAGTCGCGGTTGATTCTGTCGGCGCTGGAACT

GGAGAACTGGTATTACTCAGCAGTGGCTCCAGCGCCAGACACGTTTTTTCCGGCCCTAATGAGGCCATCGATCT
GGCTATCGTCGGCATTGTCGACACGCTTTCTCGTTAGACTAGAAATAATTTTGTTTAACTTTAAGAAGGAGATA

pduU

TACATATGGAAAGACAACCCACCACGGATCGTATGATTCAGGAATATGTTCCTGGCAAGCAGGTTACGCTGGC
GCATCTTATCGCTAATCCAGGTAAAGATTTGTTCAAGAAACTGGGATTACCAGAGTCGGTTTCCGCAATCGGTA
TTCTGACGATTACCCCCAGCGAAGCCTCAATCATCGCCTGTGATATCGCCACGAAATCCGGGGCGGTAGAGAT
TGGTTTTCTCGACCGTTTTACCGGCGCAGTGGTACTGACAGGCGATGTTTCCGCTGTTGAGTACGCGCTGAAAC

BglII

AGGTAACCCGGACGCTGGGCGAAATGATGCGTTTTACCGCCTGTCCCATCACCCGGACATAAAGATCTACTAG

lacI

AAATAATTTTGTTTAACTTTAAGAAGGAGATATACATATGAAACCAGTAACGTTATACGATGTCGCAGAGTATG
CCGGTGTCTCTTATCAGACCGTTTCCCGCGTGGTGAACCAGGCCAGCCACGTTTCTGCGAAAACGCGGGAAAA
AGTGGAAGCGGCGATGGCGGAGCTGAATTACATTCCCAACCGCGTGGCACAACAACTGGCGGGCAAACAGTC
GTTGCTGATTGGCGTTGCCACCTCCAGTCTGGCCCTGCACGCGCCGTCGCAAATTGTCGCGGCGATTAAATCTC
GCGCCGATCAACTGGGTGCCAGCGTGGTGGTGTCGATGGTAGAACGAAGCGGCGTCGAAGCCTGTAAAGCG
GCGGTGCACAATCTTCTCGCGCAACGCGTCAGTGGGCTGATCATTAACTATCCGCTGGATGACCAGGATGCCA
TTGCTGTGGAAGCTGCCTGCACTAATGTTCCGGCGTTATTTCTTGATGTCTCTGACCAGACACCCATCAACAGTA
TTATTTTCTCCCATGAAGACGGTACGCGACTGGGCGTGGAGCATCTGGTCGCATTGGGTCACCAGCAAATCGC
GCTGTTAGCGGGCCCATTAAGTTCTGTCTCGGCGCGTCTGCGTCTGGCTGGCTGGCATAAATATCTCACTCGCA
ATCAAATTCAGCCGATAGCGGAACGGGAAGGCGACTGGAGTGCCATGTCCGGTTTTCAACAAACCATGCAAAT
GCTGAATGAGGGCATCGTTCCCACTGCGATGCTGGTTGCCAACGATCAGATGGCGCTGGGCGCAATGCGCGC
CATTACCGAGTCCGGGCTGCGCGTTGGTGCGGATATCTCGGTAGTGGGATACGACGATACCGAAGACAGCTC
ATGTTATATCCCGCCGTTAACCACCATCAAACAGGATTTTCGCCTGCTGGGGCAAACCAGCGTGGACCGCTTGC
TGCAACTCTCTCAGGGCCAGGCGGTGAAGGGCAATCAGCTGTTGCCCGTCTCACTGGTGAAAAGAAAAACCAC
CCTGGCGCCCAATACGCAAACCGCCTCTCCCCGCGCGTTGGCCGATTCATTAATGCAGCTGGCACGACAGGTTT
CCCGACTGGAAAGCGGGCAGC

SmaI *EGFP*

CCGGGAGCAAGGGCGAGGAGCTGTTCACCGGGGTGGTGCCCATCCTGGTCGAGCTGGACGGCGACGTAAAC
GGCCACAAGTTCAGCGTGTCCGGCGAGGGCGAGGGCGATGCCACCTACGGCAAGCTGACCCTGAAGTTCATC
TGCACCACCGGCAAGCTGCCCGTGCCCTGGCCCACCCTCGTGACCACCTTGACCTACGGCGTGCAGTGCTTCGC
CCGCTACCCCGACCACATGAAGCAGCACGACTTCTTCAAGTCCGCCATGCCCGAAGGCTACGTCCAGGAGCGC
ACCATCTTCTTCAAGGACGACGGCAACTACAAGACCCGCGCCGAGGTGAAGTTCGAGGGCGACACCCTGGTG
AACCGCATCGAGCTGAAGGGCATCGACTTCAAGGAGGACGGCAACATCCTGGGGCACAAGCTGGAGTACAAC
TACAACAGCCACAAGGTCTATATCACCGCCGACAAGCAGAAGAACGGCATCAAGGTGAACTTCAAGACCCGCC
ACAACATCGAGGACGGCAGCGTGCAGCTCGCCGACCACTACCAGCAGAACACCCCCATCGGCGACGGCCCCG
TGCTGCTGCCCGACAACCACTACCTGAGCACCCAGTCCGCCCTGAGCAAAGACCCCAACGAGAAGCGCGATCA

BglII

CATGGTCCTGCTGGAGTTCGTGACCGCCGCCGGGATCACTCTCGGCATGGACGAGCTGTACAAGTAAAGATCT

SmaI

CCCGGGGGCTGTTTTGGCGGATGAGAGAAGATTTTCAGCCTGATACAGATTAAATCAGAACGCAGAAGCGGT
CTGATAAAACAGAATTTGCCTGGCGGCAGTAGCGCGGTGGTCCCACCTGACCCCATGCCGAACTCAGAAGTGA
AACGCCGTAGCGCCGATGGTAGTGTGGGGTCTCCCCATGCGAGAGTAGGGAACTGCCAGGCATCAAATAAAA
CGAAAGGCTCAGTCGAAAGACTGGGCCTTTCGTTTTATCTGTTGTTTGTCGGTGAACGCTCTCCTGAGTAGGAC
AAATCCGCCGGGAGCGGATTTGAACGTTGCGAAGCAACGGCCCGGAGGGTGGCGGGCAGGACGCCCGCCAT

AAACTGCCAGGCATCAAATTAAGCAGAAGGCCATCCTGACGGATGGCCTTTTTGCGTTTCTACAAACTCTTTTG
TTTATTTTTCTAAATACATTCAAATATGTATCCGCTCATGAGACAATAACCCTGATAAATGCTTCAATAATATTGA
AAAAGGAAGAGTATGAGTATTCAACATTTCCGTGTCGCCCTTATTCCCTTTTTTGCGGCATTTTGCCTTCCTGTTT
TTGCTCACCCAGAAACGCTGGTGAAAGTAAAAGATGCTGAAGATCAGTTGGGTGCACCGATGATAAGCTGTCA
AACATGAGAATTACAACTTATATCGTATGGGGCTGACTTCAGGTGCTACATTTGAAGAGATAAATTGCACTGAA
ATCTAGAAATATTTTATCTGATTAATAAGATGATCTTCTTGAGATCGTTTTGGTCTGCGCGTAATCTCTTGCTCT
GAAAACGAAAAAACCGCCTTGCAGGGCGGTTTTTCGAAGGTTCTCTGAGCTACCAACTCTTTGAACCGAGGTA
ACTGGCTTGGAGGAGCGCAGTCACCAAAACTTGTCCTTTCAGTTTAGCCTTAACCGGCGCATGACTTCAAGACT
AACTCCTCTAAATCAATTACCAGTGGCTGCTGCCAGTGGTGCTTTTGCATGTCTTTCCGGGTTGGACTCAAGAC
GATAGTTACCGGATAAGGCGCAGCGGTCGGACTGAACGGGGGGTTCGTGCATACAGTCCAGCTTGGAGCGAA
CTGCCTACCCGGAACTGAGTGTCAGGCGTGGAATGAGACAAACGCGGCCATAACAGCGGAATGACACCGGTA
AACCGAAAGGCAGGAACAGGAGAGCGCACGAGGGAGCCGCCAGGGGGAAACGCCTGGTATCTTTATAGTCC
TGTCGGGTTTCGCCACCACTGATTTGAGCGTCAGATTTCGTGATGCTTGTCAGGGGGGCGGAGCCTATGGAAA
AACGGCTTTGCCGCGGCCCTCTCACTTCCCTGTTAAGTATCTTCCTGGCATCTTCCAGGAAATCTCCGCCCCGTT
CGTAAGCCATTTCCGCTCGCCGCAGTCGAACGACCGAGCGTAGCGAGTCAGTGAGCGAGGAAGCGGAATATA
TCCTGTATCACATATTCTGCTGACGCACCGGTGCAGCCTTTTTTCTCCTGCCACATGAAGCACTTCACTGACACC
CTCATCAGTGCCAACATAGTAAGCCAGTATACACTCCGCTAGCGCTGATGTCCGGCGGTGCTTTTGCCGTTACG
CACCACCCCGTCAGTAGCTGAACAGGAGGGACAGCTGATAGAAACAGAAGCCACTGGAGCACCTCAAAAACA
CCATCATACACTAAATCAGTAAGTTGGCAGCATCACCCGACGCACTTTGCGCCGAATAAATACCTGTGACGGAA
GATCACTTCGCAGAATAAATAAATCCTGGTGTCCCTGTTGATACCGGGAAGCCCTGGGCCAACTTTTGGCGAA
AATGAGACGTTGATCGGCACGTAAGAGGTTCCAACTTTCACCATAATGAAATAAGATCACTACCGGGCGTATT
TTTGAGTTATCGAGATTTTCAGGAGCTAAGGAAGCTAAAATGGAGAAAAAAATACACTGGATATACCACCGTT
GATATATCCCAATGGCATCGTAAAGAACATTTTGAGGCATTTCAGTCAGTTGCTCAATGTACCTATAACCAGAC
CGTTCAGCTGGATATTACGGCCTTTTTAAAGACCGTAAAGGAAAATAAGCACAAGTTTTATCCGGCCTTTATTC
ACATTCTTGCCCGCCTGATGAATGCTCATCCGGAATTCCGTATGGCAATGAAAGACGGTGAGCTGGTGATATG
GGATAGTGTTCACCCTTGTTACACCGTTTTCCATGAGCAAACTGAAACGTTTTCATCGCTCTGGAGTGAATACC
ACGACGATTTCCGGCAGTTTCTACACATATATTCGCAAGATGTGGCGTGTTACGGTGAAAACCTGGCCTATTTC
CCTAAAGGGTTTATTGAGAATATGTTTTTCGTCTCAGCCAATCCCTGGGTGAGTTTCACCAGTTTTGATTTAAAC
GTGGCCAATATGGACAACTTCTTCGCCCCCCCCGTTTTCACCATGGGCAAATATTATACGCAAGGCGACAAGGT
GCTGATGCCGCTGGCGATTCAGGTTCATCATGCCGTTTGTGATGGCTTCCATGTCGGCAGAATGCTTAATGAAT
TACAACAGTACTGCGATGAGTGGCAGGGCGGGGCGTAATTTTTTTAAGGCAGTTATTGGTGCCCTTAAACGCC
TGGTTGCTACGCCTGAATAAGTGATAATAAGCGGATGAATGGCAG

>Complete sequence of the pOHΔLacI plasmid. Features of the plasmid are shown in different colours: shown in orange, blue and green are genes *I-SceI, gam, Twin-Strep-tag – pduA, pduBJKNU* and *araBAD* promoter. Target sites for SphI, XhoI, SmaI and BglII restriction enzymes are shown in red.

GTGCACTCTCAGTACAATCTGCTCTGATGCCGCATAGTTAAGCCAGTATACACTCCGCTATCGCTACGTGACTG
GGTCATGGCTGCGCCCCGACACCCGCCAACACCCGCTGACGCGCCCTGACGGGCTTGTCTGCTCCCGGCATCC
GCTTACAGACAAGCTGTGACCGTCTCCGGGAGCTGCATGTGTCAGAGGTTTTCACCGTCATCACCGAAACGCG
CGAGGCAGCAAGGAGATGGCGCCCAACAGTCCCCCGGCCACGGGGCCTGCCACCATACCCACGCCGAAACAA
GCGCTCATGAGCCCGAAGTGGCGAGCCCGATCTTCCCCATCGGTGATGTCGGCGATATAGGCGCCAGCAACC
GCACCTGTGGCGCCGGTGATGCCGGCCACGATGCGTCCGGCGTAGAGGATCTGCTCATGTTTGACAGCTTATC
ATCGATGCATAATGTGCCTGTCAAATGGACGAAGCAGGGATTCTGCAAACCCTATGCTACTCCGTCAAGCCGT
CAATTGTCTGATTCGTTACCAATTATGACAACTTGACGGCTACATCATTCACTTTTTCTTCACAACCGGCACGGA
ACTCGCTCGGGCTGGCCCCGGTGCATTTTTTAAATACCCGCGAGAAATAGAGTTGATCGTCAAAACCAACATTG
CGACCGACGGTGGCGATAGGCATCCGGGTGGTGCTCAAAAGCAGCTTCGCCTGGCTGATACGTTGGTCCTCGC
GCCAGCTTAAGACGCTAATCCCTAACTGCTGGCGGAAAAGATGTGACAGACGCGACGGCGACAAGCAAACAT
GCTGTGCGACGCTGGCGATATCAAAATTGCTGTCTGCCAGGTGATCGCTGATGTACTGACAAGCCTCGCGTAC
CCGATTATCCATCGGTGGATGGAGCGACTCGTTAATCGCTTCCATGCGCCGCAGTAACAATTGCTCAAGCAGAT
TTATCGCCAGCAGCTCCGAATAGCGCCCTTCCCCTTGCCCGGCGTTAATGATTTGCCCAAACAGGTCGCTGAAA
TGCGGCTGGTGCGCTTCATCCGGGCGAAAGAACCCCGTATTGGCAAATATTGACGGCCAGTTAAGCCATTCAT
GCCAGTAGGCGCGCGGACGAAAGTAAACCCACTGGTGATACCATTCGCGAGCCTCCGGATGACGACCGTAGT
GATGAATCTCTCCTGGCGGGAACAGCAAAATATCACCCGGTCGGCAAACAAATTCTCGTCCCTGATTTTTCACC
ACCCCCTGACCGCGAATGGTGAGATTGAGAATATAACCTTTCATTCCCAGCGGTCGGTCGATAAAAAAATCGA
GATAACCGTTGGCCTCAATCGGCGTTAAACCCGCCACCAGATGGGCATTAAACGAGTATCCCGGCAGCAGGG
GATCATTTTGCGCTTCAGCCATACTTTTCATACTCCCGCCATTCAGAGAAGAAACCAATTGTCCATATTGCATCA
GACATTGCCGTCACTGCGTCTTTTACTGGCTCTTCTCGCTAACCAAACCGGTAACCCCGCTTATTAAAAGCATTC
TGTAACAAAGCGGGACCAAAGCCATGACAAAAACGCGTAACAAAAGTGTCTATAATCACGGCAGAAAAGTCC

araBAD promoter

ACATTGATTATTTGCACGGCGTCACACTTTGCTATGCCATAGCATTTTTATCCATAAGATTAGCGGATCCTACCT

GACGCTTTTTATCGCAACTCTCTACTGTTTCTCCATACCCGTTTTTTTGGGCTAGCAGGAGGGTACCTATATGCA

I-SceI

TATGAAAAACATCAAAAAAAACCAGGTAATGAACCTGGGTCCGAACTCTAAACTGCTGAAAGAATACAAATCC
CAGCTGATCGAACTGAACATCGAACAGTTCGAAGCAGGTATCGGTCTGATCCTGGGTGATGCTTACATCCGTT
CTCGTGATGAAGGTAAAACCTACTGTATGCAGTTCGAGTGGAAAAACAAAGCATACATGGACCACGTATGTCT
GCTGTACGATCAGTGGGTACTGTCCCCGCCGCACAAAAAAGAACGTGTTAACCACCTGGGTAACCTGGTAATC
ACCTGGGGCGCCCAGACTTTCAAACACCAAGCTTTCAACAAACTGGCTAACCTGTTCATCGTTAACAACAAAAA
AACCATCCCGAACAACCTGGTTGAAAACTACCTGACCCCGATGTCTCTGGCATACTGGTTCATGGATGATGGTG
GTAAATGGGATTACAACAAAAACTCTACCAACAAATCGATCGTACTGAACACCCAGTCTTTCACTTTCGAAGAA
GTAGAATACCTGGTTAAGGGTCTGCGTAACAAATTCCAACTGAACTGTTACGTAAAAATCAACAAAAACAAAC
CGATCATCTACATCGATTCTATGTCTTACCTGATCTTCTACAACCTGATCAAACCGTACCTGATCCCGCAGATGA

SphI

TGTACAAACTGCCGAACACTATCTCCTCCGAAACTTTCCTGAAATAAGGGCCCGACGTCTGCAGGCATGCTAAG

gam

GAGGTTATAAAAAATGGATATTAATACTGAAACTGAGATCAAGCAAAAGCATTCACTAACCCCCTTTCCTGTTT
TCCTAATCAGCCCGGCATTTCGCGGGCGATATTTTCACAGCTATTTCAGGAGTTCAGCCATGAACGCTTATTAC

ATTCAGGATCGTCTTGAGGCTCAGAGCTGGGCGCGTCACTACCAGCAGCTCGCCCGTGAAGAGAAAGAGGCA
GAACTGGCAGACGACATGGAAAAAGGCCTGCCCCAGCACCTGTTTGAATCGCTATGCATCGATCATTTGCAAC
GCCACGGGGCCAGCAAAAAATCCATTACCCGTGCGTTTGATGACGATGTTGAGTTTCAGGAGCGCATGGCAG
AACACATCCGGTACATGGTTGAAACCATTGCTCACCACCAGGTTGATATTGATTCAGAGGTATAAAATAATTTT

Twin-Strep-tag

GTTTAACTTTAAGAAGGAGATATACATATGTGGTCGCACCCGCAGTTCGAGAAAGGTGGAGGTTCCGGAGGT

XhoI

GGATCGGGAGGTTCGGCGTGGAGCCACCCGCAGTTCGAAAAAGCCTCGAGCCAACAAGAAGCGTTAGGAAT

pduA

GGTAGAAACCAAAGGCTTGACTGCAGCCATAGAGGCCGCAGATGCAATGGTGAAGTCAGCCAATGTAATGCT
GGTCGGCTACGAAAAAATTGGTTCGGGGCTGGTAACAGTCATTGTCCGCGGCGATGTTGGCGCAGTCAAAGC
AGCAACAGATGCAGGTGCCGCCGCAGCACGTAATGTGGGAGAAGTGAAAGCCGTACACGTCATCCCACGCCC
TCACACCGATGTAGAAAAAATCTTACCGAAGGGAATTAGCTAATGAGCAGCAATGAGCTGGTTGATCAGATCA

pduB

TGGCGCAGGTGATTGCTCGCGTGGCAACGCCGGAACAGCAGGCTATCCCTGAAAATAATCCTCCAACACGAGA
AACGGCTATGGCAGAGAAAAGCTGCAGTTTAACGGAGTTTGTCGGTACTGCGATTGGCGACACCGTCGGTCT
GGTAATCGCCAACGTGGACAGCGCCCTACTGGACGCAATGAAACTTGAAAAACGGTATCGCTCCATTGGCATC
CTTGGCGCGCGTACTGGTGCAGGCCCGCACATCATGGCCGCAGATGAAGCGGTAAAAGCCACCAATACTGAA
GTCGTCAGTATTGAGTTGCCACGTGATACCAAAGGCGGCGCGGGTCACGGTTCGCTGATTATTCTCGGCGGCA
ACGATGTTTCCGACGTGAAACGCGGAATTGAGGTTGCGCTGAAAGAACTGGATCGCACCTTTGGCGATGTGTA
TGCCAACGAAGCCGGTCACATCGAGATGCAATACACCGCACGCGCCAGCTACGCGCTGGAAAAAGCCTTTGGT
GCACCGATTGGCCGTGCCTGTGGCGTGATCGTCGGCGCGCCGGCATCCGTTGGTGTCCTGATGGCTGATACTG
CGCTGAAATCCGCCAACGTGGAAGTTGTGGCCTACAGCTCCCCTGCCCATGGCACCAGCTTCAGTAACGAAGC
CATTCTGGTCATTTCAGGCGATTCCGGCGCTGTGCGTCAGGCCGTTATCTCCGCCCGCGAAATCGGTAAAACCG
TACTCGGGACCCTCGGCTCAGAACCGAAAAACGATCGTCCGTCCTACATCTGAACTAGAAATAATTTTGTTTAA

pduJ

CTTTAAGAAGGAGATATACATATGAATAACGCACTGGGACTGGTTGAAACAAAAGGGCTCGTCGGCGCTATTG
AAGCCGCTGATGCCATGGTGAAATCCGCAAACGTGCAGTTGGTTGGTTACGAAAAAATCGGCTCAGGCCTTAT
CACCGTTATGGTTCGCGGCGATGTCGGCGCAGTGAAAGCTGCCGTAGATGCAGGAAGCGCTGCAGCAAGCGC
CGTTGGCGAGGTGAAATCCTGCCACGTTATCCCGCGTCCGCACAGCGACGTTGAAGCCATTTTACCTAAATCCG
CATAAATACTAGAAATAATTTTGTTTAACTTTAAGAAGGAGATATACATATGGTGAAGCAATCACTGGGATTAC

pduK

TTGAAGTTAGTGGTCTGGCATTAGCCATCAGTTGCGCGGACGTCATGGCGAAAGCCGCCTCCATCACGCTGGT
GGGCCTCGAAAAAACCAACGGTTCAGGCTGGATGGTGATCAAGATAATCGGGGATGTGGCCTCCGTCCAGGC
GGCCATTTCCACCGGTGTCAGTTTCGCTGACCAGCGAGATGGACTGGTGGCTCACAAAGTCATATCCAGACCA
GGGGATGGCATTCTGTCACATAGCGTCACCCCGGAGTCTGAGTCTGAGCCAGCGCCCGCCCCGACACCGGTCG
TACCACATGAAGAGATCCCTGAGGACCATGCAGCGCCCGAAGCGCCACAAGATGCAGAGTTGATTAGCTGCA
ATCTGTGTCTTGACCCTGCCTGCCCCCGTCAAAAGGGCGAGCCGCGCTCTCTTTGTCTGCACTCAGGCAAACGA
GGTGAAGCGTGAACTAGAAATAATTTTGTTTAACTTTAAGAAGGAGATATACATATGCATCTGGCACGGGTTA

pduN

CAGGCGTTGTGGTTTCCACGCAAAAATCTCCATCACTGGTGGGGAAGAAACTGTTGCTGGTACGTCGGGTGAG
TGCGGACGGAGAACTTCCCGCGTCCCCTGTGAGTGGAGATGAAGTCGCGGTTGATTCTGTCGGCGCTGGAACT

GGAGAACTGGTATTACTCAGCAGTGGCTCCAGCGCCAGACACGTTTTTTCCGGCCCTAATGAGGCCATCGATCT
GGCTATCGTCGGCATTGTCGACACGCTTTCTCGTTAGACTAGAAATAATTTTGTTTAACTTTAAGAAGGAGATA

pduU

TACATATGGAAAGACAACCCACCACGGATCGTATGATTCAGGAATATGTTCCTGGCAAGCAGGTTACGCTGGC
GCATCTTATCGCTAATCCAGGTAAAGATTTGTTCAAGAAACTGGGATTACCAGAGTCGGTTTCCGCAATCGGTA
TTCTGACGATTACCCCCAGCGAAGCCTCAATCATCGCCTGTGATATCGCCACGAAATCCGGGGCGGTAGAGAT
TGGTTTTCTCGACCGTTTTACCGGCGCAGTGGTACTGACAGGCGATGTTTCCGCTGTTGAGTACGCGCTGAAAC

BglII SmaI

AGGTAACCCGGACGCTGGGCGAAATGATGCGTTTTACCGCCTGTCCCATCACCCGGACATAAAGATCTCCCGG
GGGCTGTTTTGGCGGATGAGAGAAGATTTTCAGCCTGATACAGATTAAATCAGAACGCAGAAGCGGTCTGAT
AAAACAGAATTTGCCTGGCGGCAGTAGCGCGGTGGTCCCACCTGACCCCATGCCGAACTCAGAAGTGAAACG
CCGTAGCGCCGATGGTAGTGTGGGGTCTCCCCATGCGAGAGTAGGGAACTGCCAGGCATCAAATAAAACGAA
AGGCTCAGTCGAAAGACTGGGCCTTTCGTTTTATCTGTTGTTTGTCGGTGAACGCTCTCCTGAGTAGGACAAAT
CCGCCGGGAGCGGATTTGAACGTTGCGAAGCAACGGCCCGGAGGGTGGCGGGCAGGACGCCCGCCATAAAC
TGCCAGGCATCAAATTAAGCAGAAGGCCATCCTGACGGATGGCCTTTTTGCGTTTCTACAAACTCTTTTGTTTAT
TTTTCTAAATACATTCAAATATGTATCCGCTCATGAGACAATAACCCTGATAAATGCTTCAATAATATTGAAAAA
GGAAGAGTATGAGTATTCAACATTTCCGTGTCGCCCTTATTCCCTTTTTTGCGGCATTTTGCCTTCCTGTTTTTGC
TCACCCAGAAACGCTGGTGAAAGTAAAAGATGCTGAAGATCAGTTGGGTGCACCGATGATAAGCTGTCAAAC
ATGAGAATTACAACTTATATCGTATGGGGCTGACTTCAGGTGCTACATTTGAAGAGATAAATTGCACTGAAATC
TAGAAATATTTTATCTGATTAATAAGATGATCTTCTTGAGATCGTTTTGGTCTGCGCGTAATCTCTTGCTCTGAA
AACGAAAAAACCGCCTTGCAGGGCGGTTTTTCGAAGGTTCTCTGAGCTACCAACTCTTTGAACCGAGGTAACT
GGCTTGGAGGAGCGCAGTCACCAAAACTTGTCCTTTCAGTTTAGCCTTAACCGGCGCATGACTTCAAGACTAAC
TCCTCTAAATCAATTACCAGTGGCTGCTGCCAGTGGTGCTTTTGCATGTCTTTCCGGGTTGGACTCAAGACGAT
AGTTACCGGATAAGGCGCAGCGGTCGGACTGAACGGGGGGTTCGTGCATACAGTCCAGCTTGGAGCGAACTG
CCTACCCGGAACTGAGTGTCAGGCGTGGAATGAGACAAACGCGGCCATAACAGCGGAATGACACCGGTAAAC
CGAAAGGCAGGAACAGGAGAGCGCACGAGGGAGCCGCCAGGGGGAAACGCCTGGTATCTTTATAGTCCTGT
CGGGTTTCGCCACCACTGATTTGAGCGTCAGATTTCGTGATGCTTGTCAGGGGGGCGGAGCCTATGGAAAAAC
GGCTTTGCCGCGGCCCTCTCACTTCCCTGTTAAGTATCTTCCTGGCATCTTCCAGGAAATCTCCGCCCCGTTCGT
AAGCCATTTCCGCTCGCCGCAGTCGAACGACCGAGCGTAGCGAGTCAGTGAGCGAGGAAGCGGAATATATCC
TGTATCACATATTCTGCTGACGCACCGGTGCAGCCTTTTTTCTCCTGCCACATGAAGCACTTCACTGACACCCTC
ATCAGTGCCAACATAGTAAGCCAGTATACACTCCGCTAGCGCTGATGTCCGGCGGTGCTTTTGCCGTTACGCAC
CACCCCGTCAGTAGCTGAACAGGAGGGACAGCTGATAGAAACAGAAGCCACTGGAGCACCTCAAAAACACCA
TCATACACTAAATCAGTAAGTTGGCAGCATCACCCGACGCACTTTGCGCCGAATAAATACCTGTGACGGAAGA
TCACTTCGCAGAATAAATAAATCCTGGTGTCCCTGTTGATACCGGGAAGCCCTGGGCCAACTTTTGGCGAAAAT
GAGACGTTGATCGGCACGTAAGAGGTTCCAACTTTCACCATAATGAAATAAGATCACTACCGGGCGTATTTTTG
AGTTATCGAGATTTTCAGGAGCTAAGGAAGCTAAAATGGAGAAAAAAATCACTGGATATACCACCGTTGATA
TATCCCAATGGCATCGTAAAGAACATTTTGAGGCATTTCAGTCAGTTGCTCAATGTACCTATAACCAGACCGTT
CAGCTGGATATTACGGCCTTTTTAAAGACCGTAAAGGAAAATAAGCACAAGTTTTATCCGGCCTTTATTCACAT
TCTTGCCCGCCTGATGAATGCTCATCCGGAATTCCGTATGGCAATGAAAGACGGTGAGCTGGTGATATGGGAT
AGTGTTCACCCTTGTTACACCGTTTTCCATGAGCAAACTGAAACGTTTTCATCGCTCTGGAGTGAATACCACGAC
GATTTCCGGCAGTTTCTACACATATATTCGCAAGATGTGGCGTGTTACGGTGAAAACCTGGCCTATTTCCCTAA
AGGGTTTATTGAGAATATGTTTTTCGTCTCAGCCAATCCCTGGGTGAGTTTCACCAGTTTTGATTTAAACGTGGC
CAATATGGACAACTTCTTCGCCCCCCCCGTTTTCACCATGGGCAAATATTATACGCAAGGCGACAAGGTGCTGA
TGCCGCTGGCGATTCAGGTTCATCATGCCGTTTGTGATGGCTTCCATGTCGGCAGAATGCTTAATGAATTACAA
CAGTACTGCGATGAGTGGCAGGGCGGGGCGTAATTTTTTTAAGGCAGTTATTGGTGCCCTTAAACGCCTGGTT
GCTACGCCTGAATAAGTGATAATAAGCGGATGAATGGCAG

>Complete sequence of the pRW-(*lacO*)$_5$ plasmid. Underlined and in red are the target sites for the EcoRI and HindIII restriction enzymes. Recognition sites for the yeast meganuclease I-SceI are shown in orange. Also marked are binding sites for the LacI repressor (blue) and LexA repressor (green). The promoter region of the *cka* gene is presented in purple.

I-SceI
GAATTGTAGGGATAACAGGGTAATGAATTGAGCCAGCACGTAGCTAGCAGAGTAGTAAGGAATTGT
LacI LacI LacI
GAGCGGATAACAATTAAAAGTCAGAAATTGTGAGCGGATAACAATTAAAGACGGAGAATTGTGAGC
LacI
GGATAACAATTAATAAAGCAGCTCTAGCATCAACGCGCAAATTGTGAGCGGATAACAATTAGCAAGA
LacI EcoRI *cka* promoter
CAGAATTGTGAGCGGATAACAATTCTGGGAATTCCTTTGTCATGCTCATATCCGGCAGCAACAAGGGTGTCGA
GAAGTCGTTTGTCTGTTTTGTTCAGTTTATGGCGGGGTGATTTGCTCACATCGTTCTCCGTTTTTCATGATCCA
ACAGTTTTATATCTCATCCTGATTTGCTCCGGTGTTCTGTTTCGACGTTCTCAATGCC
-35
TGCCTCCCCATAGTGTGATCCTTATTGCAAAAATGAAAATCCATGCTC**TTGACA**TGGACAATGCTGAG
-10 LexA LexA
TAG**TAGGTT**TTTACTGTACATAAAACCAGTGGTTATATGTACAGTATATTGTTTTTAATTTATTGTTTT
cka HindIII I-SceI
AATTATCAAAGAGGAATTTATGGCTAAAGAACAAGCTTTAGGGATAACAGGGTAATTTGTCAGTGCGCAAAAA
GATCCTGAATTTCAGGCTCAGTTCAACGACCTGCTGAAAAACTATGCCGGGCGTCCAACCGCGCTGACCAAAT
GCCAGAACATTACAGCCGGGACGAACACCACGCTGTATCTCAAGCGTGAAGATTTGCTGCACGGCGGCGCGC
ATAAAACTAACCAGGTGCTGGGGCAGGCGTTGCTGGCGAAGCGGATGGGTAAAACCGAAATCATCGCCGAAA
CCGGTGCCGGTCAGCATGGCGTGGCGTCGGCCCTGGCCAGCGCCCTGCTCGGCCTGAAATGCCGTATTTATAT
GGGTGCCAAAGACGTTGAACGCCAGTCGCCTAACGTTTTTCGTATGCGCTTAATGGGTGCGGAAGTGATCCCG
GTGCATAGCGGTTCCGCGACGCTGAAAGATGCCTGTAACGAGGCGCTGCGCGACTGGTCCGGTAGTTACGAA
ACCGCGCACTATATGCTGGGCACCGCAGCTGGCCCGCATCCTTATCCGACCATTGTGCGTGAGTTTCAGCGGAT
GATTGGCGAAGAAACCAAAGCGCAGATTCTGGAAAGAGAAGGTCGCCTGCCGGATGCCGTTATCGCCTGTGT
TGGCGGCGGTTCGAATGCCATCGGCATGTTTGCTGATTTCATCAATGAAACCAACGTCGGCCTGATTGGTGTG
GAGCCAGGTGGTCACGGTATCGAAACTGGCGAGCACGGCGCACCGCTAAAACATGGTCGCGTGGGTATCTAT
TTCGGTATGAAAGCGCCGATGATGCAAACCGAAGACGGGCAGATTGAAGAATCTTACTCCATCTCCGCCGGAC
TGGATTTCCCGTCTGTCGGCCCACAACACGCGTATCTTAACAGCACTGGACGCGCTGATTACGTGTCTATTACC
GATGATGAAGCCCTTGAAGCCTTCAAAACGCTGTGCCTGCACGAAGGGATCATCCCGGCGCTGGAATCCTCCC
ACGCCTTGGCCCATGCGTTGAAAATGATGCGCGAAAACCCGGATAAAGAGCAGCTACTGGTGGTTAACCTTTC
CGGTCGCGGCGATAAAGACATCTTCACCGTTCACGATATTTTGAAAGCACGAGGGGAAATCTGATGGAACGCT
ACGAATCTCTGTTTGCCCAGTTGAAGGAGCGCAAAGAAGGCGCATTCGTTCCTTTCGTCACGCTCGGTGATCCG
GGCATTGAGCAGTCATTGAAAATTATCGATACGCTAATTGAAGCCGGTGCTGACGCGCTGGAGTTAGGTATCC
CCTTCTCCGACCCACTGGCGATTACGGATTCACTGGCCGTCGTTTTACAACGTCGTGACTGGGAAAACCCTGGC
GTTACCCAACTTAATCGCCTTGCAGCACATCCCCCTTTCGCCAGCTGGCGTAATAGCGAAGAGGCCCGCACCGA
TCGCCCTTCCCAACAGTTGCGCAGCCTGAATGGCGAATGGCGCTTTGCCTGGTTTCCGGCACCAGAAGCGGTG
CCGGAAAGCTGGCTGGAGTGCGATCTTCCTGAGGCCGATACTGTCGTCGTCCCCTCAAACTGGCAGATGCACG
GTTACGATGCGCCCATCTACACCAACGTAACCTATCCCATTACGGTCAATCCGCCGTTTGTTCCCACGGAGAAT
CCGACGGGTTGTTACTCGCTCACATTTAATGTTGATGAAAGCTGGCTACAGGAAGGCCAGACGCGAATTATTTT
TGATGGCGTTAACTCGGCGTTTCATCTGTGGTGCAACGGGCGCTGGGTCGGTTACGGCCAGGACAGTCGTTTG
CCGTCTGAATTTGACCTGAGCGCATTTTTACGCGCCGGAGAAAACCGCCTCGCGGTGATGGTGCTGCGTTGGA
GTGACGGCAGTTATCTGGAAGATCAGGATATGTGGCGGATGAGCGGCATTTTCCGTGACGTCTCGTTGCTGCA
TAAACCGACTACACAAATCAGCGATTTCCATGTTGCCACTCGCTTTAATGATGATTTCAGCCGCGCTGTACTGG
AGGCTGAAGTTCAGATGTGCGGCGAGTTGCGTGACTACCTACGGGTAACAGTTTCTTTATGGCAGGGTGAAAC
GCAGGTCGCCAGCGGCACCGCGCCTTTCGGCGGTGAAATTATCGATGAGCGTGGTGGTTATGCCGATCGCGTC

ACACTACGTCTGAACGTCGAAAACCCGAAACTGTGGAGCGCCGAAATCCCGAATCTCTATCGTGCGGTGGTTG
AACTGCACACCGCCGACGGCACGCTGATTGAAGCAGAAGCCTGCGATGTCGGTTTCCGCGAGGTGCGGATTG
AAAATGGTCTGCTGCTGCTGAACGGCAAGCCGTTGCTGATTCGAGGCGTTAACCGTCACGAGCATCATCCTCT
GCATGGTCAGGTCATGGATGAGCAGACGATGGTGCAGGATATCCTGCTGATGAAGCAGAACAACTTTAACGC
CGTGCGCTGTTCGCATTATCCGAACCATCCGCTGTGGTACACGCTGTGCGACCGCTACGGCCTGTATGTGGTG
GATGAAGCCAATATTGAAACCCACGGCATGGTGCCAATGAATCGTCTGACCGATGATCCGCGCTGGCTACCGG
CGATGAGCGAACGCGTAACGCGAATGGTGCAGCGCGATCGTAATCACCCGAGTGTGATCATCTGGTCGCTGG
GGAATGAATCAGGCCACGGCGCTAATCACGACGCGCTGTATCGCTGGATCAAATCTGTCGATCCTTCCCGCCC
GGTGCAGTATGAAGGCGGCGGAGCCGACACCACGGCCACCGATATTATTTGCCCGATGTACGCGCGCGTGGA
TGAAGACCAGCCCTTCCCGGCTGTGCCGAAATGGTCCATCAAAAAATGGCTTTCGCTACCTGGAGAGACGCGC
CCGCTGATCCTTTGCGAATACGCCCACGCGATGGGTAACAGTCTTGGCGGTTTCGCTAAATACTGGCAGGCGT
TTCGTCAGTATCCCCGTTTACAGGGCGGCTTCGTCTGGGACTGGGTGGATCAGTCGCTGATTAAATATGATGA
AAACGGCAACCCGTGGTCGGCTTACGGCGGTGATTTTGGCGATACGCCGAACGATCGCCAGTTCTGTATGAAC
GGTCTGGTCTTTGCCGACCGCACGCCGCATCCAGCGCTGACGGAAGCAAAACACCAGCAGCAGTTTTTCCAGT
TCCGTTTATCCGGGCAAACCATCGAAGTGACCAGCGAATACCTGTTCCGTCATAGCGATAACGAGCTCCTGCAC
TGGATGGTGGCGCTGGATGGTAAGCCGCTGGCAAGCGGTGAAGTGCCTCTGGATGTCGCTCCACAAGGTAAA
CAGTTGATTGAACTGCCTGAACTACCGCAGCCGGAGAGCGCCGGGCAACTCTGGCTCACAGTACGCGTAGTGC
AACCGAACGCGACCGCATGGTCAGAAGCCGGGCACATCAGCGCCTGGCAGCAGTGGCGTCTGGCGGAAAACC
TCAGTGTGACGCTCCCCGCCGCGTCCCACGCCATCCCGCATCTGACCACCAGCGAAATGGATTTTTGCATCGAG
CTGGGTAATAAGCGTTGGCAATTTAACCGCCAGTCAGGCTTTCTTTCACAGATGTGGATTGGCGATAAAAAAC
AACTGCTGACGCCGCTGCGCGATCAGTTCACCCGTGCACCGCTGGATAACGACATTGGCGTAAGTGAAGCGAC
CCGCATTGACCCTAACGCCTGGGTCGAACGCTGGAAGGCGGCGGGCCATTACCAGGCCGAAGCAGCGTTGTT
GCAGTGCACGGCAGATACACTTGCTGATGCGGTGCTGATTACGACCGCTCACGCGTGGCAGCATCAGGGGAA
AACCTTATTTATCAGCCGGAAAACCTACCGGATTGATGGTAGTGGTCAAATGGCGATTACCGTTGATGTTGAA
GTGGCGAGCGATACACCGCATCCGGCGCGGATTGGCCTGAACTGCCAGCTGGCGCAGGTAGCAGAGCGGGT
AAACTGGCTCGGATTAGGGCCGCAAGAAAACTATCCCGACCGCCTTACTGCCGCCTGTTTTGACCGCTGGGAT
CTGCCATTGTCAGACATGTATACCCCGTACGTCTTCCCGAGCGAAAACGGTCTGCGCTGCGGGACGCGCGAAT
TGAATTATGGCCCACACCAGTGGCGCGGCGACTTCCAGTTCAACATCAGCCGCTACAGTCAACAGCAACTGAT
GGAAACCAGCCATCGCCATCTGCTGCACGCGGAAGAAGGCACATGGCTGAATATCGACGGTTTCCATATGGG
GATTGGTGGCGACGACTCCTGGAGCCCGTCAGTATCGGCGGAACTCCAGCTGAGCGCCGGTCGCTACCATTAC
CAGTTGGTCTGGTGTCAAAAATAATAATAACCGGGCAGGCCATGTCTGCCCGTATTTCGCGTAAGGAAATCCA
TTATGTACTATTTAAAAAACACAAACTTTTGGATGTTCGGTTTATTCTTTTTCTTTTACTTTTTTATCATGGGAGCC
TACTTCCCGTTTTTCCCGATTTGGCTACATGACATCAACCATATCAGCAAAAGTGATACGGGTATTATTTTTGCC
GCTATTTCTCTGTTCTCGCTATTATTCCAACCGCTGTTTGGTCTGCTTTCTGACAAACTCGGGCTGCGCAAATAC
CTGCTGTGGATTATTACCGGCATGTTAGTGATGTTTGCGCCGTTCTTTATTTTTATCTTCGGGCCACTGTTACAA
TACAACATTTTAGTAGGATCGATTGTTGGTGGTATTTATCTAGGCTTTTGTTTTAACGCCGGTGCGCCAGCAGT
AGAGGCATTTATTGAGAAAGTCAGCCGTCGCAGTAATTTCGAATTTGGTCGCGCGCGGATGTTTGGCTGTGTT
GGCTGGGCGCTGTGTGCCTCGATTGTCGGCATCATGTTCACCATCAATAATCAGTTTGTTTTCTGGCTGGGCTC
TGGCTGTGCACTCATCCTCGCCGTTTTACTCTTTTTCGCCAAAACGGATGCGCCCTCTTCTGCCACGGTTGCCAA
TGCGGTAGGTGCCAACCATTCGGCATTTAGCCTTAAGCTGGCACTGGAACTGTTCAGACAGCCAAAACTGTGG
TTTTTGTCACTGTATGTTATTGGCGTTTCCTGCACCTACGATGTTTTTGACCAACAGTTTGCTAATTTCTTTACTTC
GTTCTTTGCTACCGGTGAACAGGGTACGCGGGTATTTGGCTACGTAACGACAATGGGCGAATTACTTAACGCC
TCGATTATGTTCTTTGCGCCACTGATCATTAATCGCATCGGTGGGAAAAACGCCCTGCTGCTGGCTGGCACTAT
TATGTCTGTACGTATTATTGGCTCATCGTTCGCCACCTCAGCGCTGGAAGTGGTTATTCTGAAAACGCTGCATAT
GTTTGAAGTACCGTTCCTGCTGGTGGGCTGCTTTAAATATATTACCAGCCAGTTTGAAGTGCGTTTTTCAGCGA
CGATTTATCTGGTCTGTTTCTGCTTCTTTAAGCAACTGGCGATGATTTTTATGTCTGTACTGGCGGGCAATATGT
ATGAAAGCATCGGTTTCCAGGGCGCTTATCTGGTGCTGGGTCTGGTGGCGCTGGGCTTCACCTTAATTTCCGTG
TTCACGCTTAGCGGCCCCGGCCCGCTTTCCCTGCTGCGTCGTCAGGTGAATGAAGTCGCTTAAGCAATCAATGT
CGGATGCGGCGCGACGCTTATCCGACCAACATATCATAACGGAGTGATCGCATTGAACATGCCAATGACCGAA
AGAATAAGAGCAGGCAAGCTATTTACCGATATGTGCGAAGGCTTACCGGAAAAAAGACTTCGTGGGAAAACG
TTAATGTATGAGTTTAATCACTCGCATCCATCAGAAGTTGAAAAAAGAGAAAGCCTGATTAAAGAAATGTTTGC
CACGGTAGGGGAAAACGCCTGGGTAGAACCGCCTGTCTATTTCTCTTACGGTTCCAACATCCATATAGGCCGC

AATTTTTATGCAAATTTCAATTTAACCATTGTCGATGACTACACGGTAACAATCGGTGATAACGTACTGATTGCA
CCCAACGTTACTCTTTCCGTTACGGGACACCCTGTACACCATGAATTGAGAAAAAACGGCGAGATGTACTCTTT
TCCGATAACGATTGGCAATAACGTCTGGATCGGAAGTCATGTGGTTATTAATCCAGGCGTCACCATCGGGGAT
AATTCTGTTATTGGCGCGGGTAGTATCGTCACAAAAGACATTCCACCAAACGTCGTGGCGGCTGGCGTTCCTTG
TCGGGTTATTCGCGAAATAAACGACCGGGATAAGCACTATTATTTCAAAGATTATAAAGTTGAATCGTCAGTTT
AAATTATAAAAATTGCCTGATACGCTGCGCTTATCAGGCCTACAAGTTCAGCGATCTACATTAGCCGCATCCGG
CATGAACAAAGCGCAGGAACAAGCGTCGCATCATGCCTCTTTGACCCACAGCTGCGGAAAACGTACTGGTGCA
AAACGCAGGGTTATGATCATCAGCCCAACGACGCACAGCGCATGAAATGCCCAGTCCATCAGGTAATTGCCGC
TGATACTACGCAGCACGCCAGAAAACCACGGGGCAAGCCCGGCGATGATAAAACCGATTCCCTGCATAAACG
CCACCAGCTTGCCAGCAATAGCCGGTTGCACAGAGTGATCGAGCGCCAGCAGCAAACAGAGCGGAAACGCGC
CGCCCAGACCTAACCCACACACCATCGCCCACAATACCGGCAATTGCATCGGCAGCCAGATAAAGCCGCAGAA
CCCCACCAGTTGTAACACCAGCGCCAGCATTAACAGTTTGCGCCGATCCTGATGGCGAGCCATAGCAGGCATC
AGCAAAGCTCCTGCGGCTTGCCCAAGCGTCATCAATGCCAGTAAGGAACCGCTGTACTGCGCGCTGGCACCAA
TCTCAATATAGAAAGCGGGTTACCGCCAAGCTAGCTTTTGCCATTCTCACCGGATTCAGTCGTCACTCATGGTG
ATTTCTCACTTGATAACCTTATTTTTGACGAGGGGAAATTAATAGGTTGTATTGATGTTGGACGAGTCGGAATC
GCAGACCGATACCAGGATCTTGCCATCCTATGGAACTGCCTCGGTGAGTTTTCTCCTTCATTACAGAAACGGCT
TTTTCAAAAATATGGTATTGATAATCCTGATATGAATAAATTGCAGTTTCATTTGATGCTGGATGAGTTTTTCTA
ATCAGAATTGGTTAATTGGTTGTAACACTGGCAGAGCATTACGCTGACTTGACGGGACGGCGGCTTTGTTGAA
TAAATCGAACTTTTGCTGAGTTGAAGGATCAGATCACGCATCTTCCCGACAACGCAGACCGTTCCGTGGCAAA
GCAAAAGTTCAAAATCACCAACTGGTCCACCTACAACAAAGCTCTCATCAACCGTGGCTCCCTCACTTTCTGGCT
GGATGATGGGGCGATTCAGGCCTGGTATGAGTCAGCAACACCTTCTTCACGAGGCAGACCTCAGCGCCAGAA
GGCCGCCAGAGAGGCCGAGCGCGGCGTGAGGCTTGGACGCTAGGGCAGGGCATGAAAAAGCCCGTAGCGG
GCGCTACGGGGCTCTGACGCGGTGGAAAGGGGGAGGGGATGTGTTGTCTACATGGCTCTGCTGTAGTGAGTG
GGTTGCGCTCCGGCAGCGGTCCTGATCAATCGTCACCCTTTCTCGGTCCTTCAACGTTCCTGACAACGAGCCTC
CTTTTCGCCAATCCATCGACAATCACCGCGAGTCCCTGCTCGAACGCTGCGTCCGGACCGGCTTCGTCGAAGGC
GTCTATCGCGGCCCGCAACAGCGGCGGAGAGCGGAGCCTGTTCAACGGTGCCGCCGCGCTCGCCGGACTCGC
TGTCGCCGGCCTGCTCCTCAAGCACGGCCCCAACAGTGAAGTAGCTGATTGTCATCAGCGCATTGACGGCGTC
CCCGGCCGAAAAACCCGCCTCGCAGAGGAAGCGAAGCTGCGCGTCGGCCGTTTCCATCTGCGGTGCGCCCGG
TCGCGTGCCGGCATGGATGCGCGCGCCATCGCGGTAGGCGAGCAGCGCCTGCCTGAAGCTGCGGGCATTCCC
AGTCAGAAATGAGCGCCAGTCGTCGTCGGCTCTCGGCACCGAAGTGCTATGATTCTCCGCCAGCATGGCTTCG
GCCAGTGCGTCGAGCAGCGCCCGCTTGTTCCTGAAGTGCCAGTAAAGCGCCGGCTGCTGAACCCCCAACCGTT
CCGCCAGTTTGCGTGTCGTCAGACCGTCTACGCCGACCTCGTTCAACAGGTCTAGGGCGGCACGGATCACTGT
ATTCGGCTGCAACTTTGTCATGCTTGACACTTTATCACTGATAAACATAATATGTCCACCAACTTATCAGTGATA
AAGAATCCGCGCGTTCAATCGGACCAGCGGAGGCTGGTCCGGAGGCCAGACGTGAAACCCAACATACCCCTG
ATCGTAATTCTGAGCACTGTCGCGCTCGACGCTGTCGGCATCGGCCTGATTATGCCGGTGCTGCCGGGCCTCCT
GCGCGATCTGGTTCACTCGAACGACGTCACCGCCCACTATGGCATTCTGCTGGCGCTGTATGCGTTGGTGCAAT
TTGCCTGCGCACCTGTGCTGGGCGCGCTGTCGGATCGTTTCGGGCGGCGGCCAATCTTGCTCGTCTCGCTGGC
CGGCGCCACTGTCGACTACGCCATCATGGCGACAGCGCCTTTCCTTTGGGTTCTCTATATCGGGCGGATCGTGG
CCGGCATCACCGGGGCGACTGGGGCGGTAGCCGGCGCTTATATTGCCGATATCACTGATGGCGATGAGCGCG
CGCGGCACTTCGGCTTCATGAGCGCCTGTTTCGGGTTCGGGATGGTCGCGGGACCTGTGCTCGGTGGGCTGAT
GGGCGGTTTCTCCCCCCACGCTCCGTTCTTCGCCGCGGCAGCCTTGAACGGCCTCAATTTCCTGACGGGCTGTT
TCCTTTTGCCGGAGTCGCACAAAGGCGAACGCCGGCCGTTACGCCGGGAGGCTCTCAACCCGCTCAGCTTCGT
TCGGTGGGCCCGGGGCATGACCGTCGTCGCCGCCCTGATGGCGGTCTTCTTCATCATGCAACTTGTCGGACAG
GTGCCGGCCGCGCTTTGGGTCATTTTCGGCGAGGATCGCTTTCACTGGGACGCGACCACGATCGGCATTTCGC
TTGCCGCATTTGGCATTCTGCATTCACTCGCCCAGGCAATGATCACCGGCCCTGTAGCCGCCCGGCTCGGCGAA
AGGCGGGCACTCATGCTCGGAATGATTGCCGACGGCACAGGCTACATCCTGCTTGCCTTCGCGACACGGGGAT
GGATGGCGTTCCCGATCATGGTCCTGCTTGCTTCGGGTGGCAGCGGAATGCCGGCGCTGCAAGCAATGTTGTC
CAGGCAGGTGGATGAGGAACGCCAGGGGCAGCTGCAAGGCTCACTGGCGGCGCTCACCAGCCTGACCTCGAT
CGTCGGACCCCTCCTCTTCACGGCGATCTATGCGGCTTCTATAACAACGTGGAACGGGTGGGCATGGATTGCA
GGCGCTGCCCTCTACTTGCTCTGCCTGCCGGCGCTGCGTCGCGGGCTTTGGAGCGGCGCAGGGCAACGAGCC
GATCGCTGATCGTGGAAACGATAGGCCTATGCCATGCGGGTCAAGGCGACTTCCGGCAAGCTATACGCGCCCT
AGGAGTGCGGTTGGAACGTTGGCCCAGCCAGACACTCCCGATCACGAGCAGGACGCCGATGATTTGAAGCGC

ACTCAGCGTCTGATCCAAGAACAACCATCCTAGCAACAGCGCGGCGACGGCGGTCCCCGGGCTGAGAAAGCC
CAGTAAGGAAACAACTGTAGGTTCGAGTCGCGAGATCCCCCGGAACCAAAGGAAGTATGGTTAAACCCGCTC
CGATCAGGCCGAGCCACGCCAGGCCGAGAACATTGGTTCCTGTAGGCATCGGGATTGGCGGATCAAACACTA
AAGCTACTGGAACGAGCAGAAGTCCTCCGGCCGCCAGTTGCAGGTAAAGGTGAGCAGAGGCACGGGAGGTT
GCCACTGCGGGTCAGCACGGTTCCGAACGCCATGGGAAACCGCCCCCGCAGGCCCGCTGCGACGCCGACAGG
ATCTAGCGCTGCGTTTGGTGTCAACACCAACAGCGCCACGCCCGCAGTTCCGCAGATAGCCCCAGGACCGCCA
TCAATCGTATCGGGCTACCTAGCAGAGCGGCAGAGATGAACACGACCATCAGCGGCTGCACACGCCTACCGTC
GCCGCGACCCGCCCGGCAGGCGGTAGACCGAAATAAACAACAAGCTCCAGAATAGCGAAATATTAAGTGCGC
CGAGGATGAAGATGCGCATCCACCAGATTCCCGTTGGAATCTGTCGGACGATCATCACGAGCAATAAACCCGC
CGGCAACGCCCGGAGCAGCATACCGGCGACCCCTCGGCCTCGCTGTTCGGGCTCCACGAAGACGCCGGACAT
ATGCGCCTTGTGAGCGTCCTTGGGGCCGTCCTCCTGTTTCAAGACCGACAGCCCAATGATCTCGCCGTCGATGT
AGGCGCCGAATGCCACGGCATCTCGCAACCGTTCAGCGAACGCCTCCATGGGCTTTTTCTCCTCGTGCTCGTAA
ACGGACCCGAACATCTCTGGAGCTTTCTTCAGGGCCGACAATCGGATCTCGCGGAAATCCTGCACGTCGGCCG
CTCCAAGCCGTCGAATCTGAGCCTTAATCACAATTGTCAATTTTAATCCTCTGTTTATCGGCAGTTCGTAGAGCG
CGCCGTGCGTCCCGAGCGATACTGAGCGAAGCAAGTGCGTCGAGCAGTGCCCGCTTGTTCCTGAAATGCCAGT
AAAGCGCTGGCTGCTGAACCCCCAGCCGGAACTGACCCCACAAGGCCCTAGCGTTTGCAATGCACCAGGTCAT
CATTGACCCAGGCGTGTTCCACCAGGCCGCTGCCTCGCAACTCTTCGCAGGCTTCGCCGACCTGCTCGCGCCAC
TTCTTCACGCGGGTGGAATCCGATCCGCACATGAGGCGGAAGGTTTCCAGCTTGAGCGGGTACGGCTCCCGGT
GCGAGCTGAAATAGTCGAACATCCGTCGGGCCGTCGGCGACAGCTTGCGGTACTTCTCCCATATGAATTTCGT
GTAGTGGTCGCCAGCAAACAGCACGACGATTTCCTCGTCGATCAGGACCTGGCAACGGGACGTTTTCTTGCCA
CGGTCCAGGACGCGGAAGCGGTGCAGCAGCGACACCGATTCCAGGTGCCCAACGCGGTCGGACGTGAAGCC
CATCGCCGTCGCCTGTAGGCGCGACAGGCATTCCTCGGCCTTCGTGTAATACCGGCCATTGATCGACCAGCCCA
GGTCCTGGCAAAGCTCGTAGAACGTGAAGGTGATCGGCTCGCCGATAGGGGTGCGCTTCGCGTACTCCAACA
CCTGCTGCCACACCAGTTCGTCATCGTCGGCCCGCAGCTCGACGCCGGTGTAGGTGATCTTCACGTCCTTGTTG
ACGTGGAAAATGACCTTGTTTTGCAGCGCCTCGCGCGGGATTTTCTTGTTGCGCGTGGTGAACAGGGCAGAGC
GGGCCGTGTCGTTTGGCATCGCTCGCATCGTGTCCGGCCACGGCGCAATATCGAACAAGGAAAGCTGCATTTC
CTTGATCTGCTGCTTCGTGTGTTTCAGCAACGCGGCCTGCTTGGCCTCGCTGACCTGTTTTGCCAGGTCCTCGCC
GGCGGTTTTTCGCTTCTTGGTCGTCATAGTTCCTCGCGTGTCGATGGTCATCGACTTCGCCAAACCTGCCGCCTC
CTGTTCGAGACGACGCGAACGCTCCACGGCGGCCGATGGCGCGGGCAGGGCAGGGGGAGCCAGTTGCACGC
TGTCGCGCTCGATCTTGGCCGTAGCTTGCTGGACCATCGAGCCGACGGACTGGAAGGTTTCGCGGGGCGCAC
GCATGACGGTGCGGCTTGCGATGGTTTCGGCATCCTCGGCGGAAAACCCCGCGTCGATCAGTTCTTGCCTGTA
TGCCTTCCGGTCAAACGTCCGATTCATTCACCCTCCTTGCGGGATTGCCCCGACTCACGCCGGGGCAATGTGCC
CTTATTCCTGATTTGACCCGCCTGGTGCCTTGGTGTCCAGATAATCCACCTTATCGGCAATGAAGTCGGTCCCGT
AGACCGTCTGGCCGTCCTTCTCGTACTTGGTATTCCGAATCTTGCCCTGCACGAATACCAGCGACCCCTTGCCCA
AATACTTGCCGTGGGCCTCGGCCTGAGAGCCAAAACACTTGATGCGGAAGAAGTCGGTGCGCTCCTGCTTGTC
GCCGGCATCGTTGCGCCACTCTTCATTAACCGCTATATCGAAAATTGCTTGCGGCTTGTTAGAATTGCCATGAC
GTACCTCGGTGTCACGGGTAAGATTACCGATAAACTGGAACTGATTATGGCTCATATCGAAAGTCTCCTTGAG
AAAGGAGACTCTACTTTAGCTAAACATTGGTTCCGCTGTCAAGAACTTTAGCGGCTAAAATTTTGCGGGCCGCG
ACCAAAGGTGCGAGGGGCGGCTTCCGCTGTGTACAACCAGATATTTTTCACCAACATCCTTCGTCGCTCGATGA
GCGGGCATGACGAAACATGAGCTGTCGGAGAGGGCAGGGGTTTCAATTTCGTTTTTATCAGACTTAACCAACG
GTAAGGCCAACCCCTCGTTGAAGGTGATGGAGGCCATTGCCGACGCCCTGGAAACTCCCCTACCTCTTCTCCTG
GAGTCCACCGACCTTGACCGCGAGGCACTCGCGGAGATTGCGGGTCATCCTTTCAAGAGCAGCGTGCCGCCCG
GATACGAACGCATCAGTGTGGTTTTGCCGTCACATAAGGCGTTTATCGTAAAGAAATGGGGCGACGACACCCG
AAAAAAGCTGCGTGGAAGGCTCTGACGCCAAGGGTTAGGGCTTGCACTTCCTTCTTTAGCCGCTAAAACGGCC
CCTTCTCTGCGGGCCGTCGGCTCGCGCATCATATCGACATCCTCAACGGAAGCCGTGCCGCGAATGGCATCGG
GCGGGTGCGCTTTGACAGTTGTTTTCTATCAGAACCCCTACGTCGTGCGGTTCGATTAGCTGTTTGTCTTGCAG
GCTAAACACTTTCGGTATATCGTTTGCCTGTGCGATAATGTTGCTAATGATTTGTTGCGTAGGGGTTACTGAAA
AGTGAGCGGGAAAGAAGAGTTTCAGACCATCAAGGAGCGGGCCAAGCGCAAGCTGGAACGCGACATGGGTG
CGGACCTGTTGGCCGCGCTCAACGACCCGAAAACCGTTGAAGTCATGCTCAACGCGGACGGCAAGGTGTGGC
ACGAACGCCTTGGCGAGCCGATGCGGTACATCTGCGACATGCGGCCCAGCCAGTCGCAGGCGATTATAGAAA
CGGTGGCCGGATTCCACGGCAAAGAGGTCACGCGGCATTCGCCCATCCTGGAAGGCGAGTTCCCCTTGGATG
GCAGCCGCTTTGCCGGCCAATTGCCGCCGGTCGTGGCCGCGCCAACCTTTGCGATCCGCAAGCGCGCGGTCGC

CATCTTCACGCTGGAACAGTACGTCGAGGCGGGCATCATGACCCGCGAGCAATACGAGGTCATTAAAAGCGCC
GTGATTGATGATATAGCGGCCCGGCTGCTCCTGGTTCTCGCGCACCGAAATGGGTGACTTCACCCCGCGCTCTT
TGATCGTGGCACCGATTTCCGCGATGCTCTCCGGGGAAAAGCCGGGGTTGTCGGCCGTCCGCGGCTGATGCG
GATCTTCGTCGATCAGGTCCAGGTCCAGCTCGATAGGGCCGGAACCGCCCTGAGACGCCGCAGGAGCGTCCA
GGAGGCTCGACAGGTCGCCGATGCTATCCAACCCCAGGCCGGACGGCTGCGCCGCGCCTGCGGCTTCCTGAG
CGGCCGCAGCGGTGTTTTTCTTGGTGGTCTTGGCTTGAGCCGCAGTCATTGGGAAATCTCCATCTTCGTGAACA
CGTAATCAGCCAGGGCGCGAACCTCTTTCGATGCCTTGCGCGCGGCCGTTTTCTTGATCTTCCAGACCGGCACA
CCGGATGCGAGGGCATCGGCGATGCTGCTGCGCAGGCCAACGGTGGCCGGAATCATCATCTTGGGGTACGCG
GCCAGCAGCTCGGCTTGGTGGCGCGCGTGGCGCGGATTCCGCGCATCGACCTTGCTGGGCACCATGCCAAGG
AATTGCAGCTTGGCGTTCTTCTGGCGCACGTTCGCAATGGTCGTGACCATCTTCTTGATGCCCTGGATGCTGTA
CGCCTCAAGCTCGATGGGGGACAGCACATAGTCGGCCGCGAAGAGGGCGGCCGCCAGGCCGACGCCAAGGG
TCGGGGCCGTGTCGATCAGGCACACGTCGAAGCCTTGGTTCGCCAGGGCCTTGATGTTCGCCCCGAACAGCTC
GCGGGCGTCGTCCAGCGACAGCCGTTCGGCGTTCGCCAGTACCGGGTTGGACTCGATGAGGGCGAGGCGCGC
GGCCTGGCCGTCGCCGGCTGCGGGTGCGGTTTCGGTCCAGCCGCCGGCAGGGACAGCGCCGAACAGCTTGCT
TGCATGCAGGCCGGTAGCAAAGTCCTTGAGCGTGTAGGACGCATTGCCCTGGGGGTCCAGGTCGATCACGGC
AACCCGCAAGCCGCGCTCGAAAAAGTCGAAGGCAAGATGCACAAGGGTCGAAGTCTTGCCGACGCCGCCTTT
CTGGTTGGCCGTGACCAAAGTTTTCATCGTTTGGTTTCCTGTTTTTTCTTGGCGTCCGCTTCCCACTTCCGGACG
ATGTACGCCTGATGTTCCGGCAGAACCGCCGTTACCCGCGCGTACCCCTCGGGCAAGTTCTTGTCCTCGAACGC
GGCCCACACGCGATGCACCGCTTGCGACACTGCGCCCCTGGTCAGTCCCAGCGACGTTGCGAACGTCGCCTGT
GGCTTCCCATCGACTAAGACGCCCCGCGCTATCTCGATGGTCTGCTGCCCCACTTCCAGCCCCTGGATCGCCTC
CTGGAACTGGCTTTCGGTAAGCCGTTTCTTCATGGATAACACCCATAATTTGCTCCGCGCCTTGGTTGAACATA
GCGGTGACAGCCGCCAGCACATGAGAGAAGTTTAGCTAAACATTTCTCGCACGTCAACACCTTTAGCCGCTAA
AACTCGTCCTTGGCGTAACAAAACAAAAGCCCGGAAACCGGGCTTCGTCTCTTGCCGCTTATGGCTCTGCACCC
GGCTCCATCACCAACAGGTCGCGCACGCGCTTCACTCGGTTGCGGATCGACACTGCCAGCCCAACAAAGCCGG
TTGCCGCCGCCGCCAGGATCGCGCCGATGATGCCGGCCACACCGGCCATCGCCCACCAGGTCGCCGCCTTCCG
GTTCCATTCCTGCTGGTACTGCTTCGCAATGCTGGACCTCGGCTCACCATAGGCTGACCGCTCGATGGCGTATG
CCGCTTCTCCCCTTGGCGTAAAACCCAGCGCCGCAGGCGGCATTGCCATGCTGCCCGCCGCTTTCCCGACCACG
ACGCGCGCACCAGGCTTGCGGTCCAGACCTTCGGCCACGGCGAGCTGCGCAAGGACATAATCAGCCGCCGAC
TTGGCTCCACGCGCCTCGATCAGCTCTTGCACTCCGCGAAATCCTTGCCTCCACGGCCGCCATGAATCGCGCAC
GCGGCGAAGGCTCCGCAGGGCCGGCGTCGTGATCGCCGCCGAGAATGCCCTTCACCAAGTTCGACGACACGA
AAATCATGCTGACGGCTATCACCATCATGCAGACGATCGCACGAACCCGCTGAATTAATTCACCCCCGAACACG
AGCACGGCACCCGCGACCACTATGCCAAGAATGCCCAAGGTAAAAATTGCCGGCCCCGCCATGAAGTCCGTGA
ATGCCCCGACGGCCGAAGTGAAGGGCAGGCCGCCACCCAGGCCGCCGCCCTCACTGCCCGGCACCTGGTCGC
TGAATGTCGATGCCAGCACCTGCGGCACGTCAATGCTTCCGGGCGTCGCGCTCGGGCTGATCGCCCATCCCGT
TACTGCCCCGATCCCGGCAATGGCAAGGACTGCCAGCGCTGCCATTTTTGGGGTGAGGTCGTTCGCGGCCGAG
GGGCGCAGCCCCTGGGGGGATGGGGTGCCGCGTTAGCGGGCCGGGAGGGTTCGAGAAGGGGGGGCACCCC
CCTTCGGCGTGCGCGGTCACGCGCCAGGGCGCAGCCCTGGTTAAAAACAAGGTTTATAAATATTGGTTTAAAA
GCAGGTTAAAAGACAGGTTAGCGGTGGCCGAAAAACGGGCGGAAACCCTTGCAAATGCTGGATTTTCTGCCT
GTGGACAGCCCCTCAAATGTCAATAGGTGCGCCCCTCATCTGTCAGCACTCTGCCCCTCAAGTGTCAAGGATCG
CGCCCCTCATCTGTCAGTAGTCGCGCCCCTCAAGTGTCAATACCGCAGGGCACTTATCCCCAGGCTTGTCCACA
TCATCTGTGGGAAACTCGCGTAAAATCAGGCGTTTTCGCCGATTTGCGAGGCTGGCCAGCTCCACGTCGCCGG
CCGAAATCGAGCCTGCCCCTCATCTGTCAACGCCGCGCCGGGTGAGTCGGCCCCTCAAGTGTCAACGTCCGCC
CCTCATCTGTCAGTGAGGGCCAAGTTTTCCGCGAGGTATCCACAACGCCGGCGGCCGCGGTGTCTCGCACACG
GCTTCGACGGCGTTTCTGGCGCGTTTGCAGGGCCATAGACGGCCGCCAGCCCAGCGGCGAGGGCAACCAGCC
CGGTGAGCGTCGGAAAGGCGCTGGAAGCCCCGTAGCGACGCGGAGAGGGGCGAGACAAGCCAAGGGCGCA
GGCTCGAGTCGCAGCACGACATAGCCGGTTCTCGCAAGGACGAGAATTTCCCTGCGGTGCCCCTCAAGTGTCA
ATGAAAGTTTCCAACGCGAGCCATTCGCGAGAGCCTTGAGTCCACGCTAGATCT

>Complete sequence of the pRW-(*lacO*)$_8$ plasmid. Features of the plasmid are shown in different colours: recognition site for the yeast meganuclease I-SceI in orange, binding sites for the LacI repressor in blue and the target sites for the EcoRI and HindIII restriction enzymes in red.

I-SceI LacI

GAATTGTAGGGATAACAGGGTAATGAATTGAGCCAGCACGTAGCTAGCAGAGAGTAAGGAATTGTGAGCGG

LacI LacI

ATAACAATTAAAAGTCAGAAATTGTGAGCGGATAACAATTAAAGACGGAGAATTGTGAGCGGATAACAATTA

LacI LacI

ATAAAGCAGCTCTAGCATCAACGCGCAAATTGTGAGCGGATAACAATTAGCAAGACAGAATTGTGAGCGGAT

EcoRI

AACAATTCTGGGAATTCCGTAGCTAGCAGAGTAGTAAGGAATTGTGAGCGGATAACTTAAAAGTCAGAAATTG

LacI LacI

TGAGCGGATAACAATTAAAGACGGAGAATTGTGAGCGGATAACAATTAATAAAGCAGCTCTAGCATCAACGC

HindIII

GCAAGCTTTTGTCAGTGCGCAAAAAGATCCTGAATTTCAGGCTCAGTTCAACGACCTGCTGAAAAACTATGCCG
GGCGTCCAACCGCGCTGACCAAATGCCAGAACATTACAGCCGGGACGAACACCACGCTGTATCTCAAGCGTGA
AGATTTGCTGCACGGCGGCGCGCATAAAACTAACCAGGTGCTGGGGCAGGCGTTGCTGGCGAAGCGGATGG
GTAAAACCGAAATCATCGCCGAAACCGGTGCCGGTCAGCATGGCGTGGCGTCGGCCCTTGCCAGCGCCCTGCT
CGGCCTGAAATGCCATATTTATATGGGTGCCAAAGACGTTGAACGCCAGTCGCCTAACGTTTTTCGTATGCGCT
TAATGGGTGCGGAAGTGATCCCGGTGCATAGCGGTTCCGCGACGCTGAAAGATGCCTGTAACGAGGCGCTGC
GCGACTGGTCCGGTAGTTACGAAACCGCGCACTATATGCTGGGCACCGCAGCTGGCCCGCATCCTTATCCGAC
CATTGTGCGTGAGTTTCAGCGGATGATTGGCGAAGAAACCAAAGCGCAGATTCTGGAAAGAGAAGGTCGCCT
GCCGGATGCCGTTATCGCCTGTGTTGGCGGCGGTTCGAATGCCATCGGCATGTTTGCTGATTTCATCAATGAAA
CCAACGTCGGCCTGATTGGTGTGGAGCCAGGTGGTCACGGTATCGAAACTGGCGAGCACGGCGCACCGCTAA
AACATGGTCGCGTGGGTATCTATTTCGGTATGAAAGCGCCGATGATGCAAACCGAAGACGGGCAGATTGAAG
AATCTTACTCCATCTCCGCCGGACTGGATTTCCCGTCTGTCGGCCCACAACACGCGCAGAGGAAGCGAAGCTG
CGCGTCGGCCGTTTCCATCTGCGGTGCGCCCGGTCGCGTGCCGGCATGGATGCGCGCGCCATCGCGGTAGGC
GAGCAGCGCCTGCCTGAAGCTGCGGGCATTCCCGATCAGAAATGAGCGCCAGTCGTCGTCGGCTCTCGGCACC
GAATGCGTATGATTCTCCGCCAGCATGGCTTCGGCCAGTGCGTCGAGCAGCGCCCGCTTGTTCCTGAAGTGCC
AGTAAAGCGCCGGCTGCTGAACCCCCAACCGTTCCGCCAGTTTGCGTGTCGTCAGACCGTCTACGCCGACCTC
GTTCAACAGGTCCAGGGCGGCACGGATCACTGTATTCGGCTGCAACTTTGTCATGCTTGACACTTTATCACTGA
TAAACATAATATGTCCACCAACTTATCAGTGATAAAGAATCCGCGCGTTCAATCGGACCAGCGGAGGCTGGTC
CGGAGGCCAGACGTGAAACCCAACATACCCCTGATCGTAATTCTGAGCACTGTCGCGCTCGACGCTGTCGGCA
TCGGCCTGATTATGCCGGTGCTGCCGGGCCTCCTGCGCGATCTGGTTCACTCGAACGACGTCACCGCCCACTAT
GGCATTCTGCTGGCGCTGTATGCGTTGGTGCAATTTGCCTGCGCACCTGTGCTGGGCGCGCTGTCGGATCGTTT
CGGGCGGCGGCCAATCTTGCTCGTCTCGCTGGCCGGCGCCACTGTCGACTACGCCATCATGGCGACAGCGCCT
TTCCTTTGGGTTCTCTATATCGGGCGGATCGTGGCCGGCATCACCGGGGCGACTGGGGCGGTAGCCGGCGCTT
ATATTGCCGATATCACTGATGGCGATGAGCGCGCGCGGCACTTCGGCTTCATGAGCGCCTGTTTCGGGTTCGG
GATGGTCGCGGGACCTGTGCTCGGTGGGCTGATGGGCGGTTTCTCCCCCCACGCTCCGTTCTTCGCCGCGGCA
GCCTTGAACGGCCTCAATTTCCTGACGGGCTGTTTCCTTTTGCCGGAGTCGCACAAAGGCGAACGCCGGCCGTT

ACGCCGGGAGGCTCTCAACCCGCTCGCTTCGTTCCGGTGGGCCCGGGGCATGACCGTCGTCGCCGCCCTGATG
GCGGTCTTCTTCATCATGCAACTTGTCGGACAGGTGCCGGCCGCGCTTTGGGTCATTTTCGGCGAGGATCGCTT
TCACTGGGACGCGACCACGATCGGCATTTCGCTTGCCGCATTTGGCATTCTGCATTCACTCGCCCAGGCAATGA
TCACCGGCCCTGTAGCCGCCCGGCTCGGCGAAAGGCGGGCACTCATGCTCGGAATGATTGCCGACGGCACAG
GCTACATCCTGCTTGCCTTCGCGACACGGGGATGGATGGCGTTCCCGATCATGGTCCTGCTTGCTTCGGGTGG
CATCGGAATGCCGGCGCTGCAAGCAATGTTGTCCAGGCAGGTGGATGAGGAACGTCAGGGGCAGCTGCAAG
GCTCACTGGCGGCGCTCACCAGCCTGACCTCGATCGTCGGACCCCTCCTCTTCACGGCGATCTATGCGGCTTCT
ATAACAACGTGGAACGGGTGGGCATGGATTGCAGGCGCTGCCCTCTACTTGCTCTGCCTGCCGGCGCTGCGTC
GCGGGCTTTGGAGCGGCGCAGGGCAACGAGCCGATCGCTGATCGTGGAAACGATAGGCCTATGCCATGCGG
GTCAAGGCGACTTCCGGCAAGCTATACGCGCCCTAGGAGTGCGGTTGGAACGTTGGCCCAGCCAGATACTCCC
GATCACGAGCAGGACGCCGATGATTTGAAGCGCACTCAGCGTCTGATCCAAGAACAACCATCCTAGCAACACG
GCGGTCCCCGGGCTGAGAAAGCCCAGTAAGGAAACAACTGTAGGTTCGAGTCGCGAGATCCCCCGGAACCAA
AGGAAGTAGGTTAAACCCGCTCCGATCAGGCCGAGCCACGCCAGGCCGAGAACATTGGTTCCTGTAGGCATC
GGGATTGGCGGATCAAACACTAAAGCTACTGGAACGAGCAGAAGTCCTCCGGCCGCCAGTTGCCAGGCGGTA
AAGGTGAGCAGAGGCACGGGAGGTTGCCACTTGCGGGTCAGCACGGTTCCGAACGCCATGGAAACCGCCCCC
GCCAGGCCCGCTGCGACGCCGACAGGATCTAGCGCTGCGTTTGGTGTCAACACCAACAGCGCCACGCCCGCA
GTTCCGCAAATAGCCCCCAGGACCGCCATCAATCGTATCGGGCTACCTAGCAGAGCGGCAGAGATGAACACG
ACCATCAGCGGCTGCACAGCGCCTACCGTCGCCGCGACCCCGCCCGGCAGGCGGTAGACCGAAATAAACAAC
AAGCTCCAGAATAGCGAAATATTAAGTGCGCCGAGGATGAAGATGCGCATCCACCAGATTCCCGTTGGAATCT
GTCGGACGATCATCACGAGCAATAAACCCGCCGGCAACGCCCGCAGCAGCATACCGGCGACCCCTCGGCCTCG
CTGTTCGGGCTCCACGAAAACGCCGGACAGATGCGCCTTGTGAGCGTCCTTGGGGCCGTCCTCCTGTTTGAAG
ACCGACAGCCCAATGATCTCGCCGTCGATGTAGGCGCCGAATGCCACGGCATCTCGCAACCGTTCAGCGAACG
CCTCCATGGGCTTTTTCTCCTCGTGCTCGTAAACGGACCCGAACATCTCTGGAGCTTTCTTCAGGGCCGACAATC
GGATCTCGCGGAAATCCTGCACGTCGGCCGCTCCAAGCCGTCGAATCTGAGCCTTAATCACAATTGTCAATTTT
AATCCTCTGTTTATCGGCAGTTCGTAGAGCGCGCCGTGCGTCCCGAGCGATACTGAGCGAAGCAAGTGCGTCG
AGCAGTGCCCGCTTGTTCCTGAAATGCCAGTAAAGCGCTGGCTGCTGAACCCCCAGCCGGAACTGACCCCACA
AGGCCCTAGCGTTTGCAATGCACCAGGTCATCATTGACCCAGGCGTGTTCCACCAGGCCGCTGCCTCGCAACTC
TTCGCAGGCTTCGCCGACCTGCTCGCGCCACTTCTTCACGCGGGTGGAATCCGATCCGCACATGAGGCGGAAG
GTTTCCAGCTTGAGCGGGTACGGCTCCCGGTGCGAGCTGAAATAGTCGAACATCCGTCGGGCCGTCGGCGAC
AGCTTGCGGTACTTCTCCCATATGAATTTCGTGTAGTGGTCGCCAGCAAACAGCACGACGATTTCCTCGTCGAT
CAGGACCTGGCAACGGGACGTTTTCTTGCCACGGTCCAGGACGCGGAAGCGGTGCAGCAGCGACACCGATTC
CAGGTGCCCAACGCGGTCGGACGTGAAGCCCATCGCCGTCGCCTGTAGGCGCGACAGGCATTCCTCGGCCTTC
GTGTAATACCGGCCATTGATCGACCAGCCCAGGTCCTGGCAAAGCTCGTAGAACGTGAAGGTGATCGGCTCGC
CGATAGGGGTGCGCTTCGCGTACTCCAACACCTGCTGCCACACCAGTTCGTCATCGTCGGCCCGCAGCTCGAC
GCCGGTGTAGGTGATCTTCACGTCCTTGTTGACGTGGAAAATGACCTTGTTTTGCAGCGCCTCGCGCGGGATTT
TCTTGTTGCGCGTGGTGAACAGGGCAGAGCGGGCCGTGTCGTTTGGCATCGCTCGCATCGTGTCCGGCCACG
GCGCAATATCGAACAAGGAAAGCTGCATTTCCTTGATCTGCTGCTTCGTGTGTTTCAGCAACGCGGCCTGCTTG
GCCTCGCTGACCTGTTTTGCCAGGTCCTCGCCGGCGGTTTTTCGCTTCTTGGTCGTCATAGTTCCTCGCGTGTCG
ATGGTCATCGACTTCGCCAAACCTGCCGCCTCCTGTTCGAGACGACGCGAACGCTCCACGGCGGCCGATGGCG
CGGGCAGGGCAGGGGGAGCCAGTTGCACGCTGTCGCGCTCGATCTTGGCCGTAGCTTGCTGGACCATCGAGC
CGACGGACTGGAAGGTTTCGCGGGGCGCACGCATGACGGTGCGGCTTGCGATGGTTTCGGCATCCTCGGCGG
AAAACCCCGCGTCGATCAGTTCTTGCCTGTATGCCTTCCGGTCAAACGTCCGATTCATTCACCCTCCTTGCGGGA
TTGCCCCGACTCACGCCGGGGCAATGTGCCCTTATTCCTGATTTGACCCGCCTGGTGCCTTGGTGTCCAGATAA
TCCACCTTATCGGCAATGAAGTCGGTCCCGTAGACCGTCTGGCCGTCCTTCTCGTACTTGGTATTCCGAATCTTG
CCCTGCACGAATACCAGCGACCCCTTGCCCAAATACTTGCCGTGGGCCTCGGCCTGAGAGCCAAAACACTTGA
TGCGGAAGAAGTCGGTGCGCTCCTGCTTGTCGCCGGCATCGTTGCGCCACTCTTCATTAACCGCTATATCGAAA
ATTGCTTGCGGCTTGTTAGAATTGCCATGACGTACCTCGGTGTCACGGGTAAGATTACCGATAAACTGGAACT
GATTATGGCTCATATCGAAAGTCTCCTTGAGAAAGGAGACTCTAGTTTAGCTAAACATTGGTTCCGCTGTCAAG

AACTTTAGCGGCTAAAATTTTGCGGGCCGCGACCAAAGGTGCGAGGGGCGGCTTCCGCTGTGTACAACCAGA
TATTTTTCACCAACATCCTTCGTCTGCTCGATGAGCGGGGCATGACGAACATGAGCTGTCGGAGAGGGCAGGG
GTTTCAATTTCGTTTTTATCAGACTTAACCAACGGTAAGGCCAACCCCTCGTTGAAGGTGATGGAGGCCATTGC
CGACGCCCTGGAAACTCCCCTACCTCTTCTCCTGGAGTCCACCGACCTTGACCGCGAGGCACTCGCGGAGATTG
CGGGTCATCCTTTCAAGAGCAGCGTGCCGCCCGGATACGAACGCATCAGTGTGGTTTTGCCGTCACATAAGGC
GTTTATCGTAAAGAAATGGGGCGACGACACCCGAAAAAAGCTGCGTGGAAGGCTCTGACGCCAAGGGTTAGG
GCTTGCACTTCCTTCTTTAGCCGCTAAAACGGCCCCTTCTCTGCGGGCCGTCGGCTCGCGCATCATATCGACATC
CTCAACGGAAGCCGTGCCGCGAATGGCATCGGGCGGGTGCGCTTTGACAGTTGTTTTCTATCAGAACCCCTAC
GTCGTGCGGTTCGATTAGCTGTTTGTCTTGCAGGCTAAACACTTTCGGTATATCGTTTGCCTGTGCGATAATGTT
GCTAATGATTTGTTGCGTAGGGGTTACTGAAAAGTGAGCGGGAAAGAAGAGTTTCAGACCATCAAGGAGCGG
GCCAAGCGCAAGCTGGAACGCGACATGGGTGCGGACCTGTTGGCCGCGCTCAACGACCCGAAAACCGTTGAA
GTCATGCTCAACGCGGACGGCAAGGTGTGGCACGAACGCCTTGGCGAGCCGATGCGGTACATCTGCGACATG
CGGCCCAGCCAGTCGCAGGCGATTATAGAAACGGTGGCCGGATTCCACGGCAAAGAGGTCACGCGGCATTCG
CCCATCCTGGAAGGCGAGTTCCCCTTGGATGGCAGCCGCTTTGCCGGCCAATTGCCGCCGGTCGTGGCCGCGC
CAACCTTTGCGATCCGCAAGCGCGCGGTCGCCATCTTCACGCTGGAACAGTACGTCGAGGCGGGCATCATGAC
CCGCGAGCAATACGAGGTCATTAAAAAGCGCCGTGATTGATGATATAGCGGCCCGGCTGCTCCTGGTTCTCGC
GCACCGAAATGGGTGACTTCACCCCGCGCTCTTTGATCGTGGCACCGATTTCCGCGATGCTCTCCGGGGAAAA
GCCGGGGTTGTCGGCCGTCCGCGGCTGATGCGGATCTTCGTCGATCAGGTCCAGGTCCAGCTCGATAGGGCC
GGAACCGCCCTGAGACGCCGCAGGAGCGTCCAGGAGGCTCGACAGGTCGCCGATGCTATCCAACCCCAGGCC
GGACGGCTGCGCCGCGCCTGCGGCTTCCTGAGCGGCCGCAGCGGTGTTTTTCTTGGTGGTCTTGGCTTGAGCC
GCAGTCATTGGGAAATCTCCATCTTCGTGAACACGTAATCAGCCAGGGCGCGAACCTCTTTCGATGCCTTGCGC
GCGGCCGTTTTCTTGATCTTCCAGACCGGCACACCGGATGCGAGGGCATCGGCGATGCTGCTGCGCAGGCCAA
CGGTGGCCGGAATCATCATCTTGGGGTACGCGGCCAGCAGCTCGGCTTGGTGGCGCGCGTGGCGCGGATTCC
GCGCATCGACCTTGCTGGGCACCATGCCAAGGAATTGCAGCTTGGCGTTCTTCTGGCGCACGTTCGCAATGGT
CGTGACCATCTTCTTGATGCCCTGGATGCTGTACGCCTCAAGCTCGATGGGGGACAGCACATAGTCGGCCGCG
AAGAGGGCGGCCGCCAGGCCGACGCCAAGGGTCGGGGCCGTGTCGATCAGGCACACGTCGAAGCCTTGGTT
CGCCAGGGCCTTGATGTTCGCCCCGAACAGCTCGCGGGCGTCGTCCAGCGACAGCCGTTCGGCGTTCGCCAGT
ACCGGGTTGGACTCGATGAGGGCGAGGCGCGCGGCCTGGCCGTCGCCGGCTGCGGGTGCGGTTTCGGTCCA
GCCGCCGGCAGGGACAGCGCCGAACAGCTTGCTTGCATGCAGGCCGGTAGCAAAGTCCTTGAGCGTGTAGGA
CGCATTGCCCTGGGGGTCCAGGTCGATCACGGCAACCCGCAAGCCGCGCTCGAAAAAGTCGAAGGCAAGATG
CACAAGGGTCGAAGTCTTGCCGACGCCGCCTTTCTGGTTGGCCGTGACCAAAGTTTTCATCGTTTGGTTTCCTG
TTTTTTCTTGGCGTCCGCTTCCCACTTCCGGACGATGTACGCCTGATGTTCCGGCAGAACCGCCGTTACCCGCGC
GTACCCCTCGGGCAAGTTCTTGTCCTCGAACGCGGCCCACACGCGATGCACCGCTTGCGACACTGCGCCCCTG
GTCAGTCCCAGCGACGTTGCGAACGTCGCCTGTGGCTTCCCATCGACTAAGACGCCCCGCGCTATCTCGATGGT
CTGCTGCCCCACTTCCAGCCCCTGGATCGCCTCCTGGAACTGGCTTTCGGTAAGCCGTTTCTTCATGGATAACAC
CCATAATTTGCTCCGCGCCTTGGTTGAACATAGCGGTGACAGCCGCCAGCACATGAGAGAAGTTTAGCTAAAC
ATTTCTCGCACGTCAACACCTTTAGCCGCTAAAACTCGTCCTTGGCGTAACAAAACAAAAGCCCGGAAACCGGG
CTTTCGTCTCTTGCCGCTTATGGCTCTGCACCCGGCTCCATCACCAACAGGTCGCGCACGCGCTTCACTCGGTTG
CGGATCGACACTGCCAGCCCAACAAAGCCGGTTGCCGCCGCCGCCAGGATCGCGCCGATGATGCCGGCCACA
CCGGCCATCGCCCACCAGGTCGCCGCCTTCCGGTTCCATTCCTGCTGGTACTGCTTCGCAATGCTGGACCTCGG
CTCACCATAGGCTGACCGCTCGATGGCGTATGCCGCTTCTCCCCTTGGCGTAAAACCCAGCGCCGCAGGCGGC
ATTGCCATGCTGCCCGCCGCTTTCCCGACCACGACGCGCGCACCAGGCTTGCGGTCCAGACCTTCGGCCACGG
CGAGCTGCGCAAGGACATAATCAGCCGCCGACTTGGCTCCACGCGCCTCGATCAGCTCTTGCACTCGCGCGAA
ATCCTTGGCCTCCACGGCCGCCATGAATCGCGCACGCGGCGAAGGCTCCGCAGGGCCGGCGTCGTGATCGCC
GCCGAGAATGCCCTTCACCAAGTTCGACGACACGAAAATCATGCTGACGGCTATCACCATCATGCAGACGGAT
CGCACGAACCCGCTGAATTAATTCACCCCCGAACACGAGCACGGCACCCGCGACCACTATGCCAAGAATGCCC
AAGGTAAAAATTGCCGGCCCCGCCATGAAGTCCGTGAATGCCCCGACGGCCGAAGTGAAGGGCAGGCCGCCA
CCCAGGCCGCCGCCCTCACTGCCCGGCACCTGGTCGCTGAATGTCGATGCCAGCACCTGCGGCACGTCAATGC

TTCCGGGCGTCGCGCTCGGGCTGATCGCCCATCCCGTTACTGCCCCGATCCCGGCAATGGCAAGGACTGCCAG
CGCTGCCATTTTTGGGGTGAGGCCGTTCGCGGCCGAGGGGCGCAGCCCCTGGGGGGATGGGAGGCCCGCGT
TAGCGGGCCGGGAGGGTTCGAGAAGGGGGGGCACCCCCCTTCGGCGTGCGCGGTCACGCGCACAGGGCGCA
GCCCTGGGTTAAAACAAGGTTTATAAATATTGGTTTAAAAGCAGGTTAAAAGACAGGTTAGCGGTGGCCGAAA
AACGGGCGGAAACCCTTGCAAATGCTGGATTTTCTGCCTGTGGACAGCCCCTCAAATGTCAATAGGTGCGCCC
CTCATCTGTCAGCACTCTGCCCCTCAAGTGTCAAGGATCGCGCCCCTCATCTGTCAGTAGTCGCGCCCCTCAAGT
GTCAATACCGCAGGGCACTTATCCCCAGGCTTGTCCACATCATCTGTGGGAAACTCGCGTAAAATCAGGCGTTT
TCGCCGATTTGCGAGGCTGGCCAGCTCCACGTCGCCGGCCGAAATCGAGCCTGCCCCTCATCTGTCAGTGAGG
GCCAAGTTTTCCGCGAGGTATCCACAACGCCGGCGGCCGCGGTGTCTCGCACACGGCTTCGACGGCGTTTCTG
GCGCGTTTGCAGGGCCATAGACGGCCGCCAGCCCAGCGGCGAGGGCAACCAGCCCGGTGAGCGTCGGAAAG
GCGCTGGAAGCCCCGTAGCGACGCGGAGAGGGGCGAGACAAGCCAAGGGCGCAGGCTCGATGCGCAGCAC
GACATAGCCGGTTCTCGCAAGGACGAGAATTTCCCTGCGGTGCCCCTCAAGTGTCAATGAAAGTTTCCAACGC
GAGCCATTCGCGAGAGCCTTGAGTCCACGCTAGATCT

>Complete sequence of the pRW-LexA plasmid. Features of the plasmid are shown in different colours: LexA (*E. coli*) promoter and terminator in purple, *lexA$_{BT}$* in green, recognition sites for the yeast meganuclease I-SceI in orange, binding sites for the LacI repressor in blue and binding sites for the LexA$_{BT}$ in pale orange.

LexA (*E. coli*) promoter

GATCCGTCGACCTGCAGCCAAGCTTACCCTTCCAGAATTCGATAAATCTCTGGTTTATTGTGCAGTTTATGGTTC
CAAAATCGCCTTTTGCTGTATATACTCACAGCATAACTGTATATACACCCAGGGGGCGGAATGTTAGAAAACAT

lexA$_{BT}$

GGAAAAGTTAACGAAACGCCAGCAAGACATTCTCGACTTTATTAAGCTAAAAGTACAAGAAAAAGGATATCCA
CCTTCCGTACGTGAAATTGGTCAAGCAGTCGGCCTCGCTTCTAGTTCTACAGTGCACGGACATTTATCACGATT
AGAAGAAAAAGGATACATTCGACGCGATCCAACAAAACCACGTGCCATTGAAATTTTAGGCGAAGACCGAAT
GGATACGGAAACACAATCTGTTATTCAAGTTCCAATTGTCGGAAAAGTTACTGATGGTTTACCAATTACAGCGG
TCGAAAGCGTAGAGGAACATTTCCCACTTCCAGCTAGCATTGTTTCCGGAGCAGACCAAGTGTTCATGTTACGT
ATTTCTGGAGATAGTATGATTGAAGCTGGTATTTTCGATGGAGATTTAGTTGTTGTTCGCCAACAACAGTCTGC
ATATAATGGTGAAATTGTAGTCGCTTTAACAGAAGATAACGAAGCAACTGTTAAACGTTTCTATAAAGAAAAA
GACCATTTCCGTCTACAACCAGAAAACTCTTCATTAGAACCTATCATTTTAAAACAAGTGTCAGTTATCGGGAAA

terminator

GTAATTGGCGTATATCGTGATTTACATTAACCGATTACGTGCCTGGCGGCAGTAGCGCGGTGGTCCCACCTGA
CCCCATGCCGAACTCAGAAGTGAAACGCCGTAGCGCCGATGGTAGTGTGGGGTCTCCCCATGCGAGAGTAGG
GAACTGCCAGGCATCAAATAAAACGAAAGGCTCAGTCGAAAGACTGGGCCTTTTCGAATTTGGTCGCGCGCG
GATGTTTGGCTGTGTTGGCTGGGCGCTGTGTGCCTCGATTGTCGGCATCATGTTCACCATCAATAATCAGTTTG
TTTTCTGGCTGGGCTCTGGCTGTGCACTCATCCTCGCCGTTTTACTCTTTTTCGCCAAAACGGATGCGCCCTCTTC
TGCCACGGTTGCCAATGCGGTAGGTGCCAACCATTCGGCATTTAGCCTTAAGCTGGCACTGGAACTGTTCAGA
CAGCCAAAACTGTGGTTTTTGTCACTGTATGTTATTGGCGTTTCCTGCACCTACGATGTTTTTGACCAACAGTTT
GCTAATTTCTTTACTTCGTTCTTTGCTACCGGTGAACAGGGTACGCGGGTATTTGGCTACGTAACGACAATGGG
CGAATTACTTAACGCCTCGATTATGTTCTTTGCGCCACTGATCATTAATCGCATCGGTGGGAAAAACGCCCTGC
TGCTGGCTGGCACTATTATGTCTGTACGTATTATTGGCTCATCGTTCGCCACCTCAGCGCTGGAAGTGGTTATTC
TGAAAACGCTGCATATGTTTGAAGTACCGTTCCTGCTGGTGGGCTGCTTTAAATATATTACCAGCCAGTTTGAA
GTGCGTTTTTCAGCGACGATTTATCTGGTCTGTTTCTGCTTCTTTAAGCAACTGGCGATGATTTTTATGTCTGTAC
TGGCGGGCAATATGTATGAAAGCATCGGTTTCCAGGGCGCTTATCTGGTGCTGGGTCTGGTGGCGCTGGGCTT
CACCTTAATTTCCGTGTTCACGCTTAGCGGCCCCGGCCCGCTTTCCCTGCTGCGTCGTCAGGTGAATGAAGTCG
CTTAAGCAATCAATGTCGGATGCGGCGCGACGCTTATCCGACCAACATATCATAACGGAGTGATCGCATTGAA
CATGCCAATGACCGAAAGAATAAGAGCAGGCAAGCTATTTACCGATATGTGCGAAGGCTTACCGGAAAAAAG
ACTTCGTGGGAAAACGTTAATGTATGAGTTTAATCACTCGCATCCATCAGAAGTTGAAAAAAGAGAAAGCCTG
ATTAAAGAAATGTTTGCCACGGTAGGGGAAAACGCCTGGGTAGAACCGCCTGTCTATTTCTCTTACGGTTCCAA
CATCCATATAGGCCGCAATTTTTATGCAAATTTCAATTTAACCATTGTCGATGACTACACGGTAACAATCGGTGA
TAACGTACTGATTGCACCCAACGTTACTCTTTCCGTTACGGGACACCCTGTACACCATGAATTGAGAAAAAACG
GCGAGATGTACTCTTTTCCGATAACGATTGGCAATAACGTCTGGATCGGAAGTCATGTGGTTATTAATCCAGGC
GTCACCATCGGGGATAATTCTGTTATTGGCGCGGGTAGTATCGTCACAAAAGACATTCCACCAAACGTCGTGG
CGGCTGGCGTTCCTTGTCGGGTTATTCGCGAAATAAACGACCGGGATAAGCACTATTATTTCAAAGATTATAAA
GTTGAATCGTCAGTTTAAATTATAAAAATTGCCTGATACGCTGCGCTTATCAGGCCTACAAGTTCAGCGATCTA
CATTAGCCGCATCCGGCATGAACAAAGCGCAGGAACAAGCGTCGCATCATGCCTCTTTGACCCACAGCTGCGG
AAAACGTACTGGTGCAAAACGCAGGGTTATGATCATCAGCCCAACGACGCACAGCGCATGAAATGCCCAGTCC
ATCAGGTAATTGCCGCTGATACTACGCAGCACGCCAGAAAACCACGGGGCAAGCCCGGCGATGATAAAACCG
ATTCCCTGCATAAACGCCACCAGCTTGCCAGCAATAGCCGGTTGCACAGAGTGATCGAGCGCCAGCAGCAAAC
AGAGCGGAAACGCGCCGCCCAGACCTAACCCACACACCATCGCCCACAATACCGGCAATTGCATCGGCAGCCA
GATAAAGCCGCAGAACCCCACCAGTTGTAACACCAGCGCCAGCATTAACAGTTTGCGCCGATCCTGATGGCGA
GCCATAGCAGGCATCAGCAAAGCTCCTGCGGCTTGCCCAAGCGTCATCAATGCCAGTAAGGAACCGCTGTACT
GCGCGCTGGCACCAATCTCAATATAGAAAGCGGGTTACCATGGGCGGGCTGGCGCTGGCCGGAAGCTGGCTT
CATCAGCATGTCAGCGAACGTCGCAGTGTCGCCATCAGTCTGTTGCTGATTGCCGTCGGTGCATTGATGCGTG
AGCTTTACCCGCAAAGTGCGCTGCTGCTTAGCAGCGCACTGCTTGGTGGGGTGGGGATCGGCATCATTCAGGC

GGTGATGCCTTCGGTGATTAAACGGCGGTTTCAGCAGCGCACGCCACTGGTGATGGGGCTGTGGTCCGCGGC
TCTGATGGGCGGCGGTGGGCTTGGTGCCGCCATAACGCCCTGGTTAGTTCAACATAGCGAAACCTGGTATCAA
ACACTCGCCTGGTGGGCGCTGCCTGCCGTTGTTGCGCTCTTTGCCTGGTGGTGGCAAAGCGCCCGCGAGGTCG
CCTCTTCCCACAAGACAACAACCACTCCGGTTCGCGTGGTATTCACTCCCCGCGCGTGGACGCTGGGTGTTTAC
TTCGGTCTGATTAACGGCGGTTACGCCAGCCTGATTGCCTGGTTACCGCCAAGCTGCAGCCAAGCTAGCTTTTG
CCATTCTCACCGGATTCAGTCGTCACTCATGGTGATTTCTCACTTGATAACCTTATTTTTGACGAGGGGAAATTA
ATAGGTTGTATTGATGTTGGACGAGTCGGAATCGCAGACCGATACCAGGATCTTGCCATCCTATGGAACTGCC
TCGGTGAGTTTTCTCCTTCATTACAGAAACGGCTTTTTCAAAAATATGGTATTGATAATCCTGATATGAATAAAT
TGCAGTTTCATTTGATGCTGGATGAGTTTTTCTAATCAGAATTGGTTAATTGGTTGTAACACTGGCAGAGCATT
ACGCTGACTTGACGGGACGGCGGCTTTGTTGAATAAATCGAACTTTTGCTGAGTTGAAGGATCAGATCACGCA
TCTTCCCGACAACGCAGACCGTTCCGTGGCAAAGCAAAAGTTCAAAATCACCAACTGGTCCACCTACAACAAA
GCTCTCATCAACCGTGGCTCCCTCACTTTCTGGCTGGATGATGGGGCGATTCAGGCCTGGTATGAGTCAGCAAC
ACCTTCTTCACGAGGCAGACCTCAGCGCCAGAAGGCCGCCAGAGAGGCCGAGCGCGGCGTGAGGCTTGGAC
GCTAGGGCAGGGCATGAAAAAGCCCGTAGCGGGCGCTACGGGGCTCTGACGCGGTGGAAAGGGGGAGGGG
ATGTGTTGTCTACATGGCTCTGCTGTAGTGAGTGGGTTGCGCTCCGGCAGCGGTCCTGATCAATCGTCACCCTT
TCTCGGTCCTTCAACGTTCCTGACAACGAGCCTCCTTTTCGCCAATCCATCGACAATCACCGCGAGTCCCTGCTC
GAACGCTGCGTCCGGACCGGCTTCGTCGAAGGCGTCTATCGCGGCCCGCAACAGCGGCGGAGAGCGGAGCCT
GTTCAACGGTGCCGCCGCGCTCGCCGGACTCGCTGTCGCCGGCCTGCTCCTCAAGCACGGCCCCAACAGTGAA
GTAGCTGATTGTCATCAGCGCATTGACGGCGTCCCCGGCCGAAAAACCCGCCTCGCAGAGGAAGCGAAGCTG
CGCGTCGGCCGTTTCCATCTGCGGTGCGCCCGGTCGCGTGCCGGCATGGATGCGCGCGCCATCGCGGTAGGC
GAGCAGCGCCTGCCTGAAGCTGCGGGCATTCCCAGTCAGAAATGAGCGCCAGTCGTCGTCGGCTCTCGGCACC
GAAGTGCTATGATTCTCCGCCAGCATGGCTTCGGCCAGTGCGTCGAGCAGCGCCCGCTTGTTCCTGAAGTGCC
AGTAAAGCGCCGGCTGCTGAACCCCCAACCGTTCCGCCAGTTTGCGTGTCGTCAGACCGTCTACGCCGACCTC
GTTCAACAGGTCTAGGGCGGCACGGATCACTGTATTCGGCTGCAACTTTGTCATGCTTGACACTTTATCACTGA
TAAACATAATATGTCCACCAACTTATCAGTGATAAAGAATCCGCGCGTTCAATCGGACCAGCGGAGGCTGGTC
CGGAGGCCAGACGTGAAACCCAACATACCCCTGATCGTAATTCTGAGCACTGTCGCGCTCGACGCTGTCGGCA
TCGGCCTGATTATGCCGGTGCTGCCGGGCCTCCTGCGCGATCTGGTTCACTCGAACGACGTCACCGCCCACTAT
GGCATTCTGCTGGCGCTGTATGCGTTGGTGCAATTTGCCTGCGCACCTGTGCTGGGCGCGCTGTCGGATCGTTT
CGGGCGGCGGCCAATCTTGCTCGTCTCGCTGGCCGGCGCCACTGTCGACTACGCCATCATGGCGACAGCGCCT
TTCCTTTGGGTTCTCTATATCGGGCGGATCGTGGCCGGCATCACCGGGGCGACTGGGGCGGTAGCCGGCGCTT
ATATTGCCGATATCACTGATGGCGATGAGCGCGCGCGGCACTTCGGCTTCATGAGCGCCTGTTTCGGGTTCGG
GATGGTCGCGGGACCTGTGCTCGGTGGGCTGATGGGCGGTTTCTCCCCCCACGCTCCGTTCTTCGCCGCGGCA
GCCTTGAACGGCCTCAATTTCCTGACGGGCTGTTTCCTTTTGCCGGAGTCGCACAAAGGCGAACGCCGGCCGTT
ACGCCGGGAGGCTCTCAACCCGCTCAGCTTCGTTCGGTGGGCCCGGGGCATGACCGTCGTCGCCGCCCTGATG
GCGGTCTTCTTCATCATGCAACTTGTCGGACAGGTGCCGGCCGCGCTTTGGGTCATTTTCGGCGAGGATCGCTT
TCACTGGGACGCGACCACGATCGGCATTTCGCTTGCCGCATTTGGCATTCTGCATTCACTCGCCCAGGCAATGA
TCACCGGCCCTGTAGCCGCCCGGCTCGGCGAAAGGCGGGCACTCATGCTCGGAATGATTGCCGACGGCACAG
GCTACATCCTGCTTGCCTTCGCGACACGGGGATGGATGGCGTTCCCGATCATGGTCCTGCTTGCTTCGGGTGG
CAGCGGAATGCCGGCGCTGCAAGCAATGTTGTCCAGGCAGGTGGATGAGGAACGCCAGGGGCAGCTGCAAG
GCTCACTGGCGGCGCTCACCAGCCTGACCTCGATCGTCGGACCCCTCCTCTTCACGGCGATCTATGCGGCTTCT
ATAACAACGTGGAACGGGTGGGCATGGATTGCAGGCGCTGCCCTCTACTTGCTCTGCCTGCCGGCGCTGCGTC
GCGGGCTTTGGAGCGGCGCAGGGCAACGAGCCGATCGCTGATCGTGGAAACGATAGGCCTATGCCATGCGG
GTCAAGGCGACTTCCGGCAAGCTATACGCGCCCTAGGAGTGCGGTTGGAACGTTGGCCCAGCCAGACACTCCC
GATCACGAGCAGGACGCCGATGATTTGAAGCGCACTCAGCGTCTGATCCAAGAACAACCATCCTAGCAACAGC
GCGGCGACGGCGGTCCCCGGGCTGAGAAAGCCCAGTAAGGAAACAACTGTAGGTTCGAGTCGCGAGATCCCC
CGGAACCAAAGGAAGTATGGTTAAACCCGCTCCGATCAGGCCGAGCCACGCCAGGCCGAGAACATTGGTTCC
TGTAGGCATCGGGATTGGCGGATCAAACACTAAAGCTACTGGAACGAGCAGAAGTCCTCCGGCCGCCAGTTG
CAGGTAAAGGTGAGCAGAGGCACGGGAGGTTGCCACTGCGGGTCAGCACGGTTCCGAACGCCATGGAAAC
CGCCCCCGCAGGCCCGCTGCGACGCCGACAGGATCTAGCGCTGCGTTTGGTGTCAACACCAACAGCGCCACGC
CCGCAGTTCCGCAGATAGCCCCAGGACCGCCATCAATCGTATCGGGCTACCTAGCAGAGCGGCAGAGATGAA
CACGACCATCAGCGGCTGCACACGCCTACCGTCGCCGCGACCCGCCCGGCAGGCGGTAGACCGAAATAAACA
ACAAGCTCCAGAATAGCGAAATATTAAGTGCGCCGAGGATGAAGATGCGCATCCACCAGATTCCCGTTGGAAT

CTGTCGGACGATCATCACGAGCAATAAACCCGCCGGCAACGCCCGGAGCAGCATACCGGCGACCCCTCGGCCT
CGCTGTTCGGGCTCCACGAAGACGCCGGACATATGCGCCTTGTGAGCGTCCTTGGGGCCGTCCTCCTGTTTCAA
GACCGACAGCCCAATGATCTCGCCGTCGATGTAGGCGCCGAATGCCACGGCATCTCGCAACCGTTCAGCGAAC
GCCTCCATGGGCTTTTTCTCCTCGTGCTCGTAAACGGACCCGAACATCTCTGGAGCTTTCTTCAGGGCCGACAA
TCGGATCTCGCGGAAATCCTGCACGTCGGCCGCTCCAAGCCGTCGAATCTGAGCCTTAATCACAATTGTCAATT
TTAATCCTCTGTTTATCGGCAGTTCGTAGAGCGCGCCGTGCGTCCCGAGCGATACTGAGCGAAGCAAGTGCGT
CGAGCAGTGCCCGCTTGTTCCTGAAATGCCAGTAAAGCGCTGGCTGCTGAACCCCCAGCCGGAACTGACCCCA
CAAGGCCCTAGCGTTTGCAATGCACCAGGTCATCATTGACCCAGGCGTGTTCCACCAGGCCGCTGCCTCGCAA
CTCTTCGCAGGCTTCGCCGACCTGCTCGCGCCACTTCTTCACGCGGGTGGAATCCGATCCGCACATGAGGCGG
AAGGTTTCCAGCTTGAGCGGGTACGGCTCCCGGTGCGAGCTGAAATAGTCGAACATCCGTCGGGCCGTCGGC
GACAGCTTGCGGTACTTCTCCCATATGAATTTCGTGTAGTGGTCGCCAGCAAACAGCACGACGATTTCCTCGTC
GATCAGGACCTGGCAACGGGACGTTTTCTTGCCACGGTCCAGGACGCGGAAGCGGTGCAGCAGCGACACCGA
TTCCAGGTGCCCAACGCGGTCGGACGTGAAGCCCATCGCCGTCGCCTGTAGGCGCGACAGGCATTCCTCGGCC
TTCGTGTAATACCGGCCATTGATCGACCAGCCCAGGTCCTGGCAAAGCTCGTAGAACGTGAAGGTGATCGGCT
CGCCGATAGGGGTGCGCTTCGCGTACTCCAACACCTGCTGCCACACCAGTTCGTCATCGTCGGCCCGCAGCTC
GACGCCGGTGTAGGTGATCTTCACGTCCTTGTTGACGTGGAAAATGACCTTGTTTTGCAGCGCCTCGCGCGGG
ATTTTCTTGTTGCGCGTGGTGAACAGGGCAGAGCGGGCCGTGTCGTTTGGCATCGCTCGCATCGTGTCCGGCC
ACGGCGCAATATCGAACAAGGAAAGCTGCATTTCCTTGATCTGCTGCTTCGTGTGTTTCAGCAACGCGGCCTGC
TTGGCCTCGCTGACCTGTTTTGCCAGGTCCTCGCCGGCGGTTTTTCGCTTCTTGGTCGTCATAGTTCCTCGCGTG
TCGATGGTCATCGACTTCGCCAAACCTGCCGCCTCCTGTTCGAGACGACGCGAACGCTCCACGGCGGCCGATG
GCGCGGGCAGGGCAGGGGGAGCCAGTTGCACGCTGTCGCGCTCGATCTTGGCCGTAGCTTGCTGGACCATCG
AGCCGACGGACTGGAAGGTTTCGCGGGGCGCACGCATGACGGTGCGGCTTGCGATGGTTTCGGCATCCTCGG
CGGAAAACCCCGCGTCGATCAGTTCTTGCCTGTATGCCTTCCGGTCAAACGTCCGATTCATTCACCCTCCTTGCG
GGATTGCCCCGACTCACGCCGGGGCAATGTGCCCTTATTCCTGATTTGACCCGCCTGGTGCCTTGGTGTCCAGA
TAATCCACCTTATCGGCAATGAAGTCGGTCCCGTAGACCGTCTGGCCGTCCTTCTCGTACTTGGTATTCCGAATC
TTGCCCTGCACGAATACCAGCGACCCCTTGCCCAAATACTTGCCGTGGGCCTCGGCCTGAGAGCCAAAACACTT
GATGCGGAAGAAGTCGGTGCGCTCCTGCTTGTCGCCGGCATCGTTGCGCCACTCTTCATTAACCGCTATATCGA
AAATTGCTTGCGGCTTGTTAGAATTGCCATGACGTACCTCGGTGTCACGGGTAAGATTACCGATAAACTGGAA
CTGATTATGGCTCATATCGAAAGTCTCCTTGAGAAAGGAGACTCTACTTTAGCTAAACATTGGTTCCGCTGTCA
AGAACTTTAGCGGCTAAAATTTTGCGGGCCGCGACCAAAGGTGCGAGGGGCGGCTTCCGCTGTGTACAACCA
GATATTTTTCACCAACATCCTTCGTCGCTCGATGAGCGGGCATGACGAAACATGAGCTGTCGGAGAGGGCAGG
GGTTTCAATTTCGTTTTTATCAGACTTAACCAACGGTAAGGCCAACCCCTCGTTGAAGGTGATGGAGGCCATTG
CCGACGCCCTGGAAACTCCCCTACCTCTTCTCCTGGAGTCCACCGACCTTGACCGCGAGGCACTCGCGGAGATT
GCGGGTCATCCTTTCAAGAGCAGCGTGCCGCCCGGATACGAACGCATCAGTGTGGTTTTGCCGTCACATAAGG
CGTTTATCGTAAAGAAATGGGGCGACGACACCCGAAAAAAGCTGCGTGGAAGGCTCTGACGCCAAGGGTTAG
GGCTTGCACTTCCTTCTTTAGCCGCTAAAACGGCCCCTTCTCTGCGGGCCGTCGGCTCGCGCATCATATCGACAT
CCTCAACGGAAGCCGTGCCGCGAATGGCATCGGGCGGGTGCGCTTTGACAGTTGTTTTCTATCAGAACCCCTA
CGTCGTGCGGTTCGATTAGCTGTTTGTCTTGCAGGCTAAACACTTTCGGTATATCGTTTGCCTGTGCGATAATGT
TGCTAATGATTTGTTGCGTAGGGGTTACTGAAAAGTGAGCGGGAAAGAAGAGTTTCAGACCATCAAGGAGCG
GGCCAAGCGCAAGCTGGAACGCGACATGGGTGCGGACCTGTTGGCCGCGCTCAACGACCCGAAAACCGTTGA
AGTCATGCTCAACGCGGACGGCAAGGTGTGGCACGAACGCCTTGGCGAGCCGATGCGGTACATCTGCGACAT
GCGGCCCAGCCAGTCGCAGGCGATTATAGAAACGGTGGCCGGATTCCACGGCAAAGAGGTCACGCGGCATTC
GCCCATCCTGGAAGGCGAGTTCCCCTTGGATGGCAGCCGCTTTGCCGGCCAATTGCCGCCGGTCGTGGCCGCG
CCAACCTTTGCGATCCGCAAGCGCGCGGTCGCCATCTTCACGCTGGAACAGTACGTCGAGGCGGGCATCATGA
CCCGCGAGCAATACGAGGTCATTAAAAGCGCCGTGATTGATGATATAGCGGCCCGGCTGCTCCTGGTTCTCGC
GCACCGAAATGGGTGACTTCACCCCGCGCTCTTTGATCGTGGCACCGATTTCCGCGATGCTCTCCGGGGAAAA
GCCGGGGTTGTCGGCCGTCCGCGGCTGATGCGGATCTTCGTCGATCAGGTCCAGGTCCAGCTCGATAGGGCC
GGAACCGCCCTGAGACGCCGCAGGAGCGTCCAGGAGGCTCGACAGGTCGCCGATGCTATCCAACCCCAGGCC
GGACGGCTGCGCCGCGCCTGCGGCTTCCTGAGCGGCCGCAGCGGTGTTTTTCTTGGTGGTCTTGGCTTGAGCC
GCAGTCATTGGGAAATCTCCATCTTCGTGAACACGTAATCAGCCAGGGCGCGAACCTCTTTCGATGCCTTGCGC
GCGGCCGTTTTCTTGATCTTCCAGACCGGCACACCGGATGCGAGGGCATCGGCGATGCTGCTGCGCAGGCCAA
CGGTGGCCGGAATCATCATCTTGGGGTACGCGGCCAGCAGCTCGGCTTGGTGGCGCGCGTGGCGCGGATTCC

GCGCATCGACCTTGCTGGGCACCATGCCAAGGAATTGCAGCTTGGCGTTCTTCTGGCGCACGTTCGCAATGGT
CGTGACCATCTTCTTGATGCCCTGGATGCTGTACGCCTCAAGCTCGATGGGGGACAGCACATAGTCGGCCGCG
AAGAGGGCGGCCGCCAGGCCGACGCCAAGGGTCGGGGCCGTGTCGATCAGGCACACGTCGAAGCCTTGGTT
CGCCAGGGCCTTGATGTTCGCCCCGAACAGCTCGCGGGCGTCGTCCAGCGACAGCCGTTCGGCGTTCGCCAGT
ACCGGGTTGGACTCGATGAGGGCGAGGCGCGCGGCCTGGCCGTCGCCGGCTGCGGGTGCGGTTTCGGTCCA
GCCGCCGGCAGGGACAGCGCCGAACAGCTTGCTTGCATGCAGGCCGGTAGCAAAGTCCTTGAGCGTGTAGGA
CGCATTGCCCTGGGGGTCCAGGTCGATCACGGCAACCCGCAAGCCGCGCTCGAAAAAGTCGAAGGCAAGATG
CACAAGGGTCGAAGTCTTGCCGACGCCGCCTTTCTGGTTGGCCGTGACCAAAGTTTTCATCGTTTGGTTTCCTG
TTTTTTCTTGGCGTCCGCTTCCCACTTCCGGACGATGTACGCCTGATGTTCCGGCAGAACCGCCGTTACCCGCGC
GTACCCCTCGGGCAAGTTCTTGTCCTCGAACGCGGCCCACACGCGATGCACCGCTTGCGACACTGCGCCCCTG
GTCAGTCCCAGCGACGTTGCGAACGTCGCCTGTGGCTTCCCATCGACTAAGACGCCCCGCGCTATCTCGATGGT
CTGCTGCCCCACTTCCAGCCCCTGGATCGCCTCCTGGAACTGGCTTTCGGTAAGCCGTTTCTTCATGGATAACAC
CCATAATTTGCTCCGCGCCTTGGTTGAACATAGCGGTGACAGCCGCCAGCACATGAGAGAAGTTTAGCTAAAC
ATTTCTCGCACGTCAACACCTTTAGCCGCTAAAACTCGTCCTTGGCGTAACAAAACAAAAGCCCGGAAACCGGG
CTTCGTCTCTTGCCGCTTATGGCTCTGCACCCGGCTCCATCACCAACAGGTCGCGCACGCGCTTCACTCGGTTGC
GGATCGACACTGCCAGCCCAACAAAGCCGGTTGCCGCCGCCGCCAGGATCGCGCCGATGATGCCGGCCACAC
CGGCCATCGCCCACCAGGTCGCCGCCTTCCGGTTCCATTCCTGCTGGTACTGCTTCGCAATGCTGGACCTCGGC
TCACCATAGGCTGACCGCTCGATGGCGTATGCCGCTTCTCCCCTTGGCGTAAAACCCAGCGCCGCAGGCGGCA
TTGCCATGCTGCCCGCCGCTTTCCCGACCACGACGCGCGCACCAGGCTTGCGGTCCAGACCTTCGGCCACGGC
GAGCTGCGCAAGGACATAATCAGCCGCCGACTTGGCTCCACGCGCCTCGATCAGCTCTTGCACTCCGCGAAAT
CCTTGCCTCCACGGCCGCCATGAATCGCGCACGCGGCGAAGGCTCCGCAGGGCCGGCGTCGTGATCGCCGCC
GAGAATGCCCTTCACCAAGTTCGACGACACGAAAATCATGCTGACGGCTATCACCATCATGCAGACGATCGCA
CGAACCCGCTGAATTAATTCACCCCCGAACACGAGCACGGCACCCGCGACCACTATGCCAAGAATGCCCAAGG
TAAAAATTGCCGGCCCCGCCATGAAGTCCGTGAATGCCCCGACGGCCGAAGTGAAGGGCAGGCCGCCACCCA
GGCCGCCGCCCTCACTGCCCGGCACCTGGTCGCTGAATGTCGATGCCAGCACCTGCGGCACGTCAATGCTTCC
GGGCGTCGCGCTCGGGCTGATCGCCCATCCCGTTACTGCCCCGATCCCGGCAATGGCAAGGACTGCCAGCGCT
GCCATTTTTGGGGTGAGGTCGTTCGCGGCCGAGGGGCGCAGCCCCTGGGGGGATGGGGTGCCGCGTTAGCG
GGCCGGGAGGGTTCGAGAAGGGGGGGCACCCCCCTTCGGCGTGCGCGGTCACGCGCCAGGGCGCAGCCCTG
GTTAAAAACAAGGTTTATAAATATTGGTTTAAAAGCAGGTTAAAAGACAGGTTAGCGGTGGCCGAAAAACGG
GCGGAAACCCTTGCAAATGCTGGATTTTCTGCCTGTGGACAGCCCCTCAAATGTCAATAGGTGCGCCCCTCATC
TGTCAGCACTCTGCCCCTCAAGTGTCAAGGATCGCGCCCCTCATCTGTCAGTAGTCGCGCCCCTCAAGTGTCAA
TACCGCAGGGCACTTATCCCCAGGCTTGTCCACATCATCTGTGGGAAACTCGCGTAAAATCAGGCGTTTTCGCC
GATTTGCGAGGCTGGCCAGCTCCACGTCGCCGGCCGAAATCGAGCCTGCCCCTCATCTGTCAACGCCGCGCCG
GGTGAGTCGGCCCCTCAAGTGTCAACGTCCGCCCCTCATCTGTCAGTGAGGGCCAAGTTTTCCGCGAGGTATC
CACAACGCCGGCGGCCGCGGTGTCTCGCACACGGCTTCGACGGCGTTTCTGGCGCGTTTGCAGGGCCATAGAC
GGCCGCCAGCCCAGCGGCGAGGGCAACCAGCCCGGTGAGCGTCGGAAAGGCGCTGGAAGCCCCGTAGCGAC
GCGGAGAGGGGCGAGACAAGCCAAGGGCGCAGGCTCGAGTCGCAGCACGACATAGCCGGTTCTCGCAAGGA
CGAGAATTTCCCTGCGGTGCCCCTCAAGTGTCAATGAAAGTTTCCAACGCGAGCCATTCGCGAGAGCCTTGAG
SceI LacI
TCCACGCTAGATCTGAATTGTAGGGATAACAGGGTAATGAATTGAGCCAGCACAATTGTGAGCGGATAACAAT
LacI LexA$_{BT}$
TAAAAGTCAGAAATTGTGAGCGGATAACAATTAAAGACGGAGCATATGCAAATCGAATAAATGTTCGATTTAA
LexA$_{BT}$ LacI
ATCGAATAAATGTTCGATTTTATGGTACCCGCGCAAATTGTGAGCGGATAACAATTAGCAAGACAGAATTGTG
LacI SceI
AGCGGATAACAATTCTGGGAATTCTAGGGATAACAGGGTAATACTTCGG

>Complete sequence of the pRW-LexA-mCherry plasmid. Features of the plasmid are shown in different colours: LexA (*E. coli*) promoter and terminator in purple, *lexA*$_{BT}$ fused to *mCherry* in green and red, recognition sites for the yeast meganuclease I-SceI in orange, binding sites for the LacI repressor in blue and binding sites for the LexA$_{BT}$ repressor in pale orange.

LexA (*E. coli*) promoter

GATCCGTCGACCTGCAGCCAAGCTTACCCTTCCAGAATTCGATAAATCTCTGGTTTATTGTGCAGTTTATGGTTC
CAAAATCGCCTTTTGCTGTATATACTCACAGCATAACTGTATATACACCCAGGGGGCGGAATGTTAGAAAACAT

lexA$_{BT}$

GGAAAAGTTAACGAAACGCCAGCAAGACATTCTCGACTTTATTAAGCTAAAAGTACAAGAAAAAGGATATCCA
CCTTCCGTACGTGAAATTGGTCAAGCAGTCGGCCTCGCTTCTAGTTCTACAGTGCACGGACATTTATCACGATT
AGAAGAAAAAGGATACATTCGACGCGATCCAACAAAACCACGTGCCATTGAAATTTTAGGCGAAGACCGAAT
GGATACGGAAACACAATCTGTTATTCAAGTTCCAATTGTCGGAAAAGTTACTGATGGTTTACCAATTACAGCGG
TCGAAAGCGTAGAGGAACATTTCCCACTTCCAGCTAGCATTGTTTCCGGAGCAGACCAAGTGTTCATGTTACGT
ATTTCTGGAGATAGTATGATTGAAGCTGGTATTTTCGATGGAGATTTAGTTGTTGTTCGCCAACAACAGTCTGC
ATATAATGGTGAAATTGTAGTCGCTTTAACAGAAGATAACGAAGCAACTGTTAAACGTTTCTATAAAGAAAAA
GACCATTTCCGTCTACAACCAGAAAACTCTTCATTAGAACCTATCATTTTAAAACAAGTGTCAGTTATCGGGAAA

linker mCherry

GTAATTGGCGTATATCGTGATTTACATcccgggGTGAGCAAGGGCGAGGAGGATAACATGGCCATCATCAAGGA
GTTCATGCGCTTCAAGGTTCACATGGAGGGCTCCGTGAACGGCCACGAGTTCGAGATCGAGGGCGAGGGCGA
GGGCCGCCCCTACGAGGGCACCCAGACCGCCAAGCTGAAGGTGACCAAGGGTGGCCCCCTGCCCTTCGCCTG
GGACATCCTGTCCCCTCAGTTCATGTACGGCTCCAAGGCCTACGTGAAGCACCCCGCCGACATCCCCGACTACT
TGAAGCTGTCCTTCCCCGAGGGCTTCAAGTGGGAGCGCGTGATGAACTTCGAGGACGGCGGCGTGGTGACCG
TGACCCAGGACTCCTCCCTGCAAGACGGCGAGTTCATCTACAAGGTGAAGCTGCGCGGCACCAACTTCCCCTC
CGACGGCCCCGTAATGCAGAAGAAGACTATGGGCTGGGAGGCCTCCTCCGAGCGGATGTACCCCGAGGACG
GCGCGCTGAAGGGCGAGATCAAGCAGAGGCTGAAGCTGAAGGACGGCGGCCACTACGACGCTGAGGTCAAG
ACCACCTACAAGGCCAAGAAGCCCGTGCAACTGCCCGGCGCGTACAACGTCAACATCAAGTTGGACATCACCT
CCCACAACGAGGACTACACCATCGTGGAACAGTACGAACGCGCCGAGGGCCGCCACTCCACCGGCGGCATGG

terminator

ACGAGCTGTATAAGTAACCGATTACGTGCCTGGCGGCAGTAGCGCGGTGGTCCCACCTGACCCCATGCCGAAC
TCAGAAGTGAAACGCCGTAGCGCCGATGGTAGTGTGGGGTCTCCCCATGCGAGAGTAGGGAACTGCCAGGCA
TCAAATAAAACGAAAGGCTCAGTCGAAAGACTGGGCCTTTCGAATTTGGTCGCGCGCGGATGTTTGGCTGTG
TTGGCTGGGCGCTGTGTGCCTCGATTGTCGGCATCATGTTCACCATCAATAATCAGTTTGTTTTCTGGCTGGGC
TCTGGCTGTGCACTCATCCTCGCCGTTTTACTCTTTTTCGCCAAAACGGATGCGCCCTCTTCTGCCACGGTTGCC
AATGCGGTAGGTGCCAACCATTCGGCATTTAGCCTTAAGCTGGCACTGGAACTGTTCAGACAGCCAAAACTGT
GGTTTTTGTCACTGTATGTTATTGGCGTTTCCTGCACCTACGATGTTTTTGACCAACAGTTTGCTAATTTCTTTAC
TTCGTTCTTTGCTACCGGTGAACAGGGTACGCGGGTATTTGGCTACGTAACGACAATGGGCGAATTACTTAAC
GCCTCGATTATGTTCTTTGCGCCACTGATCATTAATCGCATCGGTGGGAAAAACGCCCTGCTGCTGGCTGGCAC
TATTATGTCTGTACGTATTATTGGCTCATCGTTCGCCACCTCAGCGCTGGAAGTGGTTATTCTGAAAACGCTGCA
TATGTTTGAAGTACCGTTCCTGCTGGTGGGCTGCTTTAAATATATTACCAGCCAGTTTGAAGTGCGTTTTTCAGC
GACGATTTATCTGGTCTGTTTCTGCTTCTTTAAGCAACTGGCGATGATTTTTATGTCTGTACTGGCGGGCAATAT
GTATGAAAGCATCGGTTTCCAGGGCGCTTATCTGGTGCTGGGTCTGGTGGCGCTGGGCTTCACCTTAATTTCCG
TGTTCACGCTTAGCGGCCCCGGCCCGCTTTCCCTGCTGCGTCGTCAGGTGAATGAAGTCGCTTAAGCAATCAAT
GTCGGATGCGGCGCGACGCTTATCCGACCAACATATCATAACGGAGTGATCGCATTGAACATGCCAATGACCG
AAAGAATAAGAGCAGGCAAGCTATTTACCGATATGTGCGAAGGCTTACCGGAAAAAAGACTTCGTGGGAAAA
CGTTAATGTATGAGTTTAATCACTCGCATCCATCAGAAGTTGAAAAAAGAGAAAGCCTGATTAAAGAAATGTTT
GCCACGGTAGGGGAAAACGCCTGGGTAGAACCGCCTGTCTATTTCTCTTACGGTTCCAACATCCATATAGGCC
GCAATTTTTATGCAAATTTCAATTTAACCATTGTCGATGACTACACGGTAACAATCGGTGATAACGTACTGATTG
CACCCAACGTTACTCTTTCCGTTACGGGACACCCTGTACACCATGAATTGAGAAAAAACGGCGAGATGTACTCT
TTTCCGATAACGATTGGCAATAACGTCTGGATCGGAAGTCATGTGGTTATTAATCCAGGCGTCACCATCGGGG
ATAATTCTGTTATTGGCGCGGGTAGTATCGTCACAAAAGACATTCCACCAAACGTCGTGGCGGCTGGCGTTCCT
TGTCGGGTTATTCGCGAAATAAACGACCGGGATAAGCACTATTATTTCAAAGATTATAAAGTTGAATCGTCAGT

TTAAATTATAAAAATTGCCTGATACGCTGCGCTTATCAGGCCTACAAGTTCAGCGATCTACATTAGCCGCATCC
GGCATGAACAAAGCGCAGGAACAAGCGTCGCATCATGCCTCTTTGACCCACAGCTGCGGAAAACGTACTGGT
GCAAAACGCAGGGTTATGATCATCAGCCCAACGACGCACAGCGCATGAAATGCCCAGTCCATCAGGTAATTGC
CGCTGATACTACGCAGCACGCCAGAAAACCACGGGGCAAGCCCGGCGATGATAAAACCGATTCCCTGCATAA
ACGCCACCAGCTTGCCAGCAATAGCCGGTTGCACAGAGTGATCGAGCGCCAGCAGCAAACAGAGCGGAAACG
CGCCGCCCAGACCTAACCCACACACCATCGCCCACAATACCGGCAATTGCATCGGCAGCCAGATAAAGCCGCA
GAACCCCACCAGTTGTAACACCAGCGCCAGCATTAACAGTTTGCGCCGATCCTGATGGCGAGCCATAGCAGGC
ATCAGCAAAGCTCCTGCGGCTTGCCCAAGCGTCATCAATGCCAGTAAGGAACCGCTGTACTGCGCGCTGGCAC
CAATCTCAATATAGAAAGCGGGTTACCATGGGCGGGCTGGCGCTGGCCGGAAGCTGGCTTCATCAGCATGTC
AGCGAACGTCGCAGTGTCGCCATCAGTCTGTTGCTGATTGCCGTCGGTGCATTGATGCGTGAGCTTTACCCGCA
AAGTGCGCTGCTGCTTAGCAGCGCACTGCTTGGTGGGGTGGGGATCGGCATCATTCAGGCGGTGATGCCTTC
GGTGATTAAACGGCGGTTTCAGCAGCGCACGCCACTGGTGATGGGGCTGTGGTCCGCGGCTCTGATGGGCGG
CGGTGGGCTTGGTGCCGCCATAACGCCCTGGTTAGTTCAACATAGCGAAACCTGGTATCAAACACTCGCCTGG
TGGGCGCTGCCTGCCGTTGTTGCGCTCTTTGCCTGGTGGTGGCAAAGCGCCCGCGAGGTCGCCTCTTCCCACA
AGACAACAACCACTCCGGTTCGCGTGGTATTCACTCCCCGCGCGTGGACGCTGGGTGTTTACTTCGGTCTGATT
AACGGCGGTTACGCCAGCCTGATTGCCTGGTTACCGCCAAGCTGCAGCCAAGCTAGCTTTTGCCATTCTCACCG
GATTCAGTCGTCACTCATGGTGATTTCTCACTTGATAACCTTATTTTTGACGAGGGGAAATTAATAGGTTGTATT
GATGTTGGACGAGTCGGAATCGCAGACCGATACCAGGATCTTGCCATCCTATGGAACTGCCTCGGTGAGTTTT
CTCCTTCATTACAGAAACGGCTTTTTCAAAAATATGGTATTGATAATCCTGATATGAATAAATTGCAGTTTCATT
TGATGCTGGATGAGTTTTTCTAATCAGAATTGGTTAATTGGTTGTAACACTGGCAGAGCATTACGCTGACTTGA
CGGGACGGCGGCTTTGTTGAATAAATCGAACTTTTGCTGAGTTGAAGGATCAGATCACGCATCTTCCCGACAA
CGCAGACCGTTCCGTGGCAAAGCAAAAGTTCAAAATCACCAACTGGTCCACCTACAACAAAGCTCTCATCAACC
GTGGCTCCCTCACTTTCTGGCTGGATGATGGGGCGATTCAGGCCTGGTATGAGTCAGCAACACCTTCTTCACGA
GGCAGACCTCAGCGCCAGAAGGCCGCCAGAGAGGCCGAGCGCGGCGTGAGGCTTGGACGCTAGGGCAGGG
CATGAAAAAGCCCGTAGCGGGCGCTACGGGGCTCTGACGCGGTGGAAAGGGGGAGGGGATGTGTTGTCTAC
ATGGCTCTGCTGTAGTGAGTGGGTTGCGCTCCGGCAGCGGTCCTGATCAATCGTCACCCTTTCTCGGTCCTTCA
ACGTTCCTGACAACGAGCCTCCTTTTCGCCAATCCATCGACAATCACCGCGAGTCCCTGCTCGAACGCTGCGTC
CGGACCGGCTTCGTCGAAGGCGTCTATCGCGGCCCGCAACAGCGGCGGAGAGCGGAGCCTGTTCAACGGTGC
CGCCGCGCTCGCCGGACTCGCTGTCGCCGGCCTGCTCCTCAAGCACGGCCCCAACAGTGAAGTAGCTGATTGT
CATCAGCGCATTGACGGCGTCCCCGGCCGAAAAACCCGCCTCGCAGAGGAAGCGAAGCTGCGCGTCGGCCGT
TTCCATCTGCGGTGCGCCCGGTCGCGTGCCGGCATGGATGCGCGCGCCATCGCGGTAGGCGAGCAGCGCCTG
CCTGAAGCTGCGGGCATTCCCAGTCAGAAATGAGCGCCAGTCGTCGTCGGCTCTCGGCACCGAAGTGCTATGA
TTCTCCGCCAGCATGGCTTCGGCCAGTGCGTCGAGCAGCGCCCGCTTGTTCCTGAAGTGCCAGTAAAGCGCCG
GCTGCTGAACCCCCAACCGTTCCGCCAGTTTGCGTGTCGTCAGACCGTCTACGCCGACCTCGTTCAACAGGTCT
AGGGCGGCACGGATCACTGTATTCGGCTGCAACTTTGTCATGCTTGACACTTTATCACTGATAAACATAATATG
TCCACCAACTTATCAGTGATAAAGAATCCGCGCGTTCAATCGGACCAGCGGAGGCTGGTCCGGAGGCCAGAC
GTGAAACCCAACATACCCCTGATCGTAATTCTGAGCACTGTCGCGCTCGACGCTGTCGGCATCGGCCTGATTAT
GCCGGTGCTGCCGGGCCTCCTGCGCGATCTGGTTCACTCGAACGACGTCACCGCCCACTATGGCATTCTGCTG
GCGCTGTATGCGTTGGTGCAATTTGCCTGCGCACCTGTGCTGGGCGCGCTGTCGGATCGTTTCGGGCGGCGGC
CAATCTTGCTCGTCTCGCTGGCCGGCGCCACTGTCGACTACGCCATCATGGCGACAGCGCCTTTCCTTTGGGTT
CTCTATATCGGGCGGATCGTGGCCGGCATCACCGGGGCGACTGGGGCGGTAGCCGGCGCTTATATTGCCGAT
ATCACTGATGGCGATGAGCGCGCGCGGCACTTCGGCTTCATGAGCGCCTGTTTCGGGTTCGGGATGGTCGCG
GGACCTGTGCTCGGTGGGCTGATGGGCGGTTTCTCCCCCCACGCTCCGTTCTTCGCCGCGGCAGCCTTGAACG
GCCTCAATTTCCTGACGGGCTGTTTCCTTTTGCCGGAGTCGCACAAAGGCGAACGCCGGCCGTTACGCCGGGA
GGCTCTCAACCCGCTCAGCTTCGTTCGGTGGGCCCGGGGCATGACCGTCGTCGCCGCCCTGATGGCGGTCTTC
TTCATCATGCAACTTGTCGGACAGGTGCCGGCCGCGCTTTGGGTCATTTTCGGCGAGGATCGCTTTCACTGGG
ACGCGACCACGATCGGCATTTCGCTTGCCGCATTTGGCATTCTGCATTCACTCGCCCAGGCAATGATCACCGGC
CCTGTAGCCGCCCGGCTCGGCGAAAGGCGGGCACTCATGCTCGGAATGATTGCCGACGGCACAGGCTACATC
CTGCTTGCCTTCGCGACACGGGGATGGATGGCGTTCCCGATCATGGTCCTGCTTGCTTCGGGTGGCAGCGGAA
TGCCGGCGCTGCAAGCAATGTTGTCCAGGCAGGTGGATGAGGAACGCCAGGGGCAGCTGCAAGGCTCACTG
GCGGCGCTCACCAGCCTGACCTCGATCGTCGGACCCCTCCTCTTCACGGCGATCTATGCGGCTTCTATAACAAC
GTGGAACGGGTGGGCATGGATTGCAGGCGCTGCCCTCTACTTGCTCTGCCTGCCGGCGCTGCGTCGCGGGCTT

TGGAGCGGCGCAGGGCAACGAGCCGATCGCTGATCGTGGAAACGATAGGCCTATGCCATGCGGGTCAAGGC
GACTTCCGGCAAGCTATACGCGCCCTAGGAGTGCGGTTGGAACGTTGGCCCAGCCAGACACTCCCGATCACGA
GCAGGACGCCGATGATTTGAAGCGCACTCAGCGTCTGATCCAAGAACAACCATCCTAGCAACAGCGCGGCGA
CGGCGGTCCCCGGGCTGAGAAAGCCCAGTAAGGAAACAACTGTAGGTTCGAGTCGCGAGATCCCCCGGAACC
AAAGGAAGTATGGTTAAACCCGCTCCGATCAGGCCGAGCCACGCCAGGCCGAGAACATTGGTTCCTGTAGGC
ATCGGGATTGGCGGATCAAACACTAAAGCTACTGGAACGAGCAGAAGTCCTCCGGCCGCCAGTTGCAGGTAA
AGGTGAGCAGAGGCACGGGAGGTTGCCACTGCGGGTCAGCACGGTTCCGAACGCCATGGGAAACCGCCCCC
GCAGGCCCGCTGCGACGCCGACAGGATCTAGCGCTGCGTTTGGTGTCAACACCAACAGCGCCACGCCCGCAG
TTCCGCAGATAGCCCCAGGACCGCCATCAATCGTATCGGGCTACCTAGCAGAGCGGCAGAGATGAACACGACC
ATCAGCGGCTGCACACGCCTACCGTCGCCGCGACCCGCCCGGCAGGCGGTAGACCGAAATAAACAACAAGCT
CCAGAATAGCGAAATATTAAGTGCGCCGAGGATGAAGATGCGCATCCACCAGATTCCCGTTGGAATCTGTCGG
ACGATCATCACGAGCAATAAACCCGCCGGCAACGCCCGGAGCAGCATACCGGCGACCCCTCGGCCTCGCTGTT
CGGGCTCCACGAAGACGCCGGACATATGCGCCTTGTGAGCGTCCTTGGGGCCGTCCTCCTGTTTCAAGACCGA
CAGCCCAATGATCTCGCCGTCGATGTAGGCGCCGAATGCCACGGCATCTCGCAACCGTTCAGCGAACGCCTCC
ATGGGCTTTTTCTCCTCGTGCTCGTAAACGGACCCGAACATCTCTGGAGCTTTCTTCAGGGCCGACAATCGGAT
CTCGCGGAAATCCTGCACGTCGGCCGCTCCAAGCCGTCGAATCTGAGCCTTAATCACAATTGTCAATTTTAATC
CTCTGTTTATCGGCAGTTCGTAGAGCGCGCCGTGCGTCCCGAGCGATACTGAGCGAAGCAAGTGCGTCGAGCA
GTGCCCGCTTGTTCCTGAAATGCCAGTAAAGCGCTGGCTGCTGAACCCCCAGCCGGAACTGACCCCACAAGGC
CCTAGCGTTTGCAATGCACCAGGTCATCATTGACCCAGGCGTGTTCCACCAGGCCGCTGCCTCGCAACTCTTCG
CAGGCTTCGCCGACCTGCTCGCGCCACTTCTTCACGCGGGTGGAATCCGATCCGCACATGAGGCGGAAGGTTT
CCAGCTTGAGCGGGTACGGCTCCCGGTGCGAGCTGAAATAGTCGAACATCCGTCGGGCCGTCGGCGACAGCT
TGCGGTACTTCTCCCATATGAATTTCGTGTAGTGGTCGCCAGCAAACAGCACGACGATTTCCTCGTCGATCAGG
ACCTGGCAACGGGACGTTTTCTTGCCACGGTCCAGGACGCGGAAGCGGTGCAGCAGCGACACCGATTCCAGG
TGCCCAACGCGGTCGGACGTGAAGCCCATCGCCGTCGCCTGTAGGCGCGACAGGCATTCCTCGGCCTTCGTGT
AATACCGGCCATTGATCGACCAGCCCAGGTCCTGGCAAAGCTCGTAGAACGTGAAGGTGATCGGCTCGCCGAT
AGGGGTGCGCTTCGCGTACTCCAACACCTGCTGCCACACCAGTTCGTCATCGTCGGCCCGCAGCTCGACGCCG
GTGTAGGTGATCTTCACGTCCTTGTTGACGTGGAAAATGACCTTGTTTTGCAGCGCCTCGCGCGGGATTTTCTT
GTTGCGCGTGGTGAACAGGGCAGAGCGGGCCGTGTCGTTTGGCATCGCTCGCATCGTGTCCGGCCACGGCGC
AATATCGAACAAGGAAAGCTGCATTTCCTTGATCTGCTGCTTCGTGTGTTTCAGCAACGCGGCCTGCTTGGCCT
CGCTGACCTGTTTTGCCAGGTCCTCGCCGGCGGTTTTTCGCTTCTTGGTCGTCATAGTTCCTCGCGTGTCGATGG
TCATCGACTTCGCCAAACCTGCCGCCTCCTGTTCGAGACGACGCGAACGCTCCACGGCGGCCGATGGCGCGGG
CAGGGCAGGGGGAGCCAGTTGCACGCTGTCGCGCTCGATCTTGGCCGTAGCTTGCTGGACCATCGAGCCGAC
GGACTGGAAGGTTTCGCGGGGCGCACGCATGACGGTGCGGCTTGCGATGGTTTCGGCATCCTCGGCGGAAAA
CCCCGCGTCGATCAGTTCTTGCCTGTATGCCTTCCGGTCAAACGTCCGATTCATTCACCCTCCTTGCGGGATTGC
CCCGACTCACGCCGGGGCAATGTGCCCTTATTCCTGATTTGACCCGCCTGGTGCCTTGGTGTCCAGATAATCCA
CCTTATCGGCAATGAAGTCGGTCCCGTAGACCGTCTGGCCGTCCTTCTCGTACTTGGTATTCCGAATCTTGCCCT
GCACGAATACCAGCGACCCCTTGCCCAAATACTTGCCGTGGGCCTCGGCCTGAGAGCCAAAACACTTGATGCG
GAAGAAGTCGGTGCGCTCCTGCTTGTCGCCGGCATCGTTGCGCCACTCTTCATTAACCGCTATATCGAAAATTG
CTTGCGGCTTGTTAGAATTGCCATGACGTACCTCGGTGTCACGGGTAAGATTACCGATAAACTGGAACTGATTA
TGGCTCATATCGAAAGTCTCCTTGAGAAAGGAGACTCTACTTTAGCTAAACATTGGTTCCGCTGTCAAGAACTT
TAGCGGCTAAAATTTTGCGGGCCGCGACCAAAGGTGCGAGGGGCGGCTTCCGCTGTGTACAACCAGATATTTT
TCACCAACATCCTTCGTCGCTCGATGAGCGGGCATGACGAAACATGAGCTGTCGGAGAGGGCAGGGGTTTCA
ATTTCGTTTTTATCAGACTTAACCAACGGTAAGGCCAACCCCTCGTTGAAGGTGATGGAGGCCATTGCCGACGC
CCTGGAAACTCCCCTACCTCTTCTCCTGGAGTCCACCGACCTTGACCGCGAGGCACTCGCGGAGATTGCGGGTC
ATCCTTTCAAGAGCAGCGTGCCGCCCGGATACGAACGCATCAGTGTGGTTTTGCCGTCACATAAGGCGTTTATC
GTAAAGAAATGGGGCGACGACACCCGAAAAAAGCTGCGTGGAAGGCTCTGACGCCAAGGGTTAGGGCTTGC
ACTTCCTTCTTTAGCCGCTAAAACGGCCCCTTCTCTGCGGGCCGTCGGCTCGCGCATCATATCGACATCCTCAAC
GGAAGCCGTGCCGCGAATGGCATCGGGCGGGTGCGCTTTGACAGTTGTTTTCTATCAGAACCCCTACGTCGTG
CGGTTCGATTAGCTGTTTGTCTTGCAGGCTAAACACTTTCGGTATATCGTTTGCCTGTGCGATAATGTTGCTAAT
GATTTGTTGCGTAGGGGTTACTGAAAAGTGAGCGGGAAAGAAGAGTTTCAGACCATCAAGGAGCGGGCCAA
GCGCAAGCTGGAACGCGACATGGGTGCGGACCTGTTGGCCGCGCTCAACGACCCGAAAACCGTTGAAGTCAT
GCTCAACGCGGACGGCAAGGTGTGGCACGAACGCCTTGGCGAGCCGATGCGGTACATCTGCGACATGCGGCC

CAGCCAGTCGCAGGCGATTATAGAAACGGTGGCCGGATTCCACGGCAAAGAGGTCACGCGGCATTCGCCCAT
CCTGGAAGGCGAGTTCCCCTTGGATGGCAGCCGCTTTGCCGGCCAATTGCCGCCGGTCGTGGCCGCGCCAACC
TTTGCGATCCGCAAGCGCGCGGTCGCCATCTTCACGCTGGAACAGTACGTCGAGGCGGGCATCATGACCCGCG
AGCAATACGAGGTCATTAAAAGCGCCGTGATTGATGATATAGCGGCCCGGCTGCTCCTGGTTCTCGCGCACCG
AAATGGGTGACTTCACCCCGCGCTCTTTGATCGTGGCACCGATTTCCGCGATGCTCTCCGGGGAAAAGCCGGG
GTTGTCGGCCGTCCGCGGCTGATGCGGATCTTCGTCGATCAGGTCCAGGTCCAGCTCGATAGGGCCGGAACCG
CCCTGAGACGCCGCAGGAGCGTCCAGGAGGCTCGACAGGTCGCCGATGCTATCCAACCCCAGGCCGGACGGC
TGCGCCGCGCCTGCGGCTTCCTGAGCGGCCGCAGCGGTGTTTTTCTTGGTGGTCTTGGCTTGAGCCGCAGTCA
TTGGGAAATCTCCATCTTCGTGAACACGTAATCAGCCAGGGCGCGAACCTCTTTCGATGCCTTGCGCGCGGCC
GTTTTCTTGATCTTCCAGACCGGCACACCGGATGCGAGGGCATCGGCGATGCTGCTGCGCAGGCCAACGGTGG
CCGGAATCATCATCTTGGGGTACGCGGCCAGCAGCTCGGCTTGGTGGCGCGCGTGGCGCGGATTCCGCGCAT
CGACCTTGCTGGGCACCATGCCAAGGAATTGCAGCTTGGCGTTCTTCTGGCGCACGTTCGCAATGGTCGTGAC
CATCTTCTTGATGCCCTGGATGCTGTACGCCTCAAGCTCGATGGGGGACAGCACATAGTCGGCCGCGAAGAGG
GCGGCCGCCAGGCCGACGCCAAGGGTCGGGGCCGTGTCGATCAGGCACACGTCGAAGCCTTGGTTCGCCAGG
GCCTTGATGTTCGCCCCGAACAGCTCGCGGGCGTCGTCCAGCGACAGCCGTTCGGCGTTCGCCAGTACCGGGT
TGGACTCGATGAGGGCGAGGCGCGCGGCCTGGCCGTCGCCGGCTGCGGGTGCGGTTTCGGTCCAGCCGCCG
GCAGGGACAGCGCCGAACAGCTTGCTTGCATGCAGGCCGGTAGCAAAGTCCTTGAGCGTGTAGGACGCATTG
CCCTGGGGGTCCAGGTCGATCACGGCAACCCGCAAGCCGCGCTCGAAAAAGTCGAAGGCAAGATGCACAAGG
GTCGAAGTCTTGCCGACGCCGCCTTTCTGGTTGGCCGTGACCAAAGTTTTCATCGTTTGGTTTCCTGTTTTTTCTT
GGCGTCCGCTTCCCACTTCCGGACGATGTACGCCTGATGTTCCGGCAGAACCGCCGTTACCCGCGCGTACCCCT
CGGGCAAGTTCTTGTCCTCGAACGCGGCCCACACGCGATGCACCGCTTGCGACACTGCGCCCCTGGTCAGTCC
CAGCGACGTTGCGAACGTCGCCTGTGGCTTCCCATCGACTAAGACGCCCCGCGCTATCTCGATGGTCTGCTGCC
CCACTTCCAGCCCCTGGATCGCCTCCTGGAACTGGCTTTCGGTAAGCCGTTTCTTCATGGATAACACCCATAATT
TGCTCCGCGCCTTGGTTGAACATAGCGGTGACAGCCGCCAGCACATGAGAGAAGTTTAGCTAAACATTTCTCG
CACGTCAACACCTTTAGCCGCTAAAACTCGTCCTTGGCGTAACAAAACAAAAGCCCGGAAACCGGGCTTCGTCT
CTTGCCGCTTATGGCTCTGCACCCGGCTCCATCACCAACAGGTCGCGCACGCGCTTCACTCGGTTGCGGATCGA
CACTGCCAGCCCAACAAAGCCGGTTGCCGCCGCCGCCAGGATCGCGCCGATGATGCCGGCCACACCGGCCATC
GCCCACCAGGTCGCCGCCTTCCGGTTCCATTCCTGCTGGTACTGCTTCGCAATGCTGGACCTCGGCTCACCATA
GGCTGACCGCTCGATGGCGTATGCCGCTTCTCCCCTTGGCGTAAAACCCAGCGCCGCAGGCGGCATTGCCATG
CTGCCCGCCGCTTTCCCGACCACGACGCGCGCACCAGGCTTGCGGTCCAGACCTTCGGCCACGGCGAGCTGCG
CAAGGACATAATCAGCCGCCGACTTGGCTCCACGCGCCTCGATCAGCTCTTGCACTCCGCGAAATCCTTGCCTC
CACGGCCGCCATGAATCGCGCACGCGGCGAAGGCTCCGCAGGGCCGGCGTCGTGATCGCCGCCGAGAATGCC
CTTCACCAAGTTCGACGACACGAAAATCATGCTGACGGCTATCACCATCATGCAGACGATCGCACGAACCCGCT
GAATTAATTCACCCCCGAACACGAGCACGGCACCCGCGACCACTATGCCAAGAATGCCCAAGGTAAAAATTGC
CGGCCCCGCCATGAAGTCCGTGAATGCCCCGACGGCCGAAGTGAAGGGCAGGCCGCCACCCAGGCCGCCGCC
CTCACTGCCCGGCACCTGGTCGCTGAATGTCGATGCCAGCACCTGCGGCACGTCAATGCTTCCGGGCGTCGCG
CTCGGGCTGATCGCCCATCCCGTTACTGCCCCGATCCCGGCAATGGCAAGGACTGCCAGCGCTGCCATTTTTGG
GGTGAGGTCGTTCGCGGCCGAGGGGCGCAGCCCCTGGGGGGATGGGGTGCCGCGTTAGCGGGCCGGGAGG
GTTCGAGAAGGGGGGGCACCCCCCTTCGGCGTGCGCGGTCACGCGCCAGGGCGCAGCCCTGGTTAAAAACAA
GGTTTATAAATATTGGTTTAAAAGCAGGTTAAAAGACAGGTTAGCGGTGGCCGAAAAACGGGCGGAAACCCT
TGCAAATGCTGGATTTTCTGCCTGTGGACAGCCCCTCAAATGTCAATAGGTGCGCCCCTCATCTGTCAGCACTC
TGCCCCTCAAGTGTCAAGGATCGCGCCCCTCATCTGTCAGTAGTCGCGCCCCTCAAGTGTCAATACCGCAGGGC
ACTTATCCCCAGGCTTGTCCACATCATCTGTGGGAAACTCGCGTAAAATCAGGCGTTTTCGCCGATTTGCGAGG
CTGGCCAGCTCCACGTCGCCGGCCGAAATCGAGCCTGCCCCTCATCTGTCAACGCCGCGCCGGGTGAGTCGGC
CCCTCAAGTGTCAACGTCCGCCCCTCATCTGTCAGTGAGGGCCAAGTTTTCCGCGAGGTATCCACAACGCCGGC
GGCCGCGGTGTCTCGCACACGGCTTCGACGGCGTTTCTGGCGCGTTTGCAGGGCCATAGACGGCCGCCAGCCC
AGCGGCGAGGGCAACCAGCCCGGTGAGCGTCGGAAAGGCGCTGGAAGCCCCGTAGCGACGCGGAGAGGGG
CGAGACAAGCCAAGGGCGCAGGCTCGAGTCGCAGCACGACATAGCCGGTTCTCGCAAGGACGAGAATTTCCC
TGCGGTGCCCCTCAAGTGTCAATGAAAGTTTCCAACGCGAGCCATTCGCGAGAGCCTTGAG

ScelLacI

TCCACGCTAGATCTGAATTGTAGGGATAACAGGGTAATGAATTGAGCCAGCACAATTGTGAGCGGATAACAAT

LacI LexA$_{BT}$
TAAAAGTCAGAAATTGTGAGCGGATAACAATTAAAGACGGAGCATATGCAAATCGAATAAATGTTCGATTTAA
LexA$_{BT}$ LacI
ATCGAATAAATGTTCGATTTTATGGTACCCGCGCAAATTGTGAGCGGATAACAATTAGCAAGACAGAATTGTG
LacI SceI
AGCGGATAACAATTCTGGGAATTCTAGGGATAACAGGGTAATACTTCGG

Supplementary Table S4. Proteins of the purified Pdu compartments identified by mass spectrometry.

Supplementary Table S5. Percentage of reads aligned to LacI or LacI-LexA operators-containing 600 bp DNA segments, or 600 bp segments from the host *E. coli* JW0336-1 chromosome, or resident plasmids. Columns indicate different plasmids in *E. coli* strain JW0336-1. Rows show the percentage of reads aligned to random 600 bp segments from the chromosome, pACBSR derivatives (pOH), pRW derivatives, or to the cloned 600 bp segment containing the LacI operator sites (*lacO*) or the LacI and LexA sites (pRW-LexA). The percentage of reads per 600 bp segment is calculated with respect to all reads in the sample of the selected strain. Shown are the values from the two biological replicates.

E. coli JW0336-1	pRW-(*lacO*)$_5$ pOH	pRW-(*lacO*)$_5$ pOH	pRW-(*lacO*)$_5$ pOHΔPduD	pRW-(*lacO*)$_5$ pOHΔPduD	pRW-(*lacO*)$_5$ pOHΔTwin-Strep-tag	pRW-(*lacO*)$_5$ pOHΔTwin-Strep-tag
	Precentage of reads aligned to 600 bp region					
Chromosome	0.0053	0.0063	0.0108	0.0107	0.0074	0.0099
pOH	2.9412	2.2859	0.9420	1.0122	2.3350	1.3051
pRW	0.3013	0.4220	0.0075	0.0027	0.0983	0.0572
600 bp (*lacO*)$_{4-8}$	2.0954	3.8223	0.0231	0.0044	0.8107	0.2029

E. coli JW0336-1	pRW-(*lacO*)$_8$ pOH	pRW-(*lacO*)$_8$ pOH	pRW-(*lacO*)$_8$ pOH ΔLacI	pRW-(*lacO*)$_8$ pOH ΔLacI	pRW-LexA pOHΔLacI	pRW-LexA pOHΔLacI
	Precentage of reads aligned to 600 bp region					
Chromosome	0.0075	0.0092	0.0095	0.0103	0.0089	0.0093
pOH	1.3222	1.4526	1.7771	1.3326	2.2128	1.9889
pRW	1.1566	0.2247	0.1315	0.1318	0.0207	0.0253
600 bp (*lacO*)$_{4-8}$	17.9879	2.2943	0.0493	0.0905	0.0016	0.0076

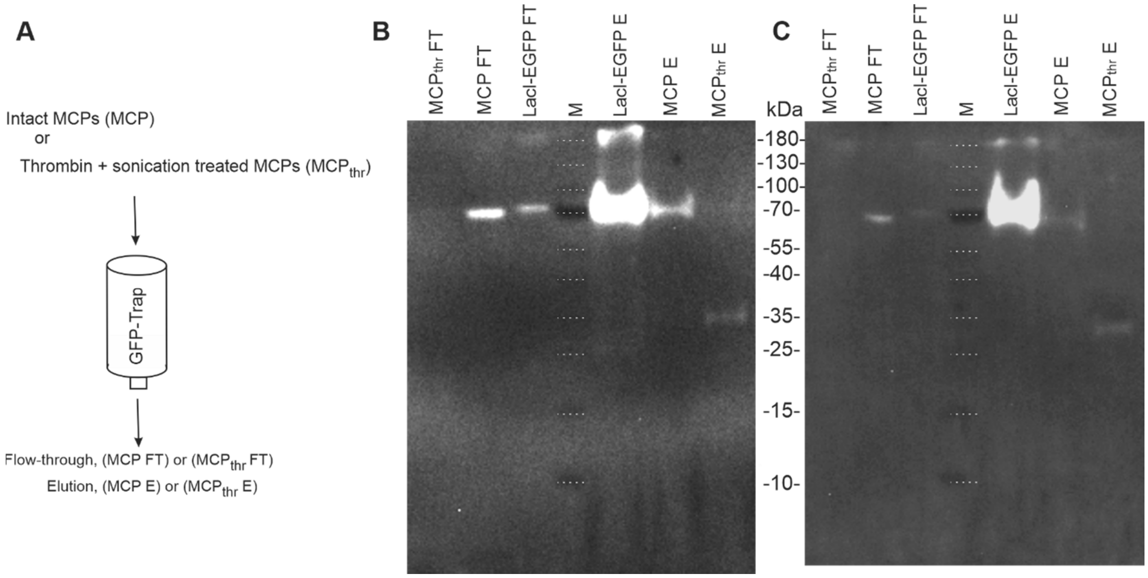

Supplementary Figure S1. Immunoprecipitation of $PduD_{(1\text{-}18)}$-LacI-EGFP fusion protein and Western blotting for EGFP. MCPs isolated from *E. coli* strain JW0336 carrying plasmid pOH were used in the analysis. (A) Schematic representation of purification step of Pdu MCPs, intact (MCP) or treated with thrombin and sonicated to release the MCPs content (MCP_{thr}), using GFP-Trap. Flow-through (FT) and elution fraction (E) were analysed for EGFP by Western blot (B, C, show two biological replicates; panel B shows the uncropped version of Figure 3E). $PduD_{(1\text{-}18)}$-LacI-EGFP (67.4 kDa) purified with Ni chelate affinity was used as a positive control. It is worth noting that purified $PduD_{(1\text{-}18)}$-LacI-EGFP carried an N-terminal His tag. Line M shows the PageRuler prestained protein ladder, the protein bands of which are also marked by white dotted lines (Thermo Fisher Scientific, USA). In intact MCP but not in broken MCP samples, EGFP signal corresponding to the size of $PduD_{(1\text{-}18)}$-LacI-EGFP was detected in the flow-through fractions, which shows that $PduD_{(1\text{-}18)}$-LacI-EGFP was captured inside MCPs.

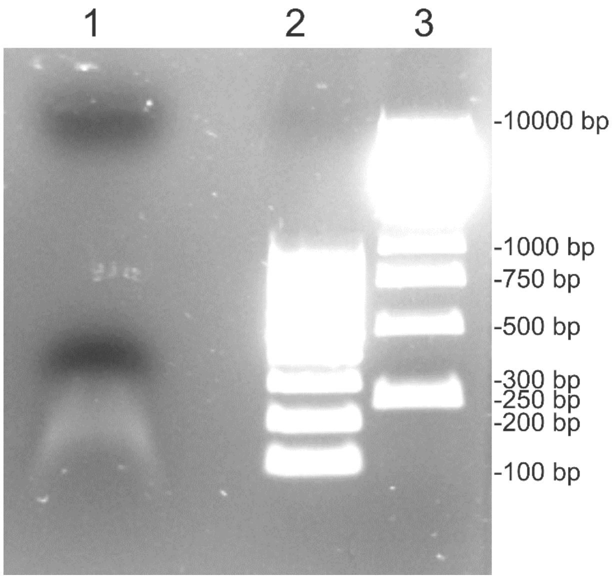

Supplementary Figure S2. The precipitated DNA (line 1) released by the proteinase from the affinity isolated Pdu-LacI microcompartments and resolved on the agarose gel which was stained by ethidium bromide. The microcompartments were isolated from the *E. coli* strain JW0336 carrying plasmids pOH and pRW-(*lacO*)$_5$. Lines 2 and 3 present the GeneRuler 100 bp DNA ladder and 1 kbp DNA ladder (Thermo Fisher Scientific, USA), respectively.

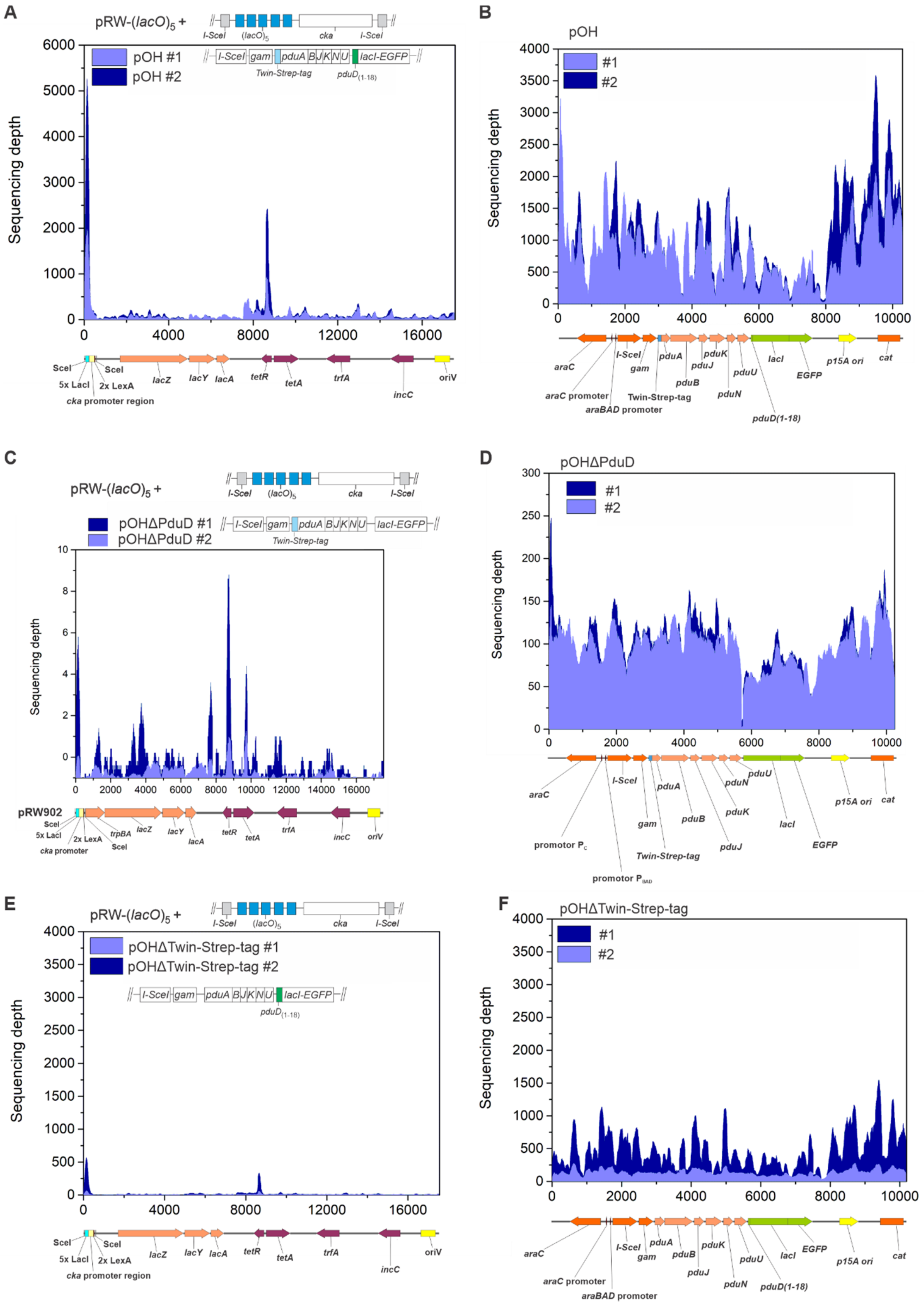

Supplementary Figure S3 Continued on next page.

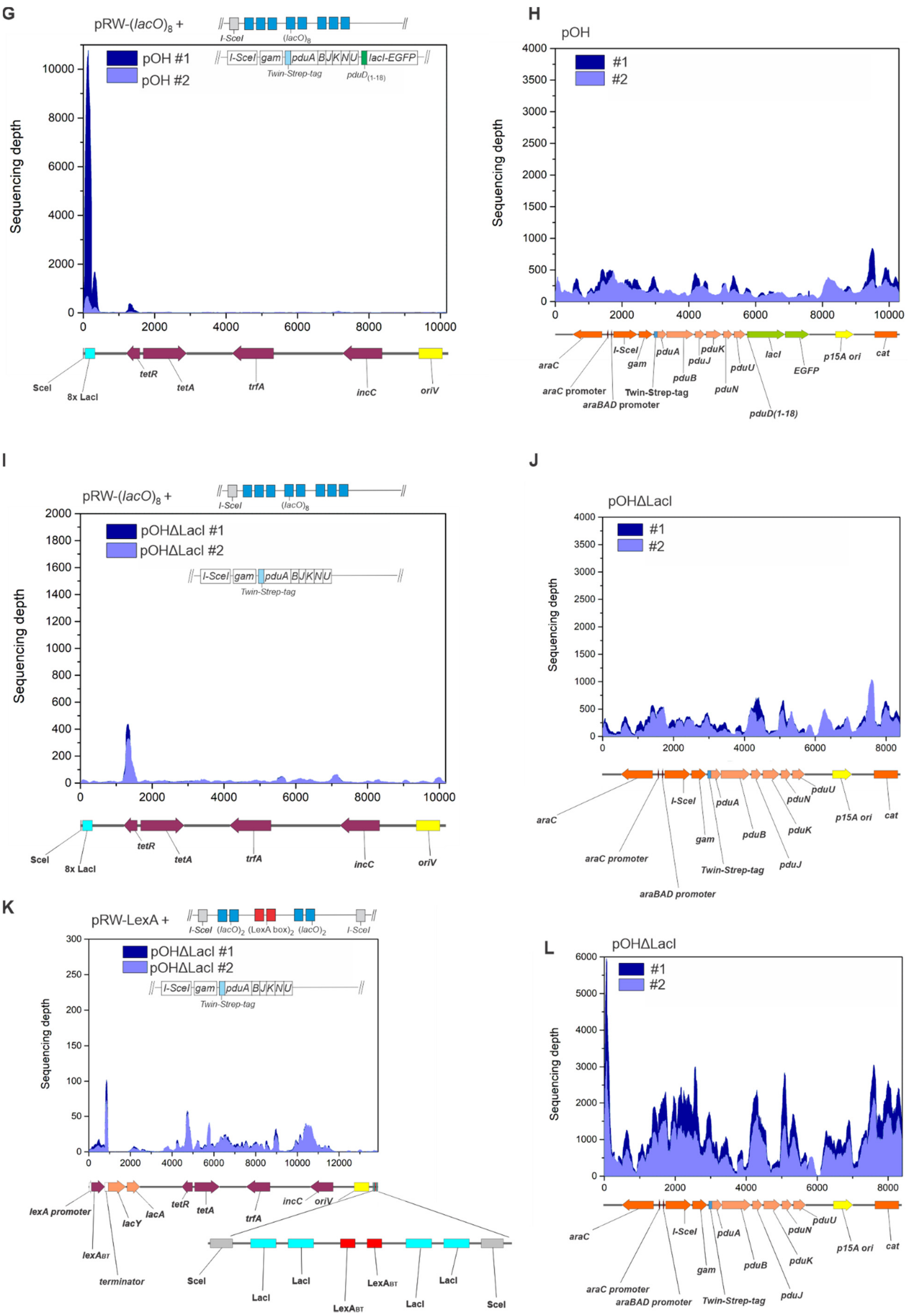

Supplementary Figure S3. Sequencing coverage of the two repeats shown in Figures 3, 4

and 5 (A, B) protocol run in the strain carrying pRW-(*lacO*)$_5$ and pOH, (C, D) in the strain carrying pRW-(*lacO*)$_5$ and pOHΔPduD, (E, F) in the strain carrying pRW-(*lacO*)$_5$ and pOHΔTwin-Strep-tag, (G, H) in the strain carrying pRW-(*lacO*)$_8$ and pOH, (I, J) in the strain carrying pRW-(*lacO*)$_8$ and pOHΔLacI or (K, L) in the strain carrying pRW-LexA and pOHΔLacI respectively. In contrast to the pRW plasmid derivatives, both pOH variants had similar average read coverage over most of their length and showed similar patterns of sequencing peaks. Note that the pRW derivatives carry a low-copy number replicon, RK2, reported to have approximately two copies, and the pACBSR, a medium-copy number replicon, p15A, with approximately nine copies per cell (5). Since the difference in template DNA concentration after the amplification, emulsion PCR step in the applied sequencing protocol is even potentiated by an undetermined multiple, we did not consider the template copy number difference between the two plasmids. We assume that we obtain pOH, pOHΔPduD, pOHΔLacI and pOHΔTwin-Strep-tag reads mapping through the plasmids as the transcription and translation processes are coupled in bacteria. We hypothesize that self-assembly of the Pdu compartment occurs in close spatial proximity to the coding plasmid. To complete the formation of the Pdu compartment, host nucleases presumably degrade the plasmid that may interfere with the shell assembly, resulting in the packaging of random DNA fragments.

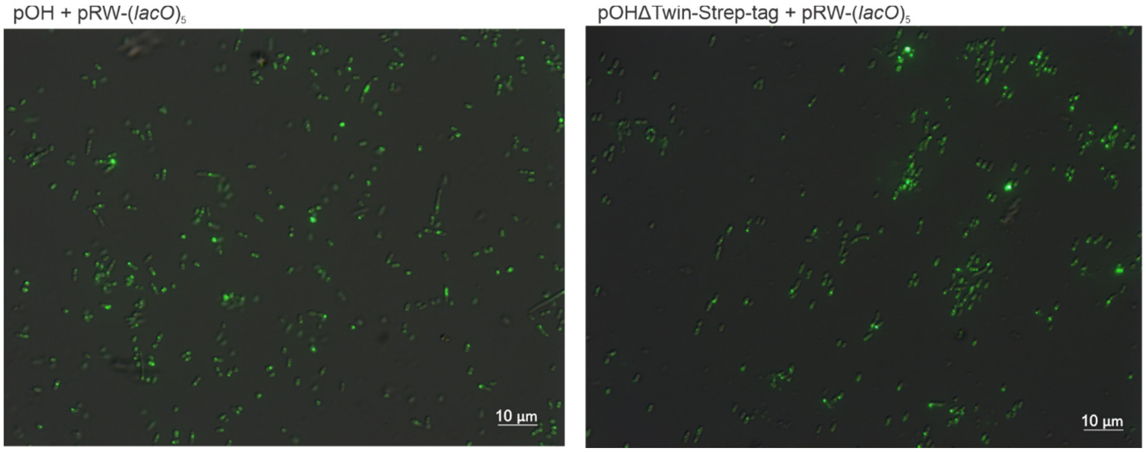

Supplementary Figure S4. Fluorescence microscopy micrographs of strains carrying two plasmids, as marked above the micrograph. Images were acquired an hour after induction of the protocol with L-arabinose.

A

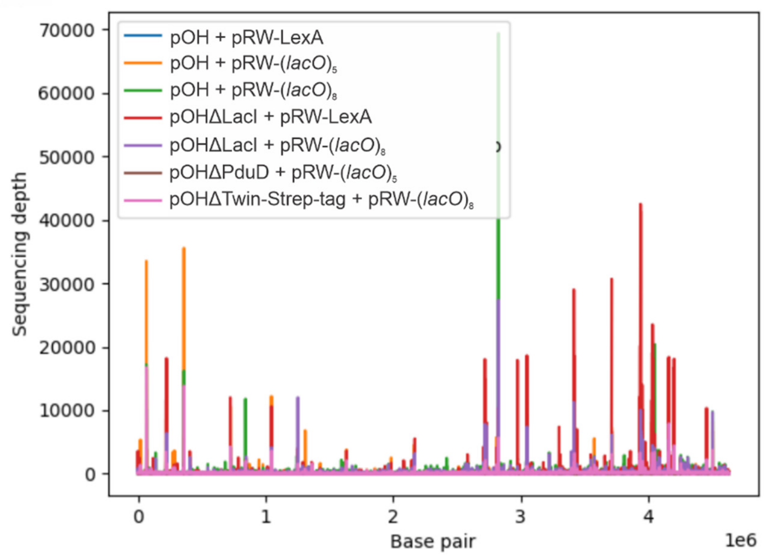

B

Genomic location (bp)		Gene
66500	67800	*araC* + promoter region
224129	225535	*rrlH*, *rrfH*
355837	361773	*cynX*, *lacA*, *lacY*,
407305	407937	*araJ*
526725	526795	*allS*
724608	724722	*ybfA*
848171	848339	*mntS* + promoter region
1047215	1047356	*ymcE* + promoter region
1254273	1254441	*ychH*
1311328	1312556	*trpB*
1484650	1484830	*gapC*
1546903	1546940	*yddM*
2170493	2170630	*gatY*
2225494	2225551	*cdd*
2825562	2825829	*norR* + promoter region
3049338	3049559	*ssrS*
3398219	3398769	*yrdE* + promoter region
4085289	4085617	*frvB*
4161484	4161890	*rrfB*
4201853	4203089	*rrlE*, *rrfE*
4254616	4255328	*dnaB*
4457303	4457482	promoter region of *mgtA*
4503588	4504027	*fecB*
4582404	4582833	*tsr*

Supplementary Figure S5. (A) Sequencing coverage of the chromosome for the strains carrying the following: (i) pRW-LexA and pOH or (ii) pRW-(*lacO*)$_5$ and pOH or (iii) pRW-(*lacO*)$_8$ and pOH or (iv) pRW-LexA and pOHΔLacI or (v) pRW-(*lacO*)$_8$ and pOHΔLacI or (vi) pRW-(*lacO*)$_5$ and pOHΔPduD or (vii) pRW-(*lacO*)$_5$ and pOHΔTwin-Strep-tag plasmids. (B) Location of highly sequenced regions on the chromosomes of the strains tested. Also marked is a gene or promoter region that was highly sequenced.

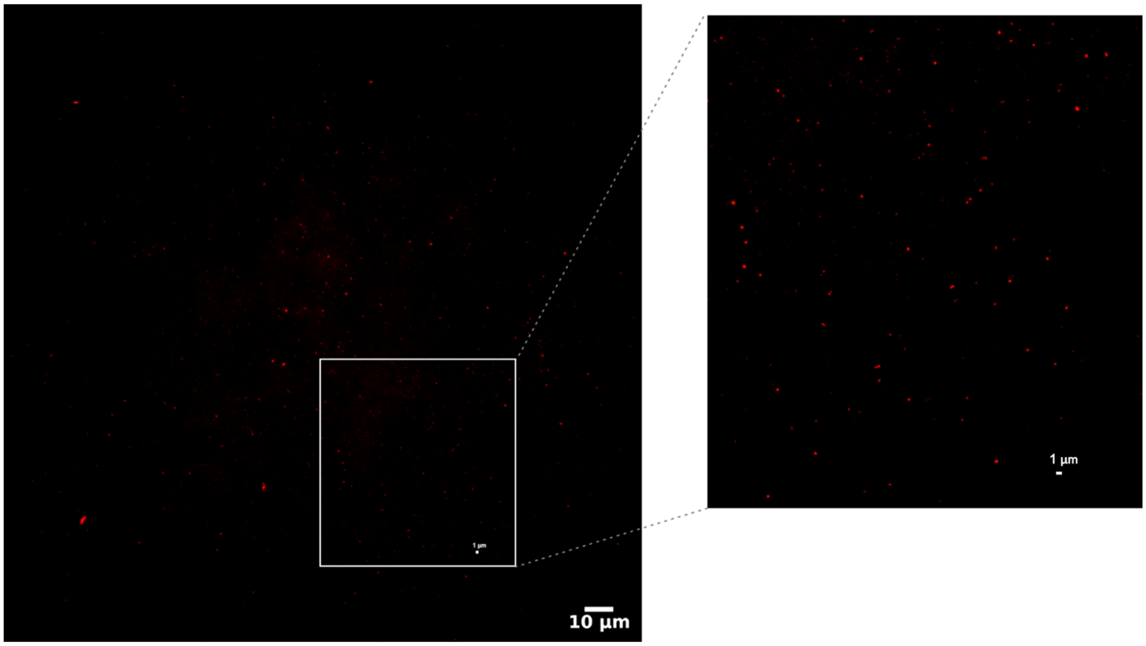

Supplementary Figure S6. Fluorescence micrographs of affinity purified Pdu compartments isolated from the strain carrying the plasmids pRW-LexA-mCherry and pOH-S.

References:

1. Herring,C.D., Glasner,J.D. and Blattner,F.R. (2003) Gene replacement without selection: regulated suppression of amber mutations in *Escherichia coli*. *Gene*, **311**, 153–163.

2. Parsons,J.B., Frank,S., Bhella,D., Liang,M., Prentice,M.B., Mulvihill,D.P. and Warren,M.J. (2010) Synthesis of empty bacterial microcompartments, directed organelle protein incorporation, and evidence of filament-associated organelle movement. *Mol. Cell*, **38**, 305–315.

3. Baba,T., Ara,T., Hasegawa,M., Takai,Y., Okumura,Y., Baba,M., Datsenko,K.A., Tomita,M., Wanner,B.L. and Mori,H. (2006) Construction of *Escherichia coli* K-12 in-frame, single-gene knockout mutants: the Keio collection. *Mol Syst Biol*, **2**, 2006 0008.

4. Butala,M., Busby,S.J. and Lee,D.J. (2009) DNA sampling: a method for probing protein binding at specific loci on bacterial chromosomes. *Nucleic Acids Res*, **37**, e37.

5. Jahn,M., Vorpahl,C., Hübschmann,T., Harms,H. and Müller,S. (2016) Copy number variability of expression plasmids determined by cell sorting and Droplet Digital PCR. *Microb. Cell Fact.*, **15**, 1-12.
